# Synthesis of Novel Stilbene–Coumarin Derivatives and Antifungal Screening of *Monotes kerstingii*-Specialized Metabolites Against *Fusarium oxysporum*

**DOI:** 10.3390/antibiotics9090537

**Published:** 2020-08-25

**Authors:** Ghislain Wabo Fotso, Bathelemy Ngameni, Thomas E. Storr, Bonaventure Tchaleu Ngadjui, Sibongile Mafu, G. Richard Stephenson

**Affiliations:** 1 Department of Organic Chemistry, Faculty of Science University of Yaoundé I, P.O. Box 812 Yaoundé, Cameroon; ghis152001@gmail.com (G.W.F.); ngadjuibt@yahoo.fr (B.T.N.); 2 Department of Biochemistry and Molecular Biology, University of Massachusetts, Amherst, MA 01003, USA; 3 Department of Pharmacognosy and Pharmaceutical Chemistry, Faculty of Medicine and Biomedical Sciences, University of Yaoundé I, P.O. Box 1364 Yaoundé, Cameroon; 4 School of Chemistry, University of East Anglia, Norwich Research Park, Norwich NR4 7TJ, UK; T.Storr@uea.ac.uk (T.E.S.); G.R.Stephenson@uea.ac.uk (G.R.S.)

**Keywords:** *Monotes kerstingii*, antifungal activity, *Fusarium oxysporum*, semisynthesis, stilbene–coumarins

## Abstract

*Fusarium* is one of the most toxigenic phytopathogens causing diseases and reduced agricultural productivity worldwide. Current chemical fungicides exhibit toxicity against non-target organisms, triggering negative environmental impact, and are a danger to consumers. In order to explore the chemical diversity of plants for potential antifungal applications, crude extract and fractions from *Monotes kerstingii* were screened for their activity against two multi-resistant *Fusarium oxysporum* strains: *Fo32931* and *Fo4287*. Antifungal activity was evaluated by the determination of minimum inhibitory concentration (MIC) by broth dilution of fermentative yeasts using kinetic OD_600 nm_ reading by a spectrophotometer. The *n*-butanol fraction showed the best activity against *Fo4287*. We screened eleven previously reported natural compounds isolated from different fractions, and a stilbene–coumarin 5-[(1*E*)-2-(4-hydroxyphenyl)ethenyl]-4,7-dimethoxy-3-methyl-2*H*-1-benzopyran-2-one (**1**) was the most active compound against both strains. Compound **1** was employed as a nucleophile with a selection of electrophilic derivatizing agents to synthesize five novel stilbene–coumarin analogues. These semisynthetic derivatives showed moderate activity against *Fo32931* with only prenylated derivative exhibiting activity comparable to the natural stilbene–coumarin (**1**), demonstrating the key role of the phenolic group.

## 1. Introduction

**Figure 1:**
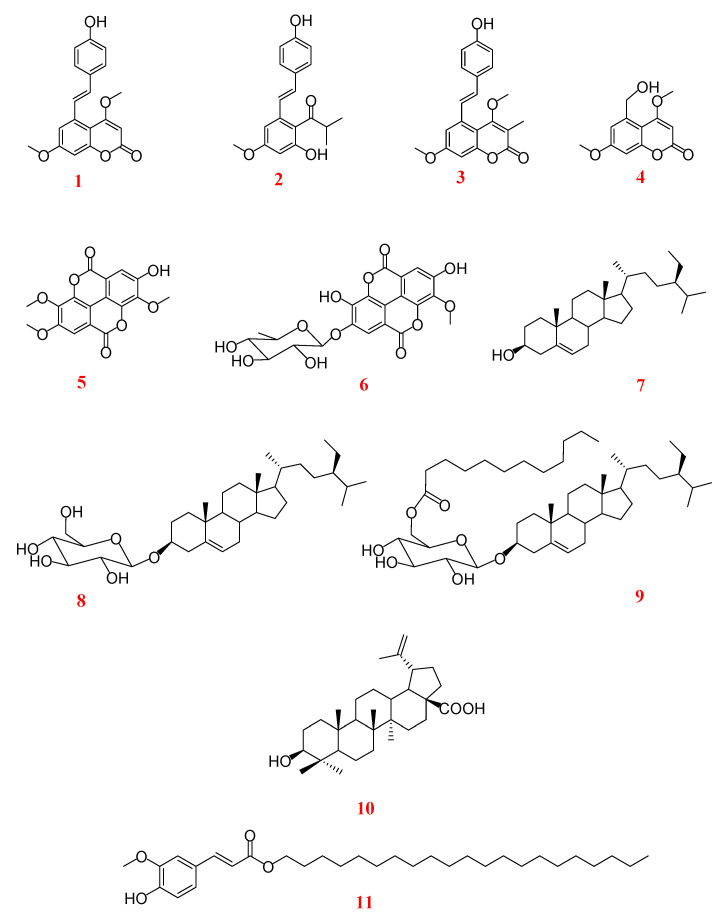
Structures of specialized metabolites **1**–**11** isolated from *Monotes kerstingii* [15].

*Fusarium* is a cross kingdom fungal pathogen with a detrimental influence on the quality, quantity, and profitability of agricultural production and simultaneously incurs opportunistic fungal infections in humans which pose an increasing threat to public health. In agricultural crops, these phytopathogens are persistent in avoiding plant defenses, resulting in diseases and quality losses that amount to billions of US dollars annually [1,2]. The management of plants infected by *Fusarium* through the use of chemical fungicides has proven to exhibit toxicity to non-target organisms, cause environmental damage, and present a danger to consumers. More than 100 species of *Fusarium* have been identified among which ten species complexes are pathogenic to humans causing diseases such as onychomycosis, endophthalmitis, as well as skin and musculoskeletal infections with immunocompromised patients being mostly affected [3]. Highly invasive fusariosis is commonly related to hepatitis and meningitis and is responsible for over one million new cases of blindness annually [4]. Additionally, clinically relevant *Fusarium* species are resistant to almost all currently used antifungals including azoles, echinocandins, and polyenes. There is a continuous need to search for new and efficient therapeutic alternatives against this fungal pathogen from natural sources such as medicinal plants and to provide an eco-friendly alternative to the use of pesticides [5,6]. It is estimated that approximately 25% of the active pharmaceutical agents prescribed in the United States originate from plant sources [7]. Moreover, natural products often possess more ‘drug-like’ features compared to molecules from combinatorial chemistry alone in terms of functional groups, polarity, three-dimensionality, and architectural diversity [8,9]. Therefore, the continued exploration of plants for lead compounds in combination with synthetic chemistry to improve their properties is an important avenue towards identifying compounds with potent antibacterial and antifungal properties. However, to reach the drug criterion, natural active compounds are often modified to produce semisynthetic derivatives to improve both physical and biological properties. Based on this approach, an antifungal drug, anidulafungin, was synthesized from echinocandin B originally isolated from a natural source [8,10]. The antifungal potential of plant extracts and fractions against *Fusarium oxysporum* has been explored in previous research [2,6,11]. As part of our ongoing search for novel antimicrobial and antifungal drug candidates from natural sources [12,13,14], we undertook a phytochemical study of the leaf and stem of the African shrub *Monotes kerstingii*, which led to the isolation of several specialized metabolites (Figure 1) [15]. Herein, we report the antifungal activity of the crude extract, fractions, and natural specialized metabolites from the stem bark of *M. kerstingii* against two *Fusarium oxysporum* strains: *Fo32931*, pathogenic to humans, and *Fo4287*, pathogenic to plants. Chemical modifications were carried out on the most active compound and the antifungal properties of the resultant semisynthetic derivatives were evaluated. 

## 2. Results and Discussion

### 2.1. Antifungal Activity of Crude Extract, Fractions, and Specialized metabolites from Monotes kerstingii against Fo32931 and Fo4287

Crude extract from *M. kerstingii* stem bark (MKS) together with hexane (HEX), ethyl acetate (EA), and *n*-butanol (BUT) fractions (MKSHEX, MKSEA, and MKSBUT) were screened for their antifungal activity against two *F. oxysporum* strains: *Fo32931*, pathogenic to humans, and *Fo4287*, pathogenic to plants (Table 1). Their antifungal activity was evaluated by cell growth curves. Potato dextrose broth (PDB) was used as a negative control and nystatin with an inhibitory concentration of 50 µg/mL was used as a positive control (Figure S1). The resultant minimum inhibitory concentration (MIC) was classified as strong (<100 µg/mL), moderate (100–500 µg/mL), weak (500–1000 µg/mL), or inactive (>1000 µg/mL) [16]. The crude extract, MKS, showed weak activity against both strains. MKSHEX exhibited moderate activity against *Fo4287* with a MIC of 331 µg/mL but was inactive against *Fo32931*. MKSEA was totally inactive against *Fo32931* and moderately active against *Fo4287* (MIC = 171 µg/mL). Strikingly, MKSBUT was strongly active against *Fo4287* with an MIC of 23 µg/mL, which is two times more than nystatin, the positive control (MIC = 50 µg/mL), but moderately active against *Fo32931* (MIC = 448 µg/mL). The presence of active compounds in the *n*-butanol suggests that active fractions/compounds against *Fusarium* spp. are more typically polar than non-polar. This result is in accordance with those recently published by Khan et al. (2018) and Nefzi et al. (2017), and it demonstrates the highest activity of *n*-butanol fraction compared to other less polar fractions [17,18]. 

Eleven natural compounds which were isolated from the above-mentioned fractions were screened for their antifungal activity against both *Fo32931* and *Fo4287* including two stilbene–coumarins (**1**, **2**), a stilbene (**3**), a coumarin (**4**), two ellagic acid derivatives (**5**, **6**), three steroids (**7**–**9**), a triterpene (**10**), and a cinnamate (**11**) (Figure 1). Compounds **2**, **7**, and **9**–**11** were isolated from the *n*-hexane fraction; **1**–**5** and **8** from the ethyl acetate fraction; and **1** and **6** from the *n*-butanol fraction [15]. In general, the pure compounds were more active against *Fo32931* where eight were moderately active, two weakly active, and one inactive. Against *Fo4287*, one compound exhibited marginally strong activity, six were moderately active, one weakly active, and three inactive (Table 1). The overall results of the pure compounds demonstrate a strain-dependent variation where the plant pathogen strain *Fo4287* was more resistant to *M. kerstingii*-specialized metabolites than the human pathogen strain, *Fo32931*. 

The stilbene–coumarin (**1**)—5-[(1*E*)-2-(4-hydroxyphenyl)ethenyl]-4,7-dimethoxy-3-methyl-2*H*-1-benzopyran-2-one—was the most actively tested compound with marginal strong activity against *Fo4287* (MIC = 96 µg/mL) and *Fo32931* (MIC = 116 µg/mL). Compound **2**, a natural alkylated derivative of (**1**) at C-3 of the coumarin ring showed moderate activity in both strains, demonstrating the importance of the free hydrogen H-3 of the double bond Δ^3,4^ in the antifungal activity of compound **1** against *F. oxsporum*. In comparison to compound **1**, the monomers stilbene (**3**) and coumarin (**4**) displayed reduced activity (Table 1). This was in accordance with the previously published results by Montagner et al. (2008) and Wu et al. (2008) who demonstrated the antifungal effect of coumarins against various fungal strains including *F. oxysporum* [19,20]. Ellagic acid (**5**) and its derivative (**6**) had marginally strong activity against *Fo32931* with a MIC of 98 µg/mL and 116 µg/mL, respectively, and exhibited strain-dependent activity with moderate reduced activity towards *Fo4287* (180 µg/mL and 361 µg/mL). Ellagic acid and ellagitannins have also been identified as active principles of pomegranate peel extract against *Fusarium* wilt of tomato plants [21]. The steroid **7** and its acylated derivative **9**, both isolated from the hexane fraction, showed moderate activity against *Fo32931* but were inactive against *Fo4287*. This result is consistent with data obtained by Mbambo et al. (2012) on *Fusarium verticilloides* for the same class of compounds [22]. Interestingly, compound **8**, a glycosylated derivative of **7**, exhibited significant increased activity two times greater the activity of compound **7** against both strains. It is postulated that after hydrolysis of the glycosylated compound, the glucose is absorbed and subsequently inhibits hyphal growth of yeast. The fungicidal action of the sugar unit is due to the effect on cell wall synthesis by inhibiting β-1,3-*D*-glucan synthase in fungal cell membranes [23,24]. The triterpene (**10**) and cinnamate (**11**) were weakly or inactive against both strains. 

Considering the interactions between compounds in different fractions and their effects on antifungal activity, it is observed that MKSHEX was moderately active against *Fo4287*, whilst the pure compounds **7**, **9**–**11** isolated from this fraction were inactive on this strain (with exception of **2** which was moderately active), demonstrating their synergistic effect against *Fo4287* or an as yet unidentified compound is responsible for the apparent activity. On the other hand, MKSHEX was inactive against *Fo32931*, but the above-mentioned pure compounds individually were moderately active, demonstrating an antagonistic effect in this fraction against *Fo32931*. Similarly, although MKSEA was totally inactive against *Fo32931*, the majority of compounds **2**–**4** and **8**, isolated from this fraction, were moderately active against this strain. In addition, the most active compounds **1** and **5** were isolated from the ethyl acetate fraction. Similar results demonstrating the weak activity of the hexane and ethyl acetate fractions of plant extracts were obtained by Ahmad et al., 2011, and Sunita et al., 2012 [25,26]. Finally, the strong antifungal activity of the *n*-butanol fraction on *Fo4287* could be explained by the co-presence of compounds **1** and **6** having a synergistic effect in that fraction. Our results show a strain-dependent variation where, in general, the crude extract and fractions had greater efficacy towards *Fo4287*, whereas for *Fo32931*, the results clearly demonstrate that activity increases with compound purity.

### 2.2. Alkylation of Compound ***1***: Semisynthesis of Allylated, Propargylated, Prenylated, and Benzylated Derivatives ***1a***–***e***

**Scheme 1:**
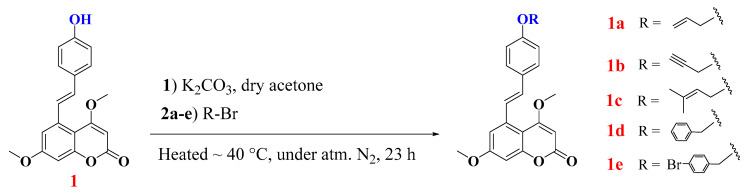
Synthesis of semisynthetic compounds **1a**–**e**.

Having identified the natural stilbene–coumarin (**1**), a compound with marginal strong MIC against both strains, a number of analogues of **1** were synthesized with an objective to improve its inhibitory activity. As phenolic groups have previously been demonstrated to exhibit antifungal activity [27,28], modifications were focused on the phenolic group to assess its importance and also to explore the structure–activity relationship of variously modified natural stilbene–coumarins. We performed a semi-synthesis of the most active compound **1** into its derivatives **1a**–**e** under weakly basic conditions as shown in Scheme 1. These chemical transformations produced *O*-allylated (**1a**), *O*-propargylated (**1b**), *O*-prenylated (**1c**), and *O*-benzylated (**1d**,**e**) synthesized products via one-pot nucleophilic substitution.

#### 2.2.1. Characterization of Compounds **1a**–**e**

The structures of the semisynthetic derivatives were established from spectral data, mainly HR-ESI-MS, 1D and 2D NMR, and by comparison with literature data.

**Figure 2:**
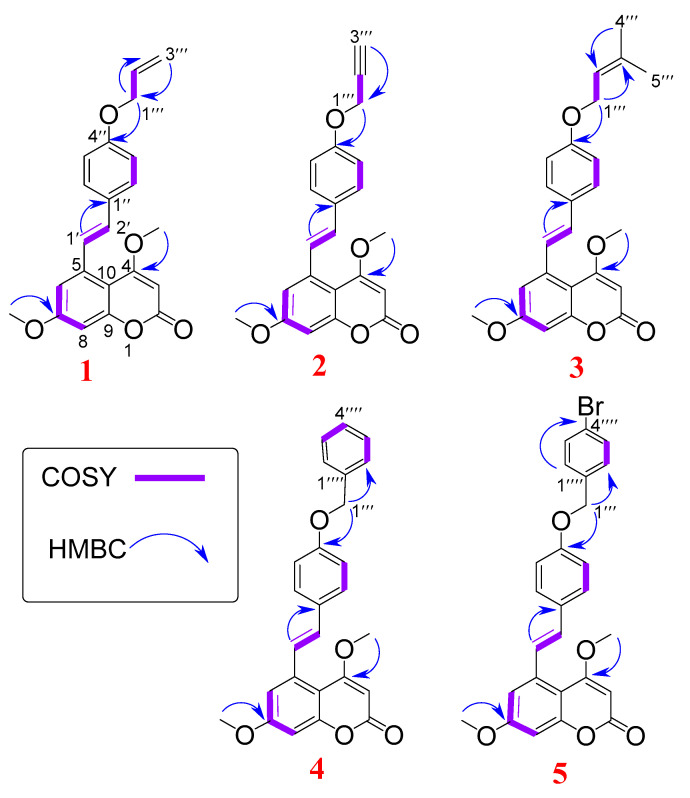
COSY and HMBC correlations of compounds **1a**–**e**.

Compound **1a** is a white amorphous solid soluble in chloroform. The HR-ESI-MS spectrum showed a pseudo molecular ion peak [M + H]^+^ at *m/z* 365.1427 (calcd. for C_22_H_21_O_5_^+^, 365.1389) corresponding to the molecular formula C_22_H_20_O_5_ with 13 degrees of unsaturation. The IR absorption spectrum of **1a** showed the characteristic bands at 1729, 1603, 1509, and 1257 cm^−1^, respectively, ascribed to a carbonyl group, ethylenic and aromatic double bonds, and ether functionality [29]. Its ^1^H NMR spectrum (Table 2) in conjunction with ^13^C NMR [15,30] in which trans-ethylenic protons were observed at δ_H_/δ_C_ 6.70 (d, *J* = 16.0 Hz, 1H)/131.4 and 7.68 (d, *J* = 16.0 Hz, 1H)/126.7; an AA’BB’ system of a para-substituted aromatic ring at δ_H_/δ_C_ 6.83 (d, *J* = 8.7 Hz, 2H)/115.1 and δ_H_/δ_C_ 7.35 (d, *J* = 8.7 Hz, 2H)/127.9, while the AB system of two aromatic protons H-6 and H-8 resonated at δ_H_/δ_C_ 6.87 (d, *J* = 2.3 Hz, 1H)/111.7 and δ_H_/δ_C_ 6.66 (d, *J* = 2.3 Hz, 1H)/100.0. The two methoxy groups at C-4 and C-7 were observed as singlets at δ_H_/δ_C_ 3.88/56.3 and 3.80/55.7, respectively. The absence of a broad absorption band on the IR spectrum of **1a** for the OH group at around 3300–3500 cm^−1^ suggested a substitution at C-4″. This was confirmed by the presence of the signals of an oxyallyl group [31] on the ^1^H and ^13^C NMR of **1a** as a doublet of an oxymethylene at δ_H_ 4.50 (*J* = 2.6 Hz, H-1‴)/68.9; a multiplet at δ_H_ 5.99 (H-2‴); and a set of two diastereotopic protons of an exomethylene carbon at δ_H_ 5.36 dd (*J* = 1.5 and 17.3 Hz) and δ_H_ 5.24 d (*J* = 11.8 Hz). The location of the allyl group at C-4″ was confirmed by the HMBC correlation (Figure 2) between the oxymethylene protons at δ_H_ 4.50 and C-4″ at δ_C_ 157.5. Based on this evidence, compound **1a** was identified as 5-[(1*E*)-2-(4-prop-2-enyloxyphenyl)ethenyl]-4,7-dimethoxy-3-2*H*-1-benzopyran-2-one.

Similarly, the synthesized compound **1b** was obtained as white amorphous solid soluble in chloroform. Its molecular formula was established as C_22_H_18_O_5_ by the interpretation of its HR-ESI-MS (positive mode) showing the protonated molecular ion peak [M + H]^+^ at *m/z* 363.1268 (calcd. for C_22_H_19_O_5_^+^, 363.1232). Its IR spectrum showed signals of a triple bond at 2129 cm^−1^, C=O at 1715 cm^−1^, C=C at 1600 and 1508 cm^−1^, as well as C-O-C at 1247 and 1152 cm^−1^. The ^1^H and ^13^C NMR spectra of **1b** showed high similarity with those of **1a** (Table 2 and Table 3) suggesting that **1b** is a stilbene–coumarin with the same skeleton as **1a**. However, the signals of the oxyallyl group in **1a** were replaced by those of an oxypropargyl group in **1b**, most notably a doublet at δ_H_ 2.48 (d, *J* = 2.4)/75.7 characteristic of the methine proton H-3‴ of a propargyl group. In the ^13^C spectrum, C-1‴ and C-2‴ appeared at 55.9 and 78.4 ppm, respectively, in accordance with literature data [32,33]. The location of this substituent at C-4″ was confirmed by the analysis of the HMBC spectrum of **1b** on which clear correlations were observed between H-1‴ at δ_H_ 4.66 ppm and C-4″ at δ_C_ 157.5 ppm (Figure 2). Compound **1b** was thus identified as 5-[(1*E*)-2-(4-prop-2-ynyloxyphenyl)ethenyl]-4,7-dimethoxy-3-2*H*-1-benzopyran-2-one.

Compound **1c** was obtained as white leaf solid soluble in chloroform. The HR-ESI-MS spectrum (positive mode) of **1c** showed the protonated molecular ion peak [M + H]^+^ at *m/z* 393.1716 (calcd. C_24_H_25_O_5_^+^, 393.1702) compatible with the molecular formula C_24_H_24_O_5_ with 13 degrees of unsaturation. As with **1a** and **1b**, compound **1c** showed characteristic signals of the stilbene–coumarin skeleton (Table 2 and Table 3). Further analysis of the ^1^H and ^13^C NMR of **1c** revealed the presence of an oxyprenyl group at C-4″ [34,35] characterized by two singlets of three protons each at δ_H/C_ 1.73/25.9 and 1.68/18.2 ascribed to the vinylic methyl groups; a triplet (*J =* 2.7 Hz) integrating as one proton assigned to the vinylic proton H-2‴ at δ_H_ 5.43, while the oxymethylene H-1‴ resonated as a doublet (*J* = 6.7 Hz) at δ_H_ 4.46. The presence of this substituent which was located at C-4″ (Figure 2) was confirmed the ^13^C spectrum of **1c** (Table 3). Compound **1c** was identified as 5-[(1*E*)-2-(4-prenyloxyphenyl)ethenyl)]-4,7-dimethoxy-3-2*H*-1-benzopyran-2-one.

Compound **1d** was obtained as white amorphous solid soluble in chloroform. Its molecular formula was deduced as C_26_H_22_O_5_ by the interpretation of its HR-ESI-MS (positive mode) showing the protonated molecular ion peak [M + H]^+^ at *m/z* 415.1544 (calcd. C_26_H_23_O_5_^+^, 415.1545). Its IR spectrum showed important bands at 1734, 1602, 1510, 1256, and 1153 cm^−1^ attributed to carbonyl, ethyl, and aromatic double bonds and ether functions [35]. ^1^H and ^13^C NMR spectra and data (Table 2 and Table 3) of **1d** were similar to the previously described derivatives. In addition, the ^1^H NMR spectrum of **1d** showed, in addition to the signals of the aromatic protons of the stilbene–coumarin, another set of five aromatic protons as multiplets between 7.25 and 7.40 ppm. This information combined with the presence of a highly deshielded oxymethylene (H-1‴) as a singlet at δ_H/C_ 5.04/70.0 suggested the presence of a benzyl group [36] in compound **1d**. HMBC correlation (Figure 2) between the oxymethylene proton H-1‴ and C-4″ at δ_C_ 158.7 confirmed its location at C-4″ of the stilbene–coumarin. Compound **1d** was identified as 5-[(1*E*)-2-(4-benzyloxyphenyl)ethenyl)]-4,7-dimethoxy-3-2*H*-1-benzopyran-2-one.

Compound **1e** was obtained as white amorphous solid soluble in chloroform. Its molecular formula was established as C_26_H_21_BrO_5_ based on its HR-ESI-MS spectrum (positive mode) on which the pseudomolecular ion peak [M + H]^+^ was observed at *m/z* 493.0652 (calcd. C_26_H_22_BrO_5_^+^, 493.0651). The presence of a bromine atom in compound **1e** was confirmed by two peaks with a 1:1 ratio in the molecular ion region. Moreover, the IR spectrum displayed, in addition to the bands of **1c**, a characteristic band of C-Br at 819 cm^−1^ [37]. The NMR data (Table 2) were very similar to those of **1d** confirming a stilbene–coumarin moiety bearing a benzyl group, however, contrary to multiplets observed in the aromatic region between 7.25 and 7.40 ppm and assigned to a phenyl ring of the benzyl group. The ^1^H NMR spectrum of **1e** displayed an AA’BB’ system of a *para*-bromodisubstituted aromatic ring at δ_H/C_ [7.35 (2H, d, *J* = 8.5)/129.0 and 7.55 (2H, d, 8.5)/131.8]. The location of the *para*-bromobenzyl group was confirmed at C-4″ by the HMBC correlation between the oxymethylene H-1‴ at δ_H_ 5.09 and C-4″ at 158.5. Based on this evidence, the structure of compound **1e** was established to be 5-[(1*E*)-2-(4-bromobenzyloxyphenyl)ethenyl)]-4,7-dimethoxy-3-2*H*-1-benzopyran-2-one. 

#### 2.2.2. Antifungal Activity of Semi-Synthetic Derivatives against *Fo32931* and *Fo4287*

The semisynthetic derivatives (**1a**–**e**) were also assayed for their antifungal activity against *F. oxysporum* strains. The compounds exhibited moderate activity towards *Fo32931* which was contrary to the weak to no activity against *Fo4287* (Table 1). Inhibitory activity against *Fo32931* clearly indicates the influence of distinct chemical groups to antifungal activity. In detail, the prenylated derivative **1c** was the most active semisynthetic derivative (MIC = 103 µg/mL) with comparable activity to the most active natural compound **1** (MIC = 116 µg/mL), indicative of the importance of prenylation on antimicrobial activity. The addition of prenyl groups to molecules has been demonstrated to play a key role in antimicrobial activity as it increases the lipophilicity of the molecule, enhancing access, affinity, and interaction with the lipophilic membrane or by inhibiting RAS transduction [38,39,40]. Compounds **1a**,**b** and **e** exhibited moderate activity against *Fo32931* with a MIC of 250–500 µg/mL. Notably, bromination at the *para* position of the benzyl group in **1e** significantly increases the activity against both strains as compared only to the benzylated derivative compound **1d** that had weak activity (Scheme 1). As the semisynthetic compounds were in general less active than (**1**), it can be postulated that the phenolic group plays a key role in the antifungal activity of specialized metabolites against *F. oxysporum.* Phenolic-OH moiety increase hydrophilicity enhancing the ability of penetration and damaging yeast plasma membrane to achieve high antifungal activity [41]. 

## 3. Materials and Methods 

### 3.1. General Methods

IR spectra were recorded on KBr discs using a Perkin Elmer Spectrum 100 FT-IR-410 spectrometer; ν in cm^‒1^.^1^H-, ^13^C-NMR, DEPT, COSY, HSQC, NOESY, and HMBC spectra: Bruker AMX 500 instrument (at 500 and 125 MHz, respectively); in chloroform-d; δ in ppm rel. to Me_4_Si as internal standard, *J* in Hz. HR-ESI-MS APEXIII (Bruker Daltonik) 7 Tesla (ESI-FT-ICR-MS); in *m/z*. Spectra were processed using the computer software MestreNova 9.1. Column chromatography (CC) was performed over Merck silica gel 60, particle size between 0.043 and 0.063 mm in diameter, and porosity 230–400 mesh ASTM. Evaporation was performed using a BUCHI rotary evaporator under reduced pressure. Plant materials, extracts, and fractions were weighed on a Satorius OT12 electronic mass balance. Pure compounds or fractions were weighed on a Satorius BP221S electronic balance maximum rating 220 g d = 0.1 mg. Analytical TLCs were carried out on pre-coated silica gel 60 F254 aluminum sheets (0.25 mm layer, Merck). The chromatograms were visualized under UV light at 254 and 366 nm and with 10% H_2_SO_4_ spray, then heated.

### 3.2. Plant Material

The stem bark of *M. kerstingii* was collected in May 2018 in Touboro, a locality of the North Region of Cameroon, and a voucher specimen was deposited at the National Herbarium of Cameroon with the registration number N6661/SRFCAM.

### 3.3. Extraction of M. kerstingii Stem Barks and Isolation of Specialized Metabolites 

The air-dried and powdered stem bark of *M. kerstingii* (100 g) was sonicated in a mixture of ethanol/H_2_O (7:3) for 2 h. The mixture was filtered, and the solvent evaporated under reduced pressure to give 13.20 g of a brownish crude extract (MKS). A total of 10 g of MKS was dissolved in H_2_O and partitioned successively using (3 × 50 mL) each of hexane (MKSHEX, 0.32 g) and ethyl acetate (MKSEA, 1.236 g) and *n*-butanol (MKSBUT, 7.55 g). Specialized metabolites **1**–**11** were isolated from *M. kerstingii* roots as previously described [15].

### 3.4. Alkylation of Compound ***1***

In each experiment, 49.50 mg (0.15 mmol) of 5-[(1*E*)-2-(4-hydroxyphenyl)ethenyl]-4,7-dimethoxy-3-methyl-2*H*-1-benzopyran-2-one **1** were added to anhydrous K_2_CO_3_ (300 mg, 2.17 mmol, 15 eq) in dry acetone (10 mL) followed by various alkylbromide as follows: (a) allylbromide (0.50 mL, d = 1.40, 0.70 g, 5.78 mmol); (b) propargylbromide (0.50 mL, d = 1.38, 0.69 g, 5.80 mmol); (c) prenylbromide (0.50 mL, d = 1.29, 0.65 g, 4.33 mmol); (d) benzylbromide (0.50 mL, d = 1.44, 0.72 g, 4.21 mmol); (e) p-bromobenzylbromide (d = 1.85, 0.20 g, 0.80 mmol). The reaction mixture was heated at 40 °C under reflux for 23 h. TLC was used to monitor the alkylation. At the end of the reaction, the solvent was evaporated under reduced pressure. Each residue was suspended in water (40 mL) and then extracted with ethyl acetate (60 mL). The extract was dried by anhydrous Na_2_SO_4_. The ethyl acetate extract was then evaporated and purified through a silica gel column chromatography eluted with a mixture *n*-hexane-ethyl acetate of increasing polarity. Moreover, further purifications of the synthesized products were completed using the preparative TLC. Through this process, the semisynthetic derivatives were obtained as follows: compound **1a** (25.3 mg, 0.07 mmol, 46%, *R_f_* 0.40, silica gel, hexane-EtOAc, 7:3 *v*/*v*); compound **1b** (84.8 mg, 0.23 mmol, 90%, *R_f_* 0.30, silica gel, hexane-EtOAc, 3:2 *v*/*v*); compound **1c** (29.1 mg, 0.07 mmol, 49%, *R_f_* 0.50, silica gel, hexane-EtOAc, 3:2 *v*/*v*); compound **1d** (51.42 mg, 0.12 mmol, 82%, *R_f_* 0.31, silica gel, hexane-EtOAc, 7:3 *v*/*v*); compound **1e** (39.6 mg, 0.08 mmol, 53%, *R_f_* 0.43, silica gel, hexane-EtOAc, 4:1 *v*/*v*).

### 3.5. Antifungal Bioassay

**Yeasts isolates.***F. oxysporum* strains *Fo32931* and *Fo4287* were obtained from Ma Lab (University of Massachusetts-Amherst). The fungi were prepared from glycerol stocks. Each *F. oxysporum* strain was first grown on potato dextrose agar (PDA) media by inoculating 20 µL of stock at the center of the plate and incubating at 28 °C for three days. Fungi were then transferred and grown in PDB for two days. Spores were filtered using a miracloth, and 1 × 10^7^ spores/mL concentration were obtained using a hemocytometer. 

**Preparation of stock solutions.***M. kerstingii* crude extract stock solution (MKS) was prepared at 20 mg/mL. Fractions (MKSHEX, MKSEA, and MKSBUT) and pure compound stock solutions were prepared at 6 mg/mL, while nystatin at 1 mg/mL was used at positive control. Tested samples and control were dissolved in 50% DMSO. 

**Growth curve and determination of the MIC.** Serial dilutions were performed to achieve concentrations ranging from 1000 to 31.25 μg/mL from extracts and fractions, and from 300 to 18.75 µg/mL for pure compounds. Samples were tested against 10% *v*/*v* of 1 × 10^7^ spores/mL of *F. oxysporum* in a 96 well plate at the final concentration of 1 × 10^6^ spores/mL *F. oxysporum* in each plate. Ten percent *v*/*v* of 1 × 10^7^ spores/mL *F. oxysporum* was supplemented with PDB media to a final volume of 200 µL. For the positive control, 5% *v*/*v* of nystatin stock at the final concentration of 50 µg/mL was used. PDB was used as negative control. As *F. oxysporum* is sensitive to DMSO, its concentration was maintained at 2.5% throughout the screenings. Experiments were performed in duplicates and repeated three times for each experiment. The antifungal activities of crude extracts fractions and pure compounds were assessed by the inhibition growth curve through a kinetic reading of the optical density at 600 nm of the media over 24 h using a spectrophotometer (SpectraMax M2 Multilabel Microplate Reader). After subtraction of the OD of the blank, which consisted of non-inoculated wells that had been incubated together with the inoculated wells, from the OD of the inoculated wells, the inhibition percentages were calculated as defined by Campbell in 2010 [42] as Inhibition Percentage = 100 − [(OD*_avg sample_* − OD*_avg pos_*)/(OD*_avg neg_* − OD *_avg pos_*) × 100]. The minimal inhibitory concentration was determined by the lowest concentration of the sample achieving 100% of inhibition [43] using a linear regression in the program GraphPad Prism version 5.00 (GraphPad Software, San Diego, CA, USA) for Windows. The antifungal activity of the samples was analyzed as active or not active according Morales et al.’s criteria: strong/good activity (MIC < 100 µg/mL); moderate activity (100 < MIC < 500 µg/mL); weak activity (500 < MIC < 1000 µg/mL); no activity (MIC >1000 µg/mL) [16].

## 4. Conclusions

This research was designed to evaluate the potential of *M. kerstingii* stem bark crude extracts, fractions, natural metabolites from these fractions, and semisynthetic analogues of compound **1** against two multiresistant *F. oxysporum* strains (*Fo32931*, pathogenic to humans, and *Fo4287*, pathogenic to plants). Results revealed that crude stem bark from *M. kerstingii* has a weak activity against both strains. However, after fractionation, the *n*-butanol extract exhibited strong activity against *Fo4287*, two times more than nystatin, the positive control. Results also revealed that the stilbene–coumarin (**1**), as well as ellagic acid and its derivative (**5** and **6**) were the most active principle of this plant. However, ellagic acid derivatives showed significant activity only against *Fo32931*, whereas the stilbene–coumarin (**1**)—5-[(1*E*)-2-(4-hydroxyphenyl) ethenyl]-4,7-dimethoxy-3-methyl-2*H*-1-benzopyran-2-one—was active against both strains. Compared to the starting material **1**, which had marginal strong activity against both strains, the semisynthetic analogues were either only moderately active (against *Fo2391*) or inactive (against *Fo4287*), demonstrating the importance of the phenolic group on antifungal activity. Only the prenylated derivative **1c** exhibited an activity comparable to compound **1** against *Fo2391*, highlighting the key role of the prenyl group that is known to increase the antimicrobial activity of a molecule. In summary, this study reveals that the *n*-butanol fraction of *M. kerstingii* and its phytoconstituents have potential as natural environment-friendly fungi-toxicant against *F. oxysporum*. We identified stilbene–coumarin as a promising lead compound that can be further explored for improved efficacy through synergism with other compounds or further chemical transformations. 

## Figures and Tables

**Table 1:** Minimum inhibitory concentration (MIC) of crude extracts, fractions, as well as natural and hemisynthetic pure compounds against *Fo32931* and *Fo4287*.

Type	Code	*Fo32931* MIC (µg/mL)	*Fo4287* MIC (µg/mL)
Extract	MKS	841	679
Fractions	MKSHEX	>1000	331
	MKSEA	>1000	171
	MKSBUT	448	23
Hemisynthetic	1a	477	>1000
	1b	281	658
	1c	103	>1000
	1d	708	>1000
	1e	341	621
Natural	1	116	96
	2	337	286
	3	327	330
	4	553	273
	5	98	180
	6	116	361
	7	408	915
	8	174	457
	9	473	>1000
	10	>1000	>1000
	11	523	>1000
Pop	Nystatin	50	50

MKS: *M. kerstingii* stem bark; MKSHEX: *M. kerstingii* hexane fraction; MKSEA: *M. kerstingii* ethyl acetate fraction; MKSBUT: *M. kerstingii n*-butanol fraction. Strong/good activity (MIC: <100 µg/mL); moderate activity (MIC: 100–500 µg/mL); weak activity (MIC: 500–1000 µg/mL); no activity (MIC: >1000 µg/mL) [16].

**Table 2:** ^1^H NMR data of compounds **1a**–**e** [CDCl_3_; 500 MHz].

N°	Compound 1a δ_H_ (m, *J* in Hz)	Compound 1b δ_H_ (m, *J* in Hz)	Compound 1c δ_H_ (m, *J* in Hz)	Compound 1d δ_H_ (m, *J* in Hz)	Compound 1e δ_H_ (m, *J* in Hz)
**3**	5.49 (1H, s)	5.52 (1H, s)	5.49 (1H, s)	5.51 (1H, s)	5.62 (1H, s)
**4**	-	-	-	-	-
**5**	-	-	-	-	-
**6**	6.87 (1H, d, 2.6)	6.89 (1H, d, 2.4)	6.87 (1H, d, 2.6)	6.89 (1H, d, 2.6)	6.99 (1H, d, 2.5)
**7**	-	-	-	-	-
**8**	6.66 (1H, d, 2.5)	6.68 (1H, d, 2.6)	6.65 (1H, d, 2.6)	6.67 (1H, d, 2.6)	6.79 (1H, d, 2.5)
**9**	-	-	-	-	-
**10**	-	-	-	-	-
**1′**	7.68 (1H, d, 16.0)	7.71 (1H, d, 16.0)	7.67 (1H, d, 15.9)	7.69 (1H, d, 16.0)	7.80 (1H, d, 15.9)
**2′**	6.70 (1H, d, 16.0)	6.73 (1H, d, 16.0)	6.71 (1H, d, 15.9)	6.73 (1H, d, 16.0)	6.81 (1H, d, 15.9)
**1″**	-	-	-	-	-
**2″, 6″**	7.35 (2H, d, 8.7)	7.39 (2H, d, 8.6)	7.35 (2H, d, 8.7)	7.35 (2H, d, 8.8)	7.47 (2H, d, 8.7)
**4″**	-	-	-	-	-
**3″, 5″**	6.86 (2H, d, 8.7)	6.93 (2H, d, 8.8)	6.85 (2H, d, 8.7)	6.92 (2H, d, 8.8)	6.99 (2H, d, 8.7)
**1‴**	4.50 (2H, d, 2.6)	4.66 (2H, d, 2.4)	4.46 (2H, d, 6.7)	5.04 (2H, s)	5.09 (2H, s)
**2‴**	5.99 (1H, m)		5.43 (1H, t, 2.7)		
**3‴**	5.36 (1H, dd, 17.3; 1.5) 5.24 (1H, d, 11.8)	2.48 (1H, d, 2.4)			
**4‴**			1.73 (3H, s)		
**5‴**			1.68 (3H, s)		
**2⁗, 6⁗**				7.25–7.40 m	7.35 (2H, d, 8.5)
**3⁗, 5⁗**					7.55 (2H, d, 8.5)
**4⁗**					-
**4-OMe**	3.88 (3H, s)	3.92 (3H, s)	3.87 (3H, s)	3.90 (3H, s)	3.99 (3H, s)
**7-OMe**	3.80 (3H, s)	3.89 (3H, s)	3.80 (3H, s)	3.82 (3H, s)	3.92 (3H, s)

**Table 3:** ^13^C NMR data of compounds **1a**–**e** [CDCl_3_; 125 MHz].

N°	Compound 1a δ_C_ (m)	Compound 1b δc (m)	Compound 1c δc (m)	Compound 1d δc (m)	Compound 1e δc (m)
**2**	162.1 (s)	162.1 (s)	162.1 (s)	162.0 (s)	162.1 (s)
**3**	88.2 (d)	88.2 (d)	88.2 (d)	88.0 (d)	88.2 (d)
**4**	169.2 (s)	169.2 (s)	169.3 (s)	169.2 (s)	169.2 (s)
**5**	131.0 (s)	131.0 (s)	130.0 (s)	130.2 (s)	130.5 (s)
**6**	111.7 (d)	111.9 (d)	111.7 (d)	111.6 (d)	111.8 (d)
**7**	162.9 (s)	162.9 (s)	162.9 (s)	162.8 (s)	162.9 (s)
**8**	100.0 (d)	100.1 (d)	100.0 (d)	100.0 (d)	100.1 (d)
**9**	156.4 (s)	156.4 (s)	156.4 (s)	156.4 (s)	156.5 (s)
**10**	106.7 (s)	106.7 (s)	106.7 (s)	106.6 (s)	106.7 (s)
**1′**	126.7 (d)	127.2 (d)	126.5 (d)	126.7 (d)	127.0 (d)
**2′**	131.4 (d)	131.2 (d)	131.4 (d)	131.3 (d)	131.3 (d)
**1″**	138.8 (s)	138.8 (s)	138.9 (s)	138.9 (s)	138.9 (s)
**2″, 6″**	127.9 (d)	115.2 (d)	127.9 (d)	127.9 (d)	128.0 (d)
**4″**	157.5 (s)	157.5 (s)	158.9 (s)	158.7 (s)	158.5
**3″, 5″**	115.1 (d)	127.9 (d)	115.0 (d)	115.1 (d)	115.2 (d)
**1‴**	68.9 (t)	55.9 (t)	64.9 (t)	70.0 (t)	69.3
**2‴**	133.1 (d)	78.4 (s)	119.5 (d)		
**3‴**	117.4 (t)	75.7 (d)	138.4 (s)		
**4‴**			25.9 (q)		
**5‴**			18.2 (q)		
**1⁗**				136.7 (s)	135.9
**2⁗**				128.6 (d)	129.0
**3⁗**				127.9 (d)	131.8
**4⁗**				128.0 (d)	129.0
**5⁗**				127.9 (d)	131.8
**6⁗**				128.6 (d)	122.0
**4-OMe**	56.3 (q)	56.4 (q)	56.3 (q)	56.4 (q)	56.4
**7-OMe**	55.7 (q)	55.7 (q)	55.7 (q)	55.7 (q)	55.7

Spectroscopic data of compounds **1a**–**e**.

**5-[(1*E*)-2-(4-prop-2-enyloxyphenyl)ethenyl]-4,7-dimethoxy-3-2*H*-1-benzopyran-2-one (1a)**

White amorphous solid; IR (KBr) *ν_max_* (cm^−^^1^): 1729 (C=O), 1603 and 1509 (C=C), 1380 (Csp^3^-H), 1257 and 1150 (C-O); ^1^H, ^13^C NMR, see Table 1 and Table 2; HR-ESI-MS: *m/z* 365.1427 [M + H]^+^ (calcd. for C_22_H_21_O_5_^+^, 365.1389).

**5-[(1*E*)-2-(4-prop-2-ynyloxyphenyl)ethenyl]-4,7-dimethoxy-3-2*H*-1-benzopyran-2-one (1b)**

White amorphous solid; IR (KBr) *ν_max_* (cm^−1^): 2129 (C≡C), 1715 (C=O), 1600 and 1508 (C=C), 1378 (Csp^3^-H), 1247 and 1152 (C-O); ^1^H, ^13^C NMR, see Table 1 and Table 2; HR-ESI-MS: *m/z* 363.1268 [M + H]^+^ (calcd. for C_22_H_19_O_5_^+^, 363.1232).

**5-[(1E)-2-(4-prenyloxyphenyl)ethenyl)]-4,7-dimethoxy-3-2H-1-benzopyran-2-one (1c)**

White leaf solid; IR (KBr) *ν_max_* (cm^−^^1^): 1731 (C=O), 1603 and 1508 (C=C), 1381 (Csp^3^-H), 1251 and 1149 (C-O); ^1^H, ^13^C NMR, see Table 1 and Table 2; HR-ESI-MS: *m/z* 393.1716 [M + H]^+^ (calcd. C_24_H_25_O_5_^+^, 393.1702).

**5-[(1E)-2-(4-benzyloxyphenyl)ethenyl)]-4,7-dimethoxy-3-2H-1-benzopyran-2-one (1d)**

White amorphous solid; IR (KBr) *ν_max_* (cm^−^^1^): 1734 (C=O), 1602 and 1510 (C=C), 1385 (Csp^3^-H), 1256 and 1153 (C-O); ^1^H and ^13^C NMR, see Table 1 and Table 2; HR-ESI-MS: *m/z* 415.1544 [M + H]^+^ (calcd. C_26_H_23_O_5_^+^, 415.1545).

**5-[(1E)-2-(4-bromobenzyloxyphenyl)ethenyl)]-4,7-dimethoxy-3-2H-1-benzopyran-2-one (1e)**

White amorphous solid; IR (KBr) *ν_max_* (cm^−^^1^): 1731 (C=O), 1606 and 1510 (C=C), 1382 (Csp^3^-H), 1256 and 1152 (C-O), 819 (C-Br); ^1^H and ^13^C NMR, see Table 1 and Table 2; HR-ESI-MS: *m/z* 493.0652 [M + H]^+^ (calcd. C_26_H_22_BrO_5_^+^, 493.0651).

## Supplementary Materials

The following are available online at https://www.mdpi.com/2079-6382/9/9/537/s1, Figures S1–S49.

## References

[1] Shuping D.S.S., Eloff J.N. (2017). The use of plants to protect plants and food against fungal pathogens: A Review. Afr. J. Tradit. Complement. Altern. Med..

[2] Ramaiah A.K., Garampalli R.k.H. (2015). In vitro antifungal activity of plant extracts against Fusarium oxysporum f. sp. *lycopersici*. Asian J. Plant Sci. Res..

[3] Wickern G.M. (1993). Fusarium allergic fungal sinusitis. J. Allergy Clin. Immunol..

[4] Kredics L., Narendran V., Shobana C.S., Vágvölgyi C., Manikandan P., Indo-Hungarian Fungal Keratitis Working Group (2015). Filamentous fungal infections of the cornea: A global overview of epidemiology and drug sensitivity. Mycoses.

[5] Al-Hatmi A.M., Meis J.F., de Hoog G.S. (2016). Fusarium: Molecular Diversity and Intrinsic Drug Resistance. PLoS Pathog.

[6] Boulenouar N., Marouf A., Cheriti A., Belboukhari N. (2012). Medicinal Plants Extracts as Source of Antifungal Agents against *Fusarium oxysporum* f. sp. *Albedinis*. J. Agric. Sci. Technol..

[7] Newman D.J., Cragg G.M. (2020). Natural Products as Sources of New Drugs over the Nearly Four Decades from 01/1981 to 09/2019. J. Nat. Prod..

[8] Butler M.S. (2004). The role of natural product chemistry in drug discovery. J. Nat. Prod..

[9] Cowan M.M. (1999). Plant products as antimicrobial agents. Clin. Microbiol. Rev..

[10] Guo Z. (2017). The modification of natural products for medical use. Acta Pharm. Sin. B.

[11] Taffou J.B., Fokou H., Zeuko’o E.M., Tchokouaha L.R.T., Mfopa A.N., Kamdem M.S., Ngouana V., Kenfack I.F., Boyom F.F. (2017). Anti-yeast potential of some Annonaceae species from Camerronian biodiveristy. Int. J. Biol.Chem Sci..

[12] Mambe F.T., Na-Iya J., Fotso G.W., Ashu F., Ngameni B., Ngadjui B.T., Beng V.P., Kuete V. (2019). Antibacterial and Antibiotic Modifying Potential of Crude Extracts, Fractions, and Compounds from. Evid. Based Complement. Altern. Med..

[13] Kuete V., Wabo H.K., Eyong K.O., Feussi M.T., Wiench B., Krusche B., Tane P., Folefoc G.N., Efferth T. (2011). Anticancer activities of six selected natural compounds of some Cameroonian medicinal plants. PLoS ONE.

[14] Tabopda T.K., Fotso G.W., Ngoupayo J., Mitaine-Offer A.C., Ngadjui B.T., Lacaille-Dubois M.A. (2009). Antimicrobial dihydroisocoumarins from Crassocephalum biafrae. Planta Med..

[15] Fotso G.W., Mogue Kamdem L., Dube M., Fobofou S.A., Ndjie Ebene A., Arnold N., Tchaleu Ngadjui B. (2019). Antimicrobial secondary metabolites from the stem barks and leaves of Monotes kerstingii Gilg (Dipterocarpaceae). Fitoterapia.

[16] Morales G., Paredes A., Sierra P., Loyola L.A. (2008). Antimicrobial activity of three baccharis species used in the traditional medicine of Northern Chile. Molecules.

[17] Khan N., Martínez-Hidalgo P., Ice T.A., Maymon M., Humm E.A., Nejat N., Sanders E.R., Kaplan D., Hirsch A.M. (2018). Antifungal Activity of. Front. Microbiol..

[18] Nefzi A., Jabnoun-Khiareddine H., Abdallah R.A.B., Ammar N., Medimagh-Saidana S., Haouala R., Daami-Remadi M. (2017). Suppressing Fusarium Crown and Root Rot infections and enhancing the growth of tomato plants by *Lycium arabicum* Schweinf. Ex Boiss. extracts. S. Afr. J. Bot..

[19] Montagner C., de Souza S.M., Groposoa C., Delle Monache F., Smânia E.F., Smânia A. (2008). Antifungal activity of coumarins. Z. Naturforsch. C J. Biosci..

[20] Wu H.-S., Raza W., Liu D.-Y., Wu C.-L., Mao Z.-S., Xu Y.-C., Shen Q.-R. (2008). Allelopathic impact of applied coumarin on *Fusarium oxysporum* f.sp. *niveum*. World J. Microbiol. Biotechnol..

[21] Rongai D., Pulcini P., Pesce B., Milano F. (2017). Antifungal activity of pomegranate peel extract against fusarium wilt of tomato. Eur. J. Plant Pathol..

[22] Mbambo B., Odhav B., Mohanlall V. (2012). Antifungal activity of stigmasterol, sitosterol and ergosterol from *Bulbine natalensis* Baker (Asphodelaceae). J. Med. Plants Res..

[23] Guo J., Hu H., Zhao Q., Wang T., Zou Y., Yu S., Wu Q., Guo Z. (2012). Synthesis and antifungal activities of glycosylated derivatives of the cyclic peptide fungicide caspofungin. ChemMedChem.

[24] Moore D. (1981). Effects of hexose analogues on fungi: Mechanisms of inhibition and resistance. New Phytol..

[25] Ahmad B., Khan I., Bashir S., Azam S., Ali N. (2011). The antifungal, cytotoxic, antitermite and insecticidal activities of Zizyphus jujube. Pak. J. Pharm. Sci..

[26] Sunita P., Jha S., Pattanayak S.P., Mishra S. (2012). Antimicrobial activity of a halophytic plant *Cressa cretica* L.. J.Sci. Res..

[27] Teodoro G.R., Ellepola K., Seneviratne C.J., Koga-Ito C.Y. (2015). Potential Use of Phenolic Acids as Anti-Candida Agents: A Review. Front. Microbiol..

[28] Schöneberg T., Kibler K., Sulyok M., Musa T., Bucheli T.D., Mascher F., Bertossa M., Voegele R.T., Vogelgsang S. (2018). Can plant phenolic compounds reduce Fusarium growth and mycotoxin production in cereals?. Food Addit. Contam. Part A Chem. Anal. Control Expo Risk Assess..

[29] Heneczkowski M., Kopacz M., Nowak D., Kuźniar A. (2001). Infrared spectrum analysis of some flavonoids. Acta Pol. Pharm..

[30] Chavez D., Chai H.-B., Chagwedera T.E., Gao Q., Farnsworth N.R., Cordell G.A., Pezzuto J.M., Kinghorn A.D. (2001). Novel stilbenes isolated from the root bark of *Ekebergia benguelensis*. Tetrahedron Lett..

[31] Ngameni B., Fotso W.G., Ngachussi E., Poumale H., Ngadjui B., Shiono Y., Murayama T. (2014). Hemisynthesis and spectroscopic characterization of two novel *O*-allylated benzophenones from *Garcinia punctata* Oliv. (Clusiaceae). Asian J. Chem..

[32] Hearn M. (1975). Carbon-13 chemical shifts of some propargyl alchohol derivatives. Tetrahedron.

[33] Uliniuc A., Popa M., Drockenmuller E., Boisson F., Leonard D., Hamaide T. (2013). Toward tunable amphiphilic copolymers via CuAAC click chemistry of oligocaprolactones onto starch backbone. Carbohydr. Polym..

[34] Ngnintedo D., Fotso G.W., Kuete V., Nana F., Sandjo L.P., Karaosmanoğlu O., Sivas H., Keumedjio F., Kirsch G., Ngadjui B.T. (2016). Two new pterocarpans and a new pyrone derivative with cytotoxic activities from. Chem. Cent. J..

[35] Xiao Y., Lee I.S. (2018). Microbial transformation of quercetin and its prenylated derivatives. Nat. Prod. Res..

[36] Al-Douh M.H., Hamid S.A., Osman H. (2008). 1D and 2D NMR Studies of benzyl *O*-vanillin. Indones. J. Chem..

[37] Badertscher M., Buhlmann P., Pretsch E. (2009). Structure Determination of Organic Compounds.

[38] Yang X., Yang J., Jiang Y., Yang H., Yun Z., Rong W., Yang B. (2016). Regiospecific synthesis of prenylated flavonoids by a prenyltransferase cloned from Fusarium oxysporum. Sci. Rep..

[39] Alhassan A.M., Abdullahi M.I., Uba A., Umar A. (2014). Prenylation of aromatic secondary metabolites: A new frontier for development of novel drugs. Trop. J. Pharm. Res..

[40] Botta B., Vitali A., Menendez P., Misiti D., Monache G.D. (2005). Prenylated flavonoids: Pharmacology and Biotechnology. Curr. Med. Chem..

[41] Konuk H.B., Ergüden B. (2020). Phenolic -OH group is crucial for the antifungal activity of terpenoids via disruption of cell membrane integrity. Folia Microbiol. (Praha).

[42] Campbell J. (2011). High-throughput assessment of bacterial growth inhibition by optical density measurements. Curr. Protoc. Chem. Biol..

[43] Rodriguez-Tudela J., Barchiesi F., Bille J., Chryssanthou E., Cuenca-Estrella M., Denning D., Donnelly J., Dupont B., Fegeler W., Moore C. (2002). Method for the determination of minimum inhibitory concentration (MIC) by broth dilution of fermentative yeasts. Clin. Microbiol. Infect..

