# Supplementary material for: Synthesis of Novel Stilbene–Coumarin Derivatives and Antifungal Screening of Monotes kerstingii-Specialized Metabolites Against Fusarium oxysporum
Source: Antibiotics (Basel). 2020 Aug 25;9(9):537. doi: 10.3390/antibiotics9090537 (PMC7558447; doi:10.3390/antibiotics9090537)
Supplement: Supplementary file 1 [file antibiotics-09-00537-s001.pdf]

SUPPLEMENTAL DATA

Synthesis of novel stilbene-coumarins derivatives, and antifungal screening of *Monotes kerstingii* specialized metabolites against *Fusarium oxysporum*

Ghislain Wabo Fotso[1,4], Bathelemy Ngameni[2,3]*, Thomas E. Storr[3], Bonaventure Tchaleu Ngadjui[1], Sibongile Mafu[4]and G. Richard Stephenson[3]**

[1] Department of Organic Chemistry, Faculty of Science University of Yaoundé 1, P.O. Box 812 Yaoundé, Cameroon

[2] Department of Pharmacognosy and Pharmaceutical Chemistry, Faculty of Medicine and Biomedical Sciences, University of Yaoundé I, P.O. Box 1364, Yaoundé, Cameroon

[3] School of Chemistry, University of East Anglia, Norwich Research Park, Norwich NR4 7TJ, UK

[4] Department of Biochemistry and Molecular Biology, University of Massachusetts

Correspondence: bath_ngameni@yahoo.fr; Tel.: +237 690 230 281 (B.N)
smafu@umass.edu, Tel.: +1 413 545 4083 (S.M)

Received: date; Accepted: date; Published: date

Abstract: *Fusarium* is one of the most toxigenic phytopathogen causing diseases and reduced agricultural productivity worldwide. Current chemical fungicides exhibit toxicity against non-target organisms, triggering negative environmental impact and danger to consumers. In order to explore the chemical diversity of plants for potential antifungal applications, crude extract and fractions from *Monotes kerstingii* were screened for their activity against two multi-resistant *Fusarium oxysporum* strains: *Fo32931* and *Fo4287*. Antifungal activity was evaluated by the determination of minimum inhibitory concentration (MIC) by broth dilution of fermentative yeasts using kinetic $OD_{600\ nm}$ reading by a spectrophotometer. The *n*-butanol fraction showed the best activity against *Fo4287*. We screened eleven previously reported natural compounds isolated from different fractions and a stilbene-coumarin 5-[(1*E*)-2-(4-hydroxyphenyl)ethenyl]-4,7-dimethoxy-3-methyl-2*H*-1-benzopyran-2-one (**1**) was the most active compound against both strains. Compound **1** was employed as a nucleophile with a selection of electrophilic derivatizing agents to synthesize five novel stilbene-coumarin analogues. These semisynthetic derivatives showed moderate activity against *Fo32931* with only the prenylated derivative exhibiting activity comparable to the natural stilbene coumarin (**1**), demonstrating the key role of the phenolic group.

Keywords: *Monotes kerstingii*, antifungal activity, *Fusarium oxysporum*, semisynthesis, stilbene-coumarins.

Figure S1: Inhibition growth curves of PDB neat (negative control) and Nystatin (positive control) at different concentrations against *Fo32931* (A) and *Fo4287* (B)……3
Figure S2: MIC of *Monotes kerstingii* stem bark crude extract against *Fo32931* (A) and *Fo4287* (B)……4
Figure S3: MIC of hexane fraction against *Fo4287* (A), ethyl acetate fraction against *Fo4287* (B), n-butanol fraction against *Fo32931* (C) and against *Fo4287* (D)……4
Figure S4: MIC of the most active natural compounds: stilbene-coumarin 1 (A) and (B); ellagic acid derivatives 5 and 6 (C) and (D) against Fo32931 and Fo4287……5
Figure S5: MIC of the hemisynthetic prenylated compound **1c** against *Fo32931*……5
Figure S6: HRESIMS spectrum of compound **1a**……6
Figure S7: IR spectrum of compound **1a**……6
Figure S8: ^{1}H NMR ($CDCl_3$, 500 MHz) spectrum of compound **1a**……7
Figure S9: COSY spectrum of compound **1a**……7
Figure S10: ^{13}C NMR spectrum ($CDCl_3$, 125 MHz) of compound **1a**……8
Figure S11: DEPT 135 spectrum of compound **1a**……8
Figure S12: HSQC spectrum of compound **1a**……9
Figure S13: HMBC spectrum of compound **1a**……9
Figure S14: ROESY spectrum of compound **1a**……10
Figure S15: HRESIMS spectrum of compound **1b**……10
Figure S16: IR spectrum of compound **1b**……11
Figure S17: ^{1}H NMR spectrum ($CDCl_3$, 500 MHz) of compound **1b**……11
Figure S18: COSY spectrum of compound **1b**……12
Figure S19: ^{13}C NMR spectrum ($CDCl_3$, 125 MHz) of compound **1b**……12
Figure S20: DEPT 135 spectrum of compound **1b**……13
Figure S21: HSQC spectrum of compound **1b**……13
Figure S22: HMBC spectrum of compound **1b**……14
Figure S23: NOESY spectrum of compound **1b**……14
Figure S24: HRESIMS spectrum of compound **1c**……15
Figure S25: IR spectrum of compound **1c**……15
Figure S26: ^{1}H NMR spectrum ($CDCl_3$, 500 MHz) of compound **1c**……16
Figure S27: COSY spectrum of compound **1c**……16
Figure S28: ^{13}C NMR spectrum ($CDCl_3$, 125 MHz) of compound **1c**……17
Figure S29: DEPT 135 spectrum of compound **1c**……17
Figure S30: HSQC spectrum of compound **1c**……18
Figure S31: HMBC spectrum of compound **1c**……18
Figure S32: NOESY spectrum of compound **1c**……19
Figure S33: HRESIMS spectrum of compound **1d**……19
Figure S34: IR spectrum of compound **1d**……20
Figure S35: ^{1}H NMR spectrum ($CDCl_3$, 500 MHz) of compound **1d**……20
Figure S36: COSY spectrum of compound **1d**……21
Figure S37: ^{13}C NMR spectrum ($CDCl_3$, 125 MHz) of compound **1d**……21
Figure S38: DEPT 135 spectrum of compound **1d**……22
Figure S39: HSQC spectrum of compound **1d**……22
Figure S40: HMBC spectrum of compound **1d**……23
Figure S41: NOESY spectrum of compound **1d**……23
Figure S42: HRESIMS spectrum of compound **1e**……24
Figure S43: IR spectrum of compound **1e**……24
Figure S44: ^{1}H NMR spectrum ($CDCl_3$, 500 MHz) of compound **1e**……25
Figure S45: COSY spectrum of compound **1e**……25
Figure S46: ^{13}C NMR spectrum ($CDCl_3$, 125 MHz) of compound **1e**……26
Figure S47: DEPT 135 spectrum of compound **1e**……26
Figure S48: HSQC spectrum of compound **1e**……27
Figure S49: HMBC spectrum of compound **1e**……27

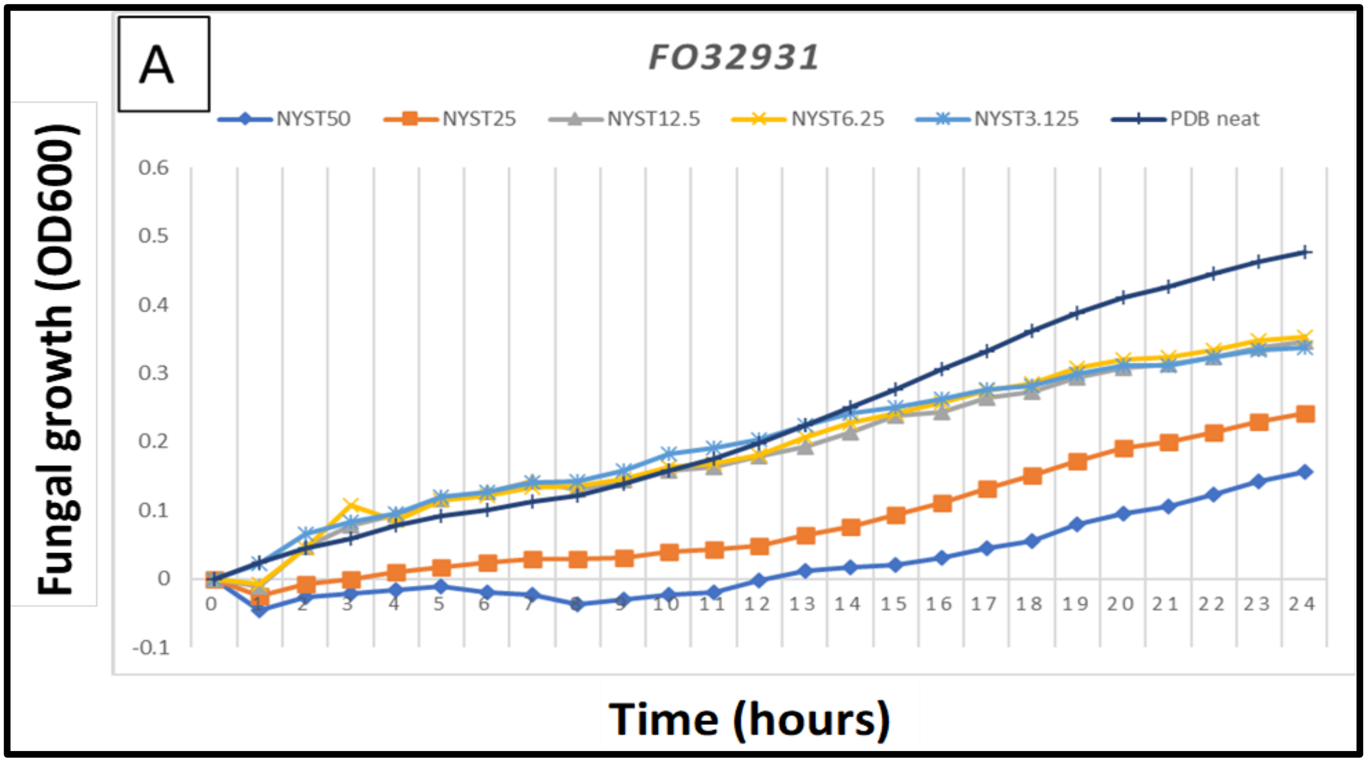

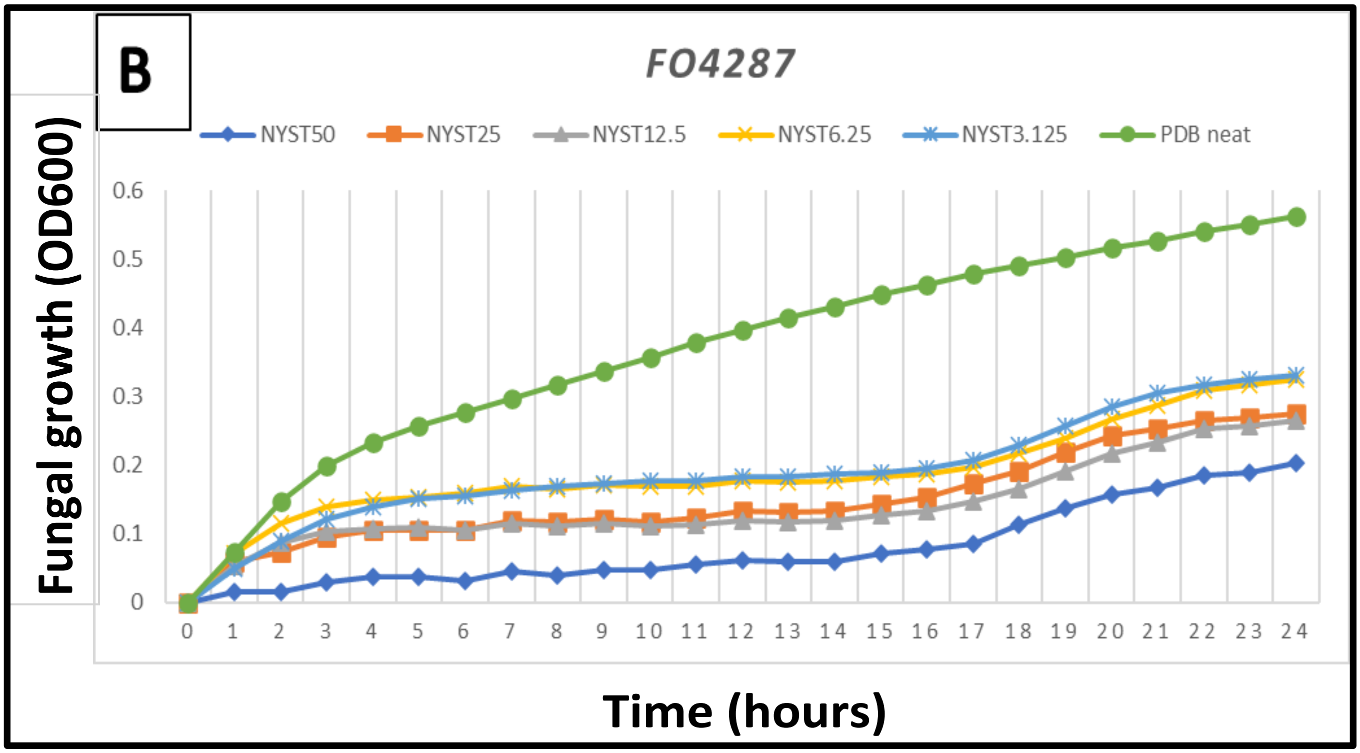

Figure S1: Fungal growth curves of *Fo32931* (A) and *Fo4287* (B) in PDB neat (negative control) and Nystatin at different concentrations (positive control)

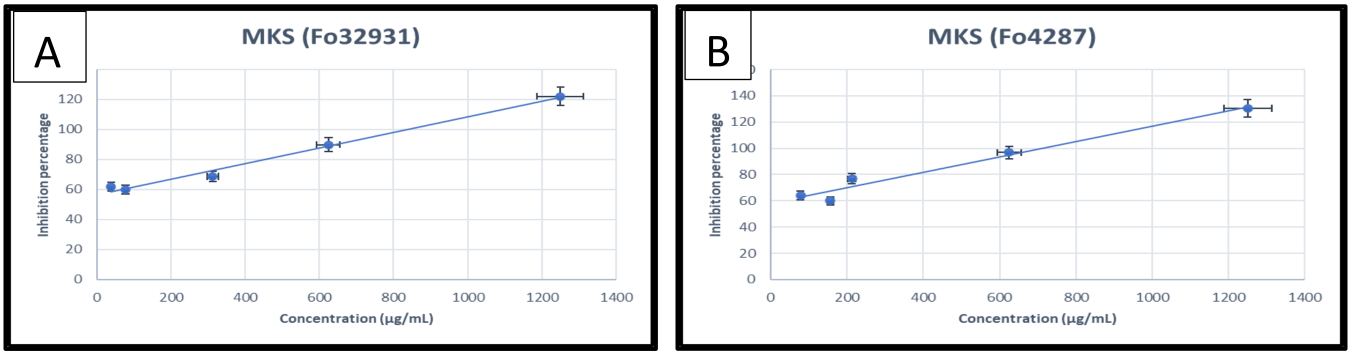

Figure S2: MIC of *Monotes kerstingii* stem bark crude extract against *Fo32931* (A) and *Fo4287* (B)

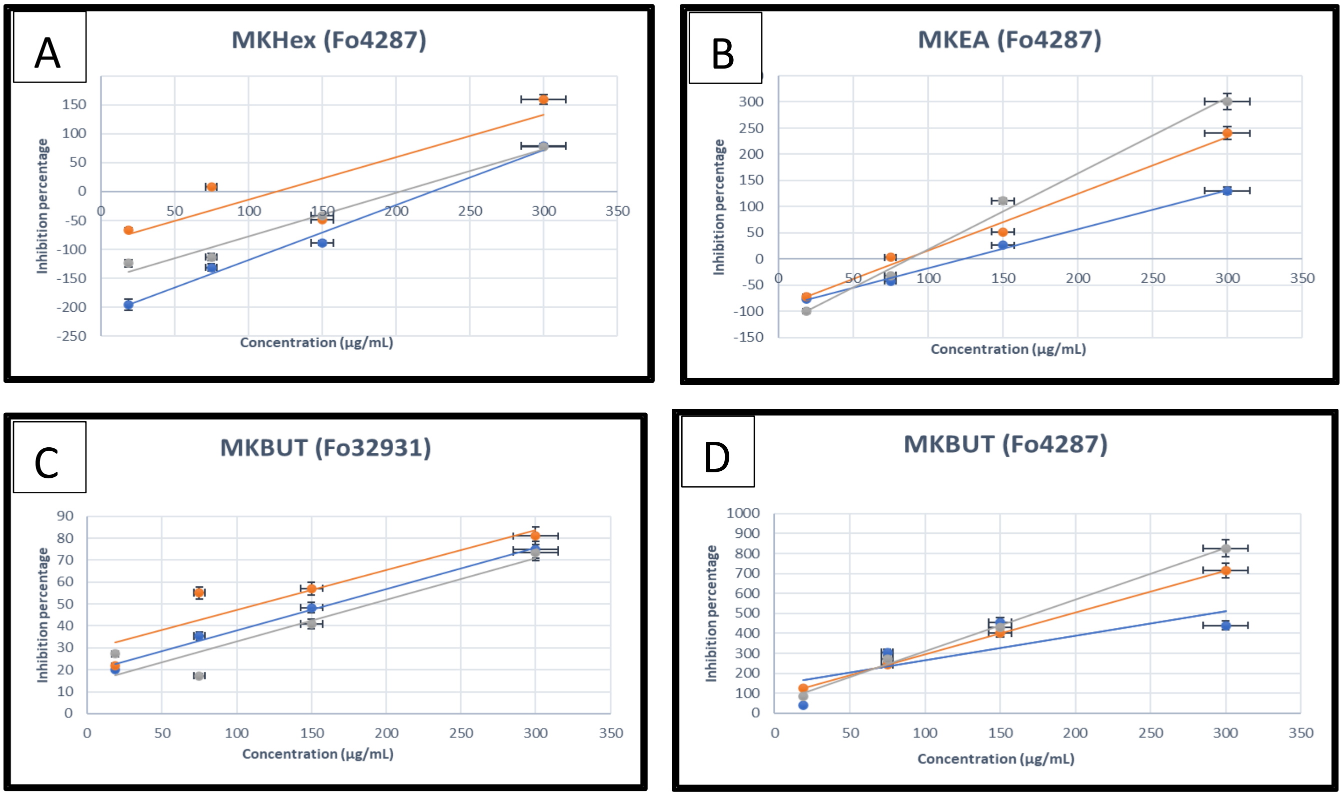

Figure S3: MIC of hexane fraction against *Fo4287* (A), ethyl acetate fraction against *Fo4287* (B), n-butanol fraction against *Fo32931* (C) and against *Fo4287* (D).

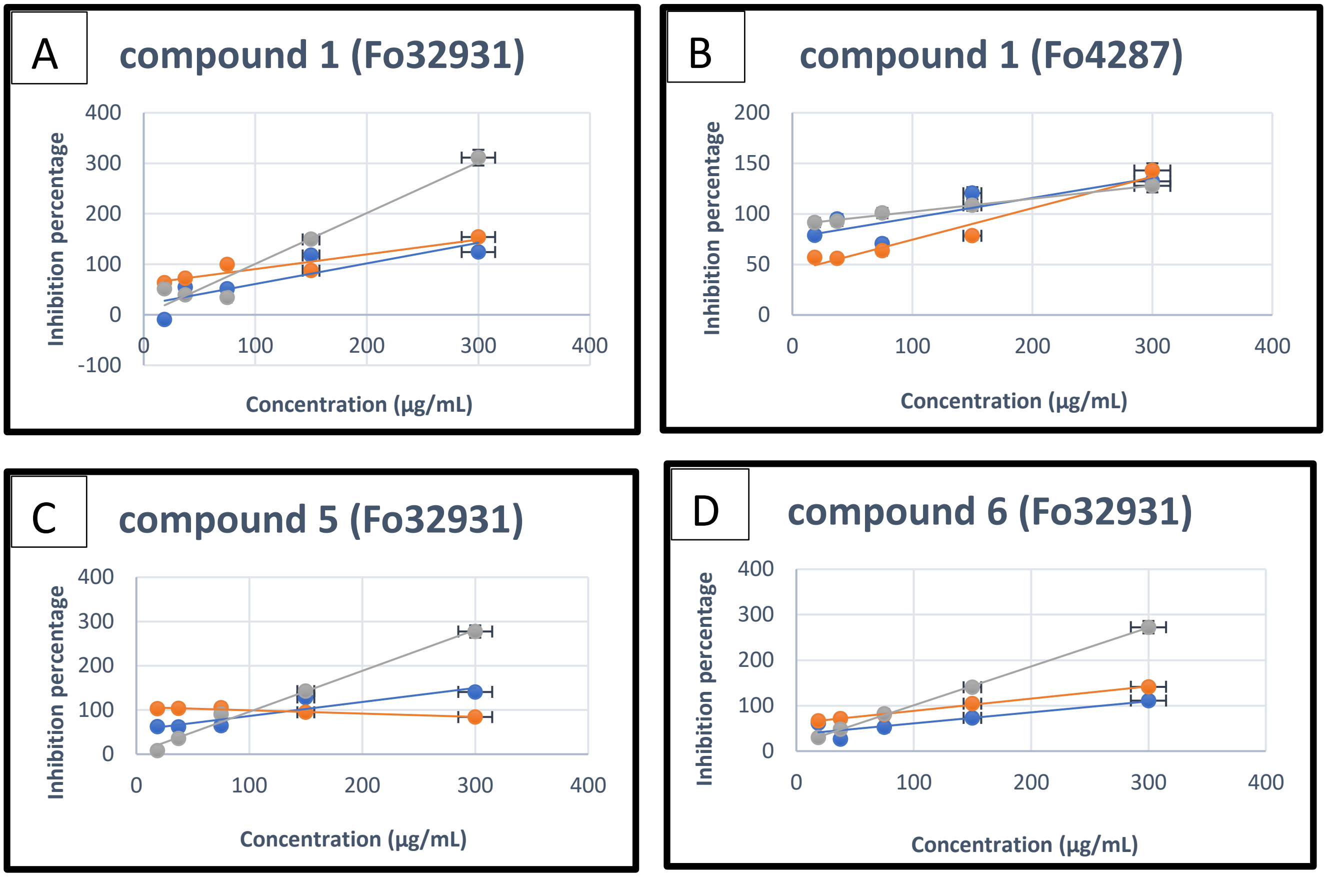

Figure S4: MIC of the most active natural compounds: stilbene-coumarin **1** (A) and (B); ellagic acid derivatives **5** and **6** (C) and (D) against *Fo32931* and *Fo4287*

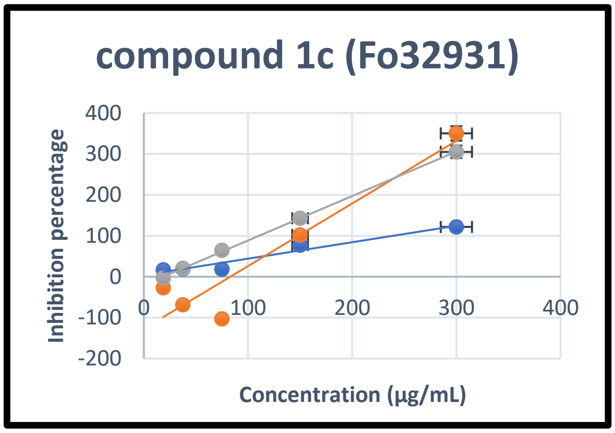

Figure S5: MIC of the most active semisynthetic compound **1c** against *Fo32931*

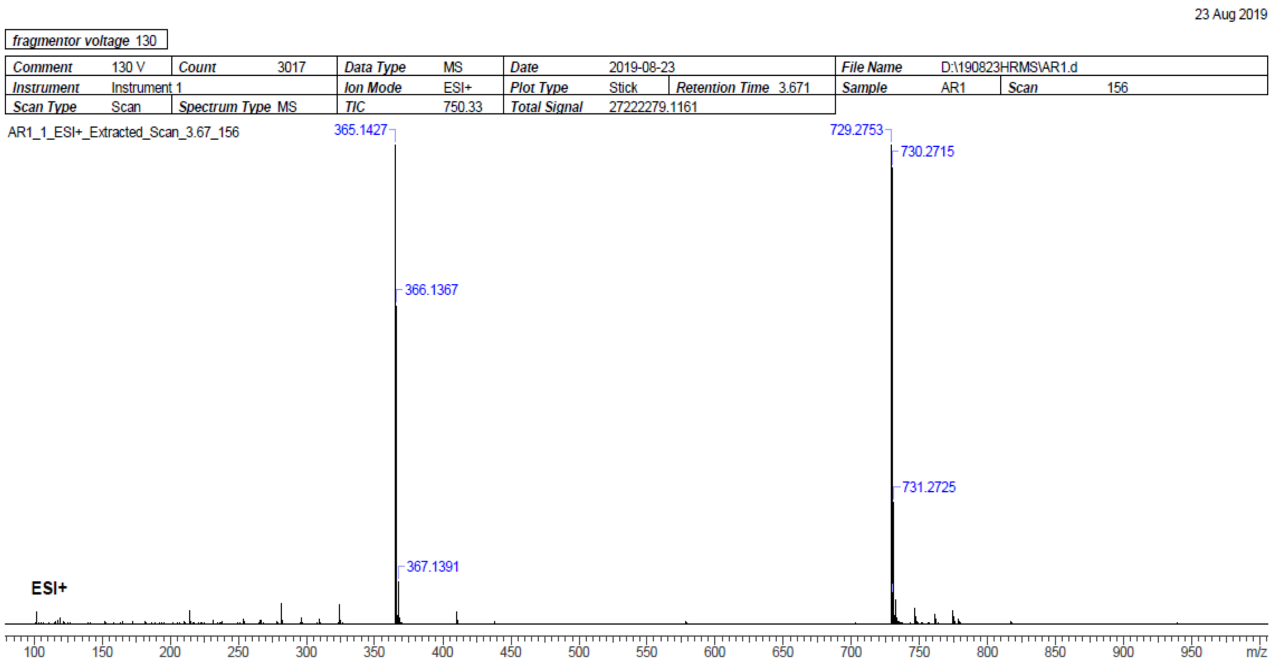

Expected Mass (m/z)	Observed Mass (*m/z*)	Error (ppm)
365.1389	365.1427	10.407

Figure S6: HRESIMS spectrum of compound **1a**

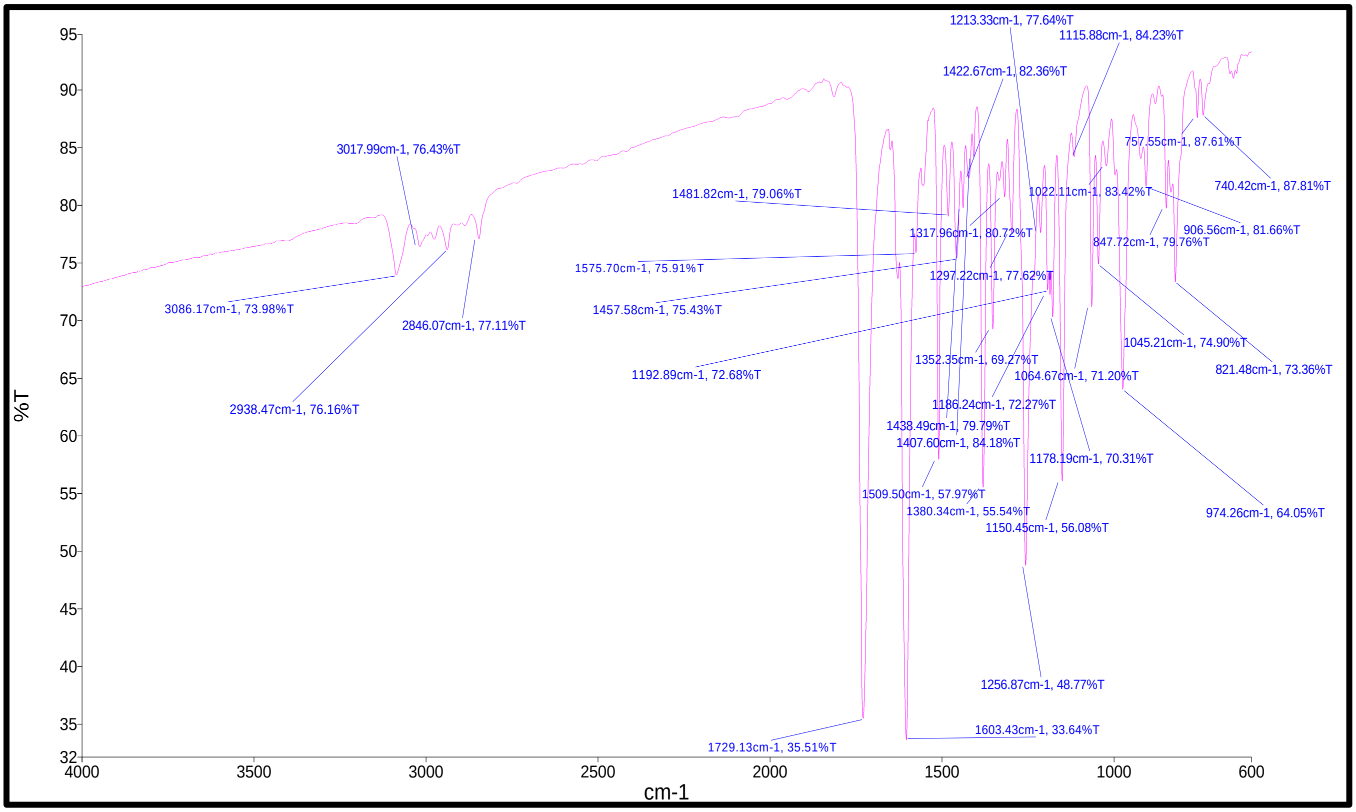

Figure S7: IR spectrum of compound **1a**

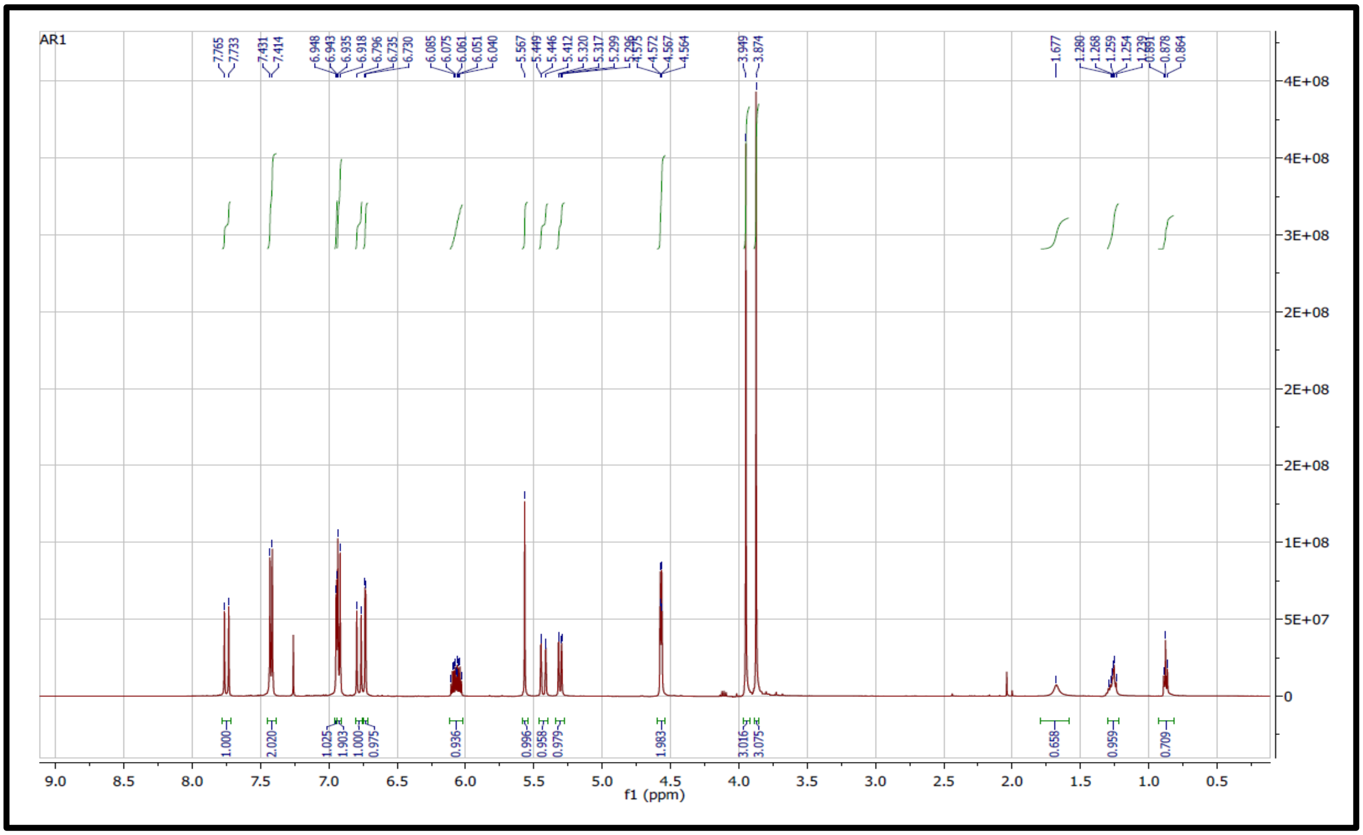

Figure S8: ^{1}H NMR ($CDCl_3$, 500 MHz) spectrum of compound **1a**

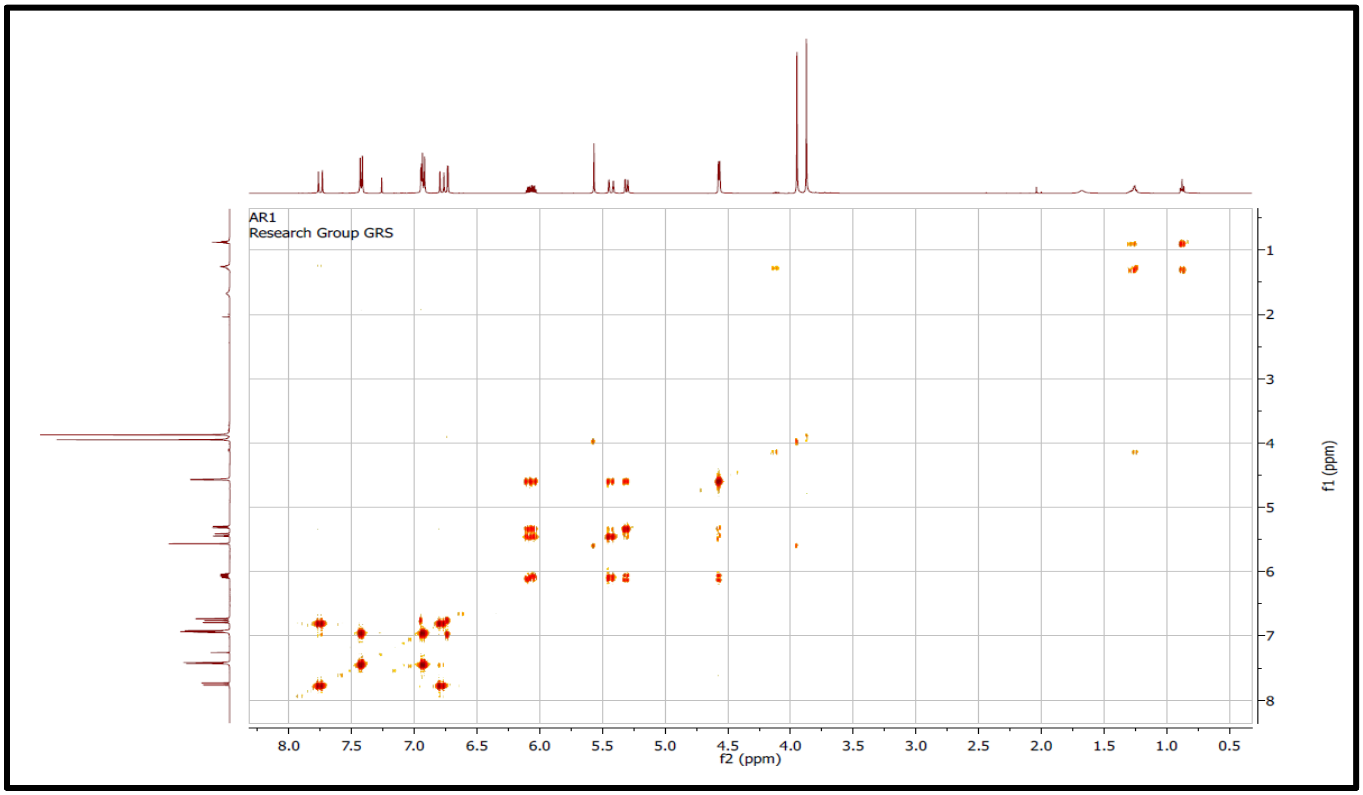

Figure S9: COSY spectrum of compound **1a**

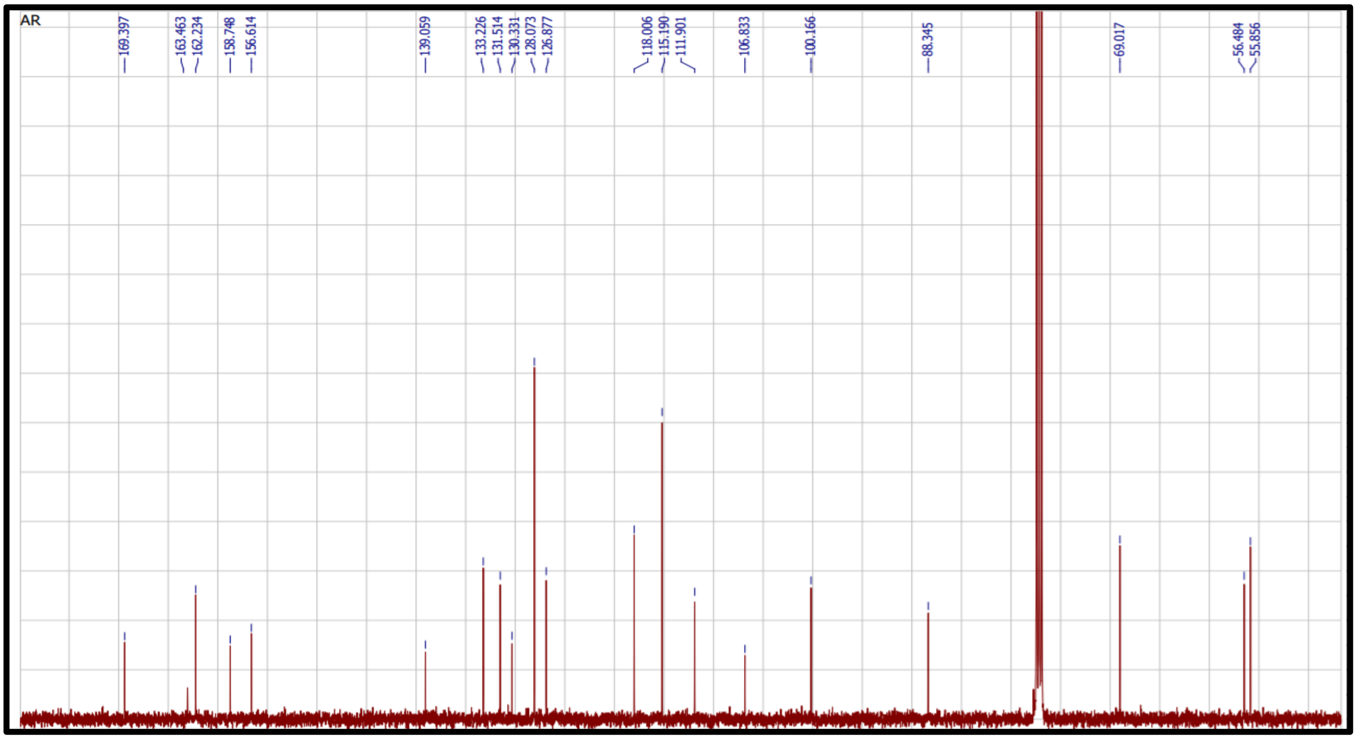

Figure S10: ^{13}C NMR spectrum ($CDCl_3$, 125 MHz) of compound **1a**

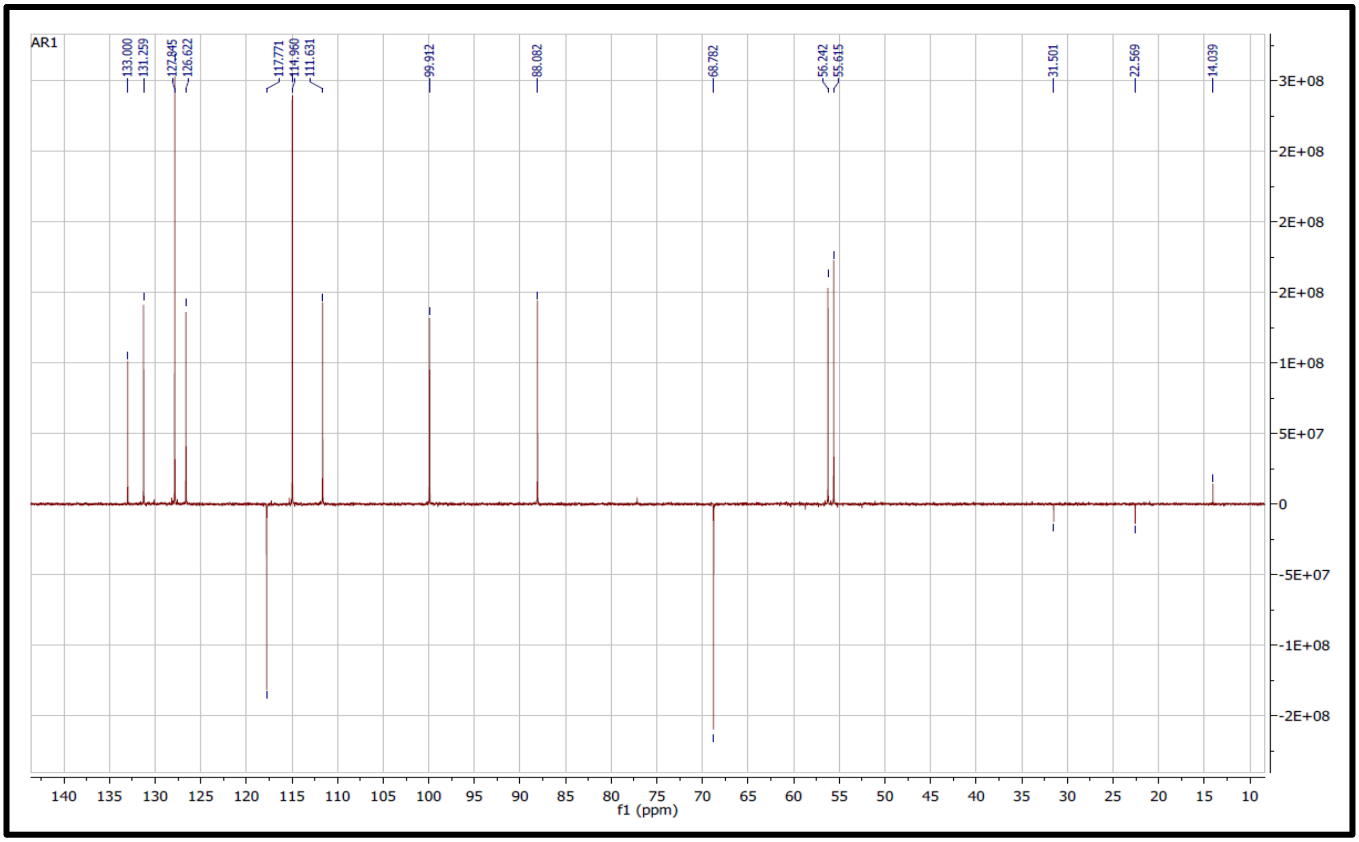

Figure S11: DEPT 135 spectrum of compound **1a**

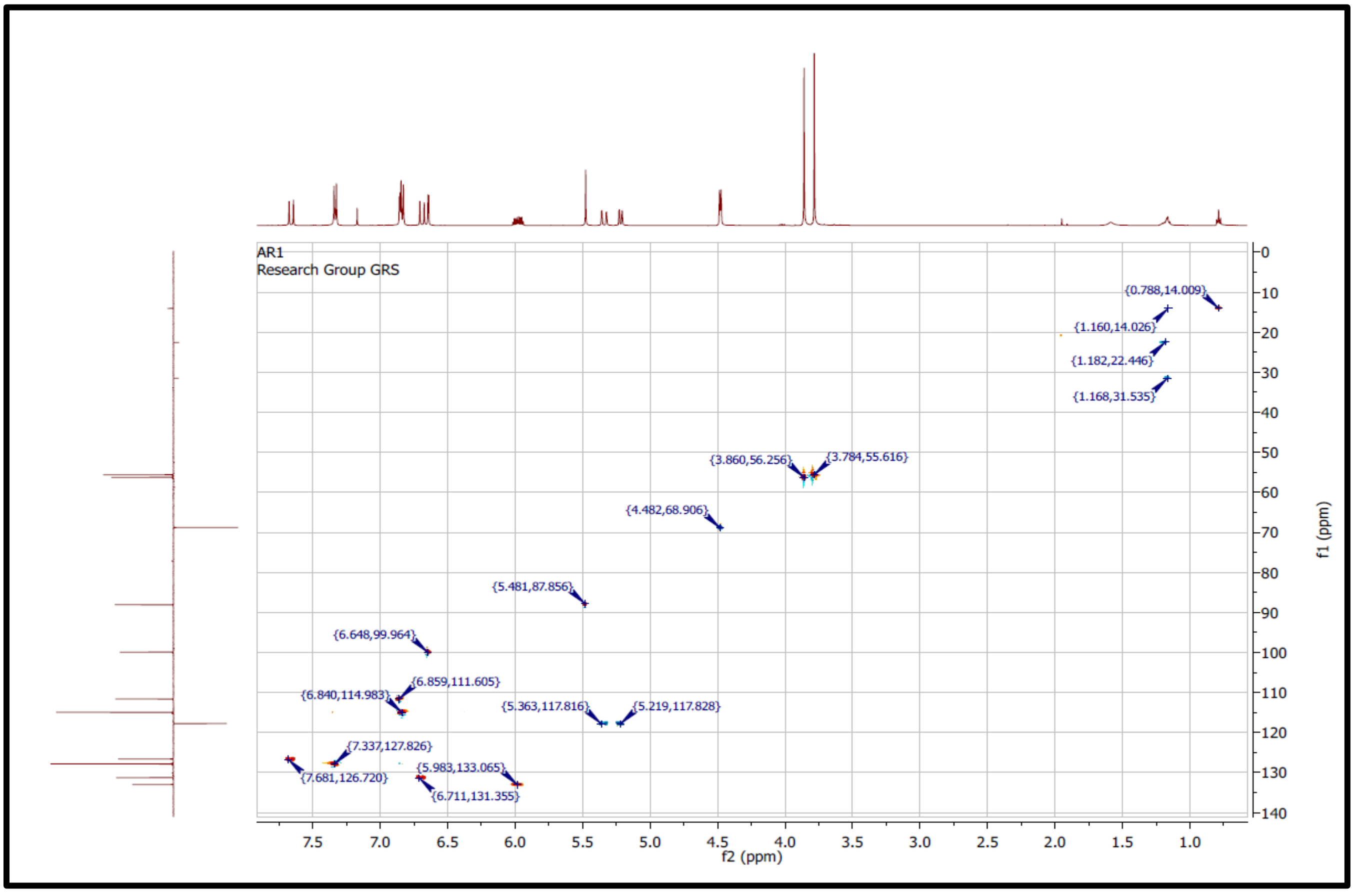

Figure S12: HSQC spectrum of compound **1a**

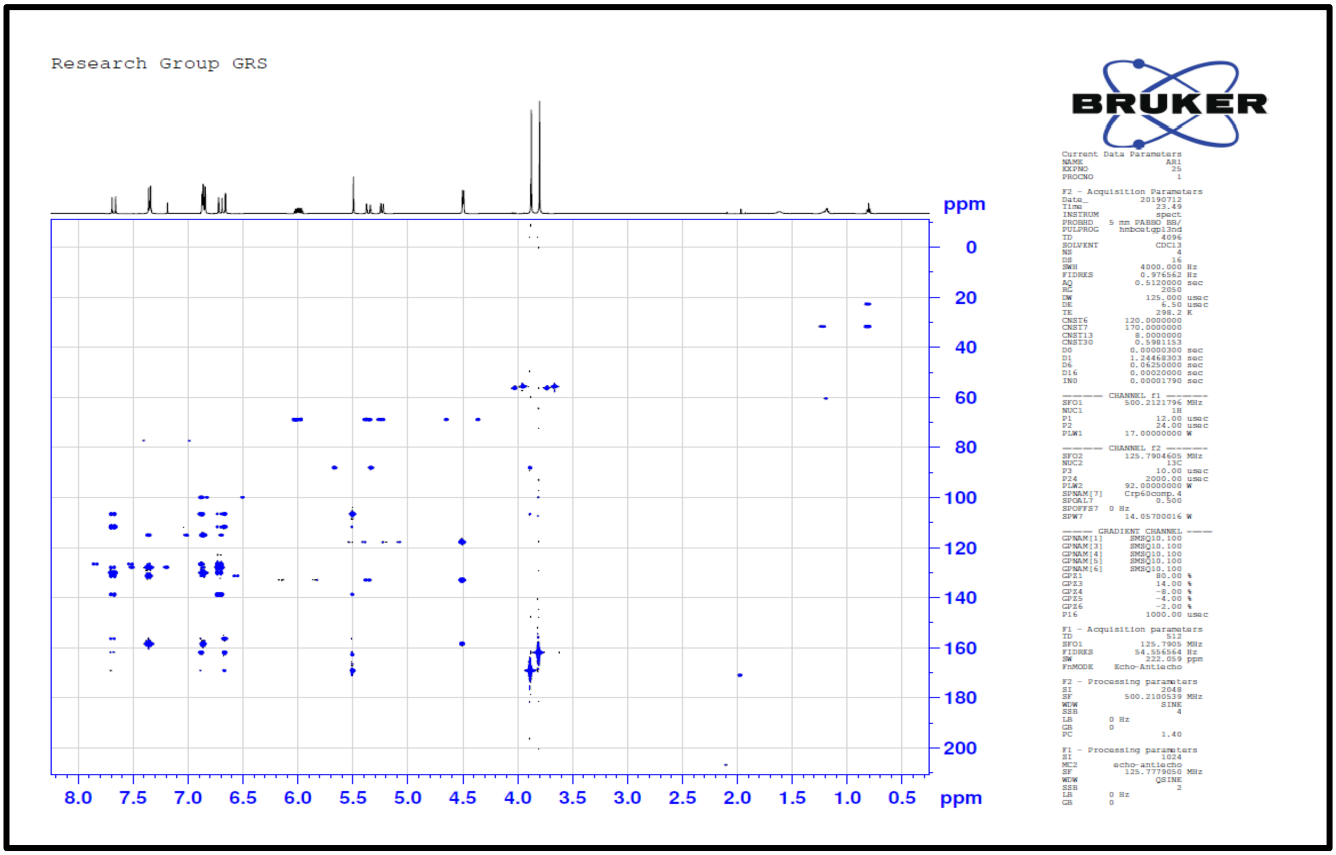

Figure S13: HMBC spectrum of compound **1a**

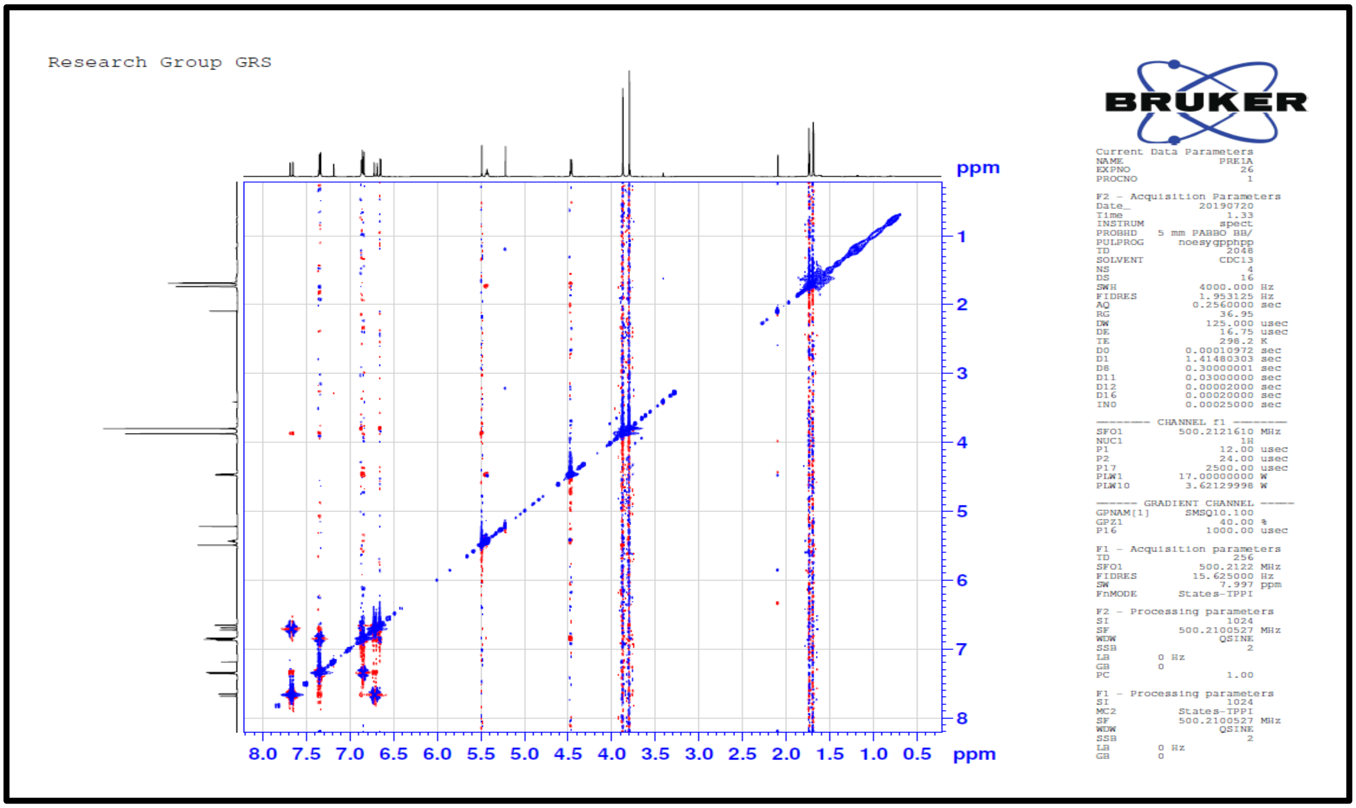

Figure S14: ROESY spectrum of compound **1a**

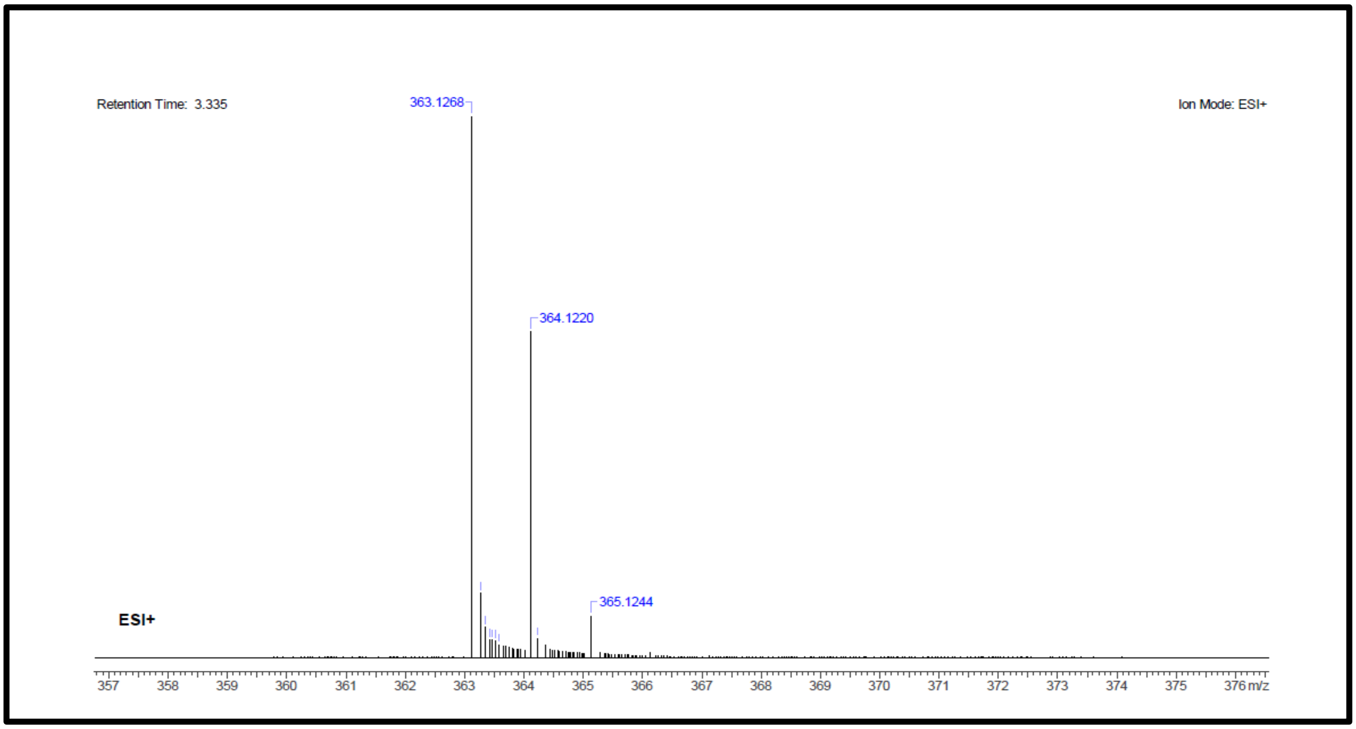

Figure S15: HRESIMS spectrum of compound **1b**

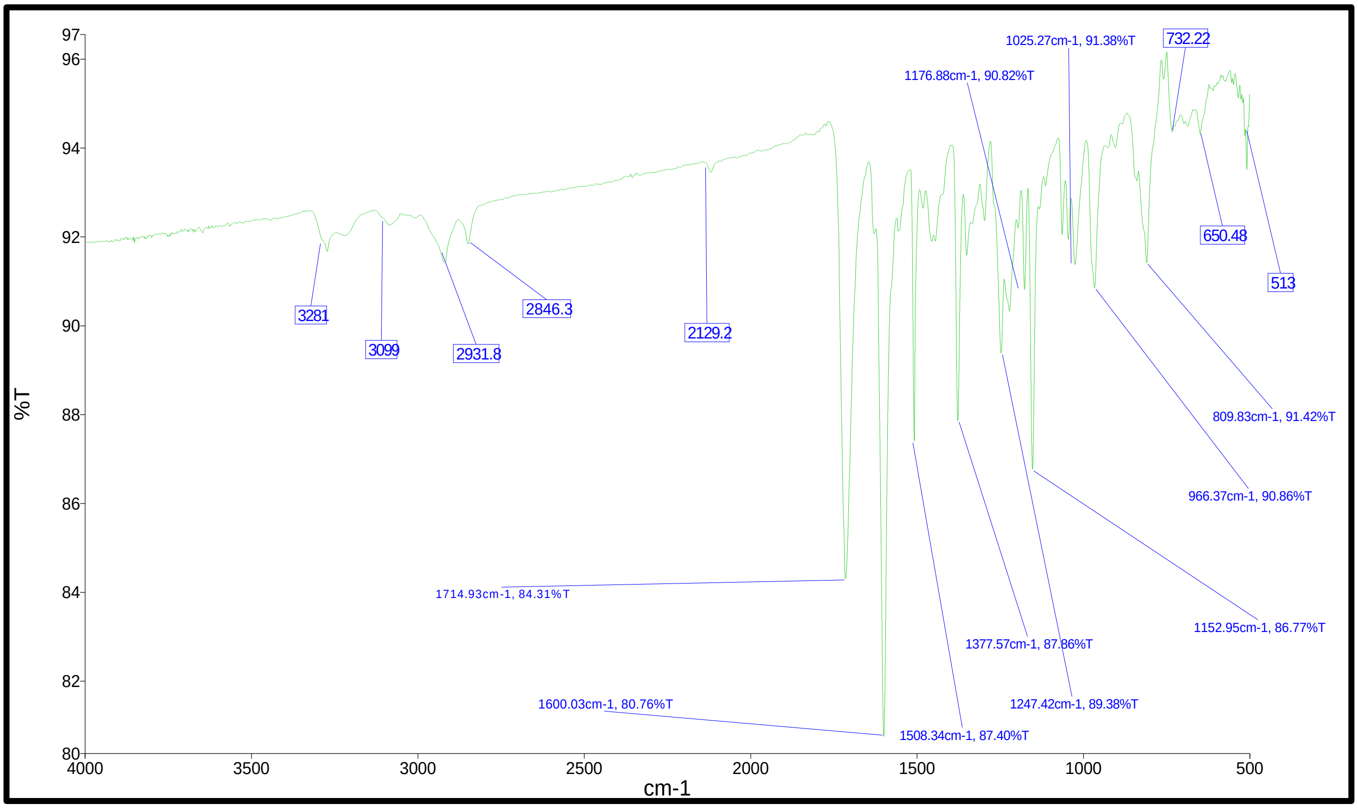

Figure S16: IR spectrum of compound **1b**

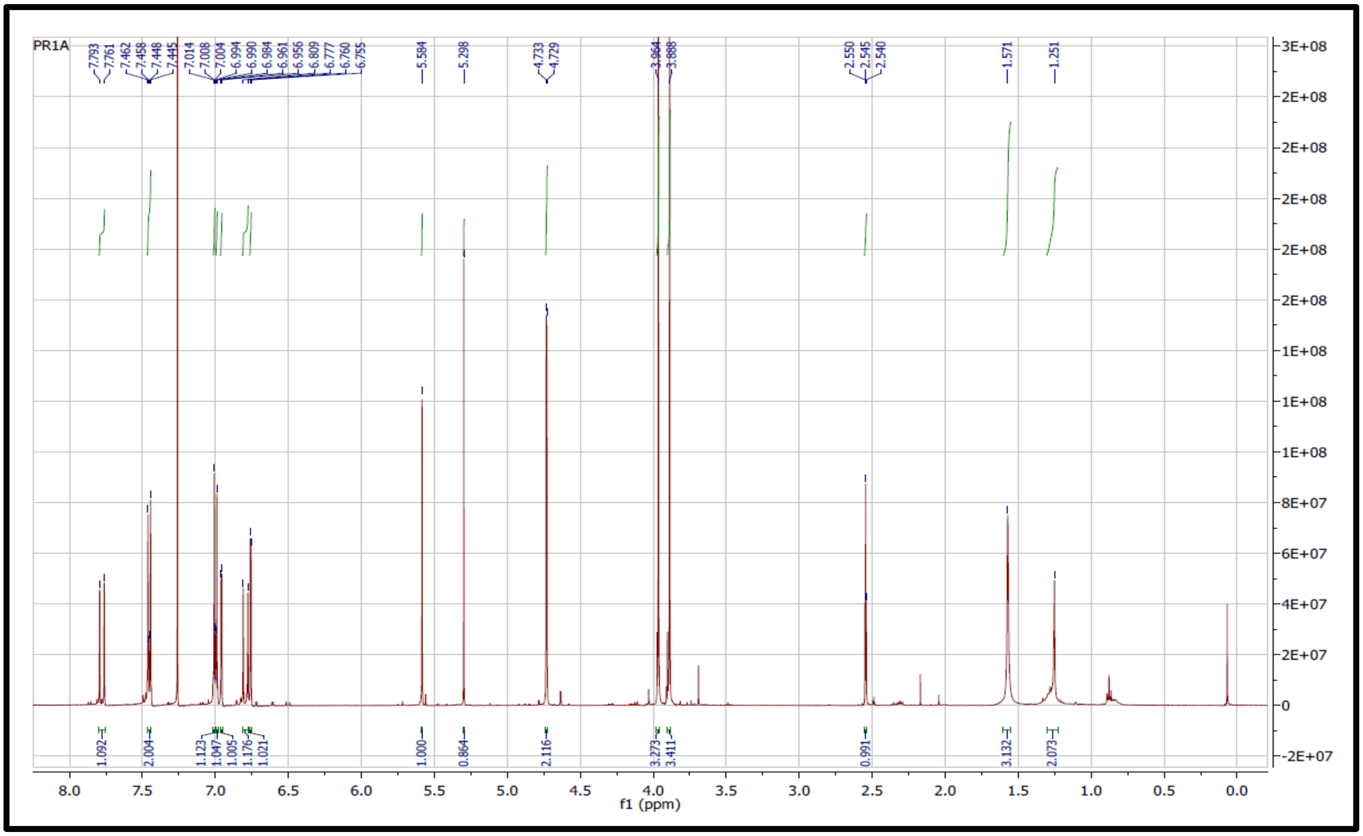

Figure S17: ^{1}H NMR spectrum ($CDCl_3$, 500 MHz) of compound **1b**

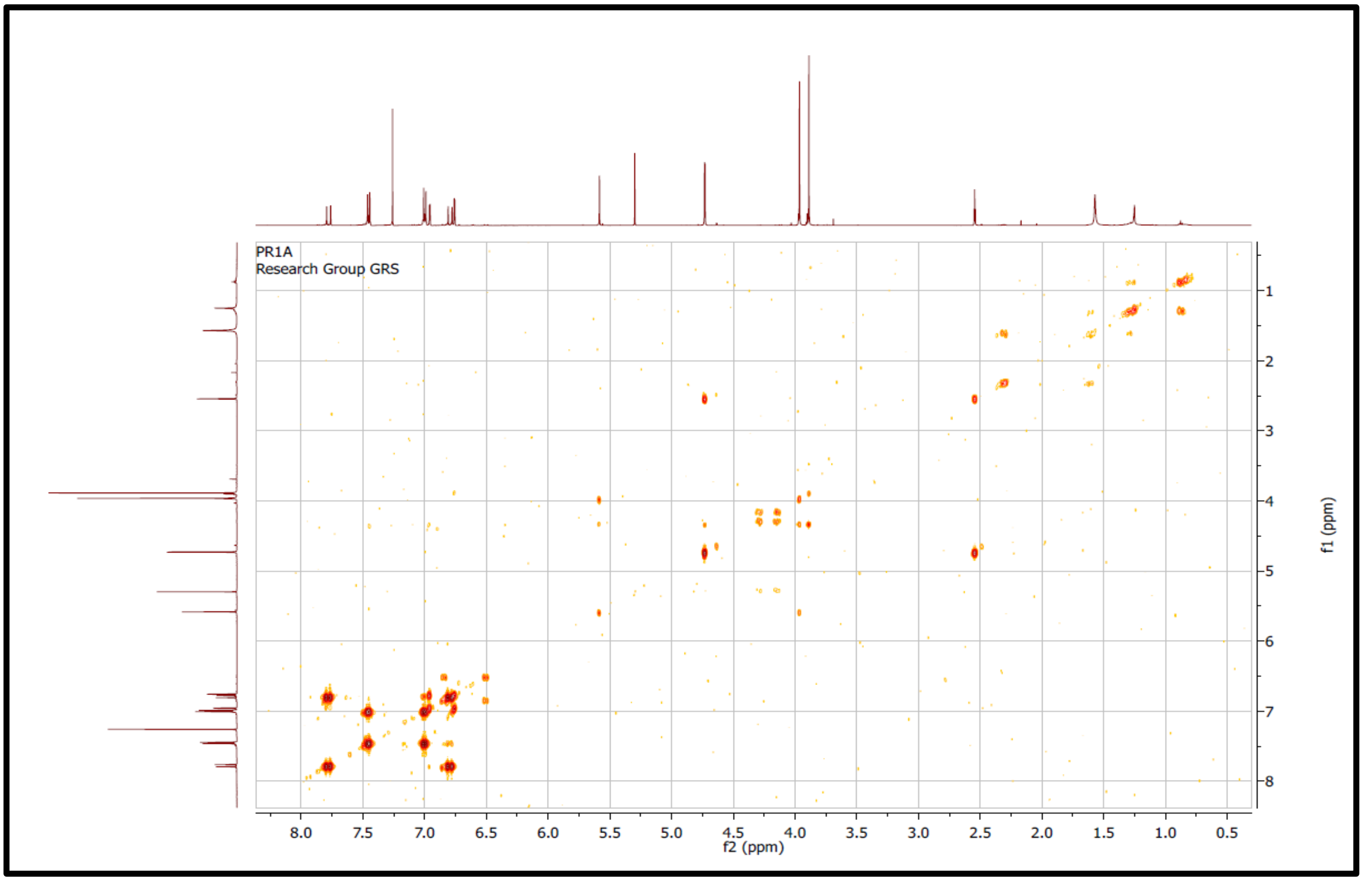

Figure S18: COSY spectrum of compound **1b**

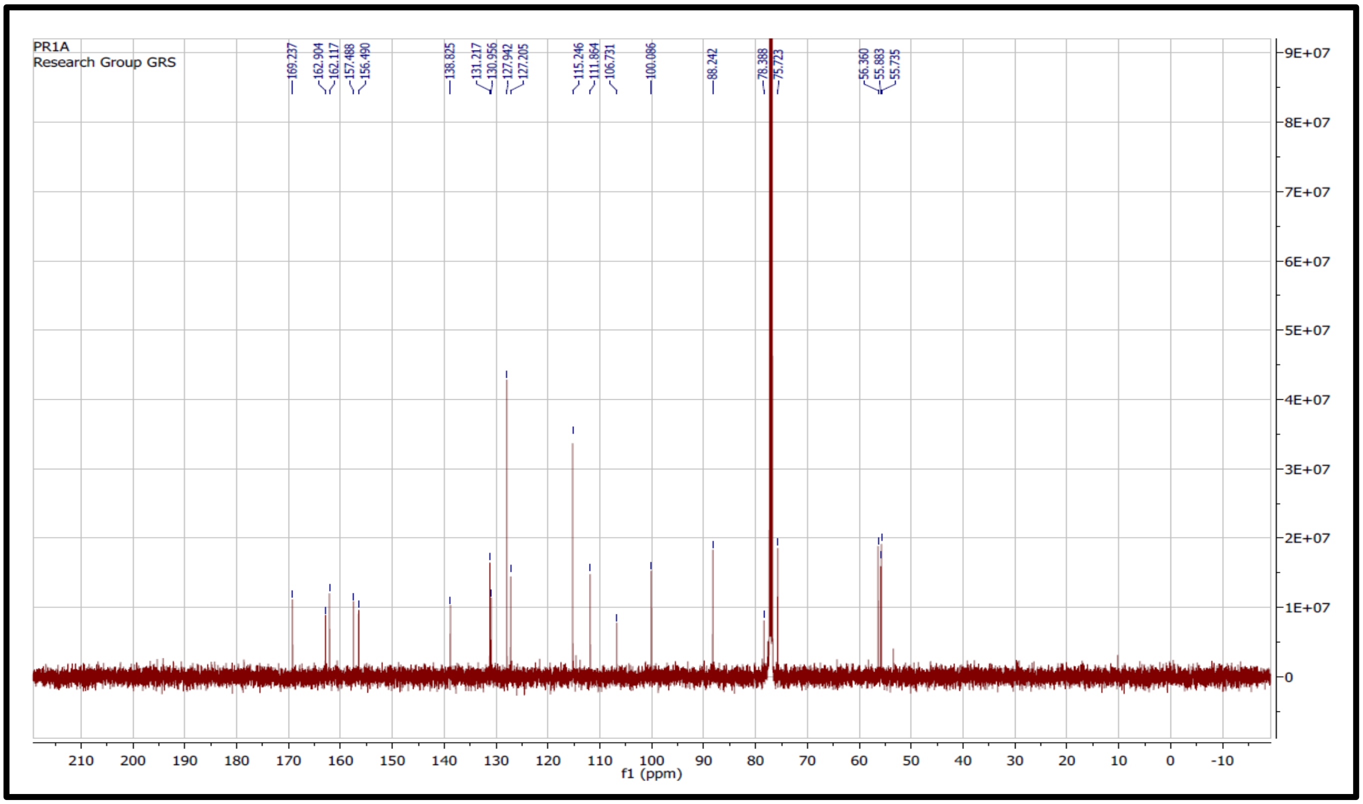

Figure S19: ^{13}C NMR spectrum ($CDCl_3$, 125 MHz) of compound **1b**

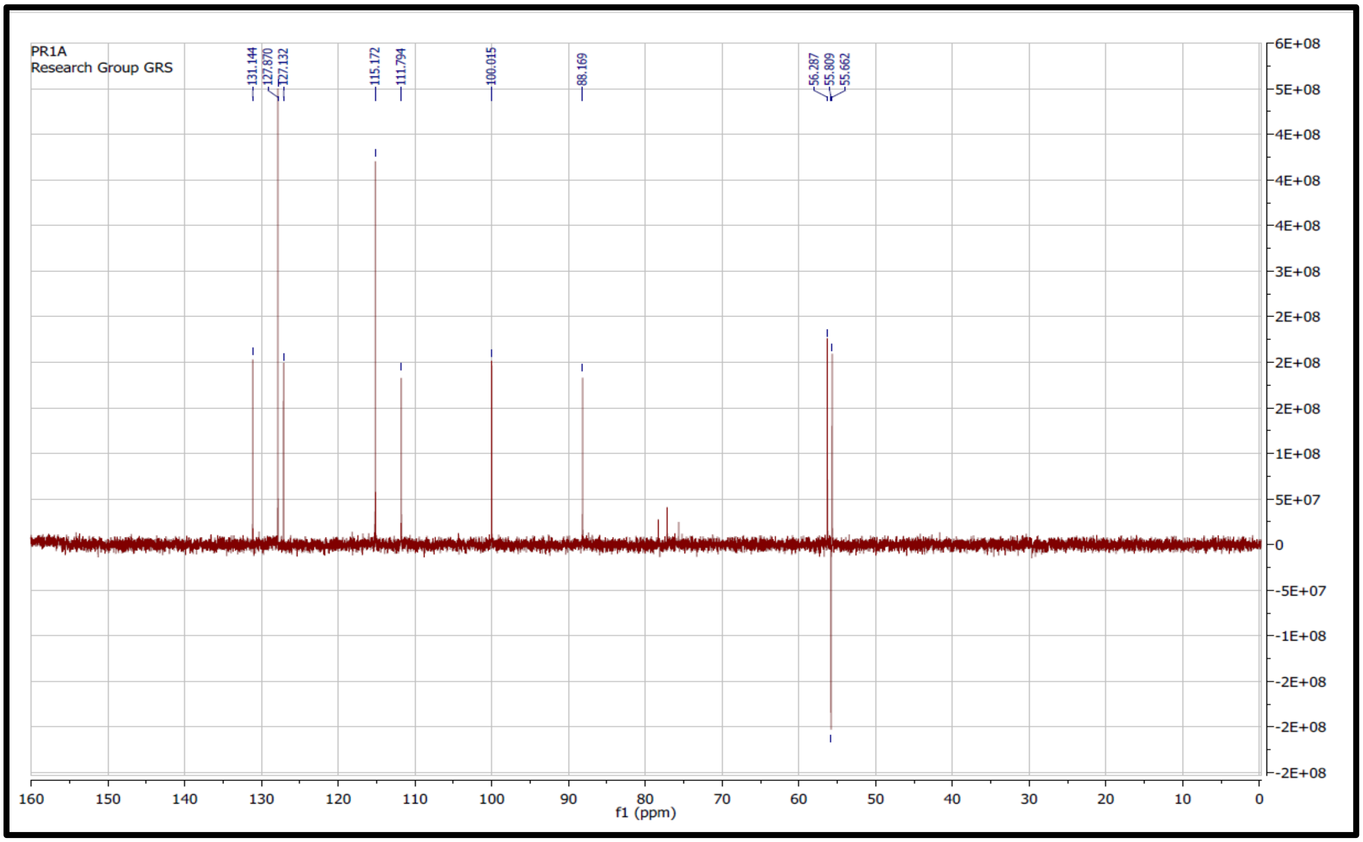

Figure S20: DEPT 135 spectrum of compound **1b**

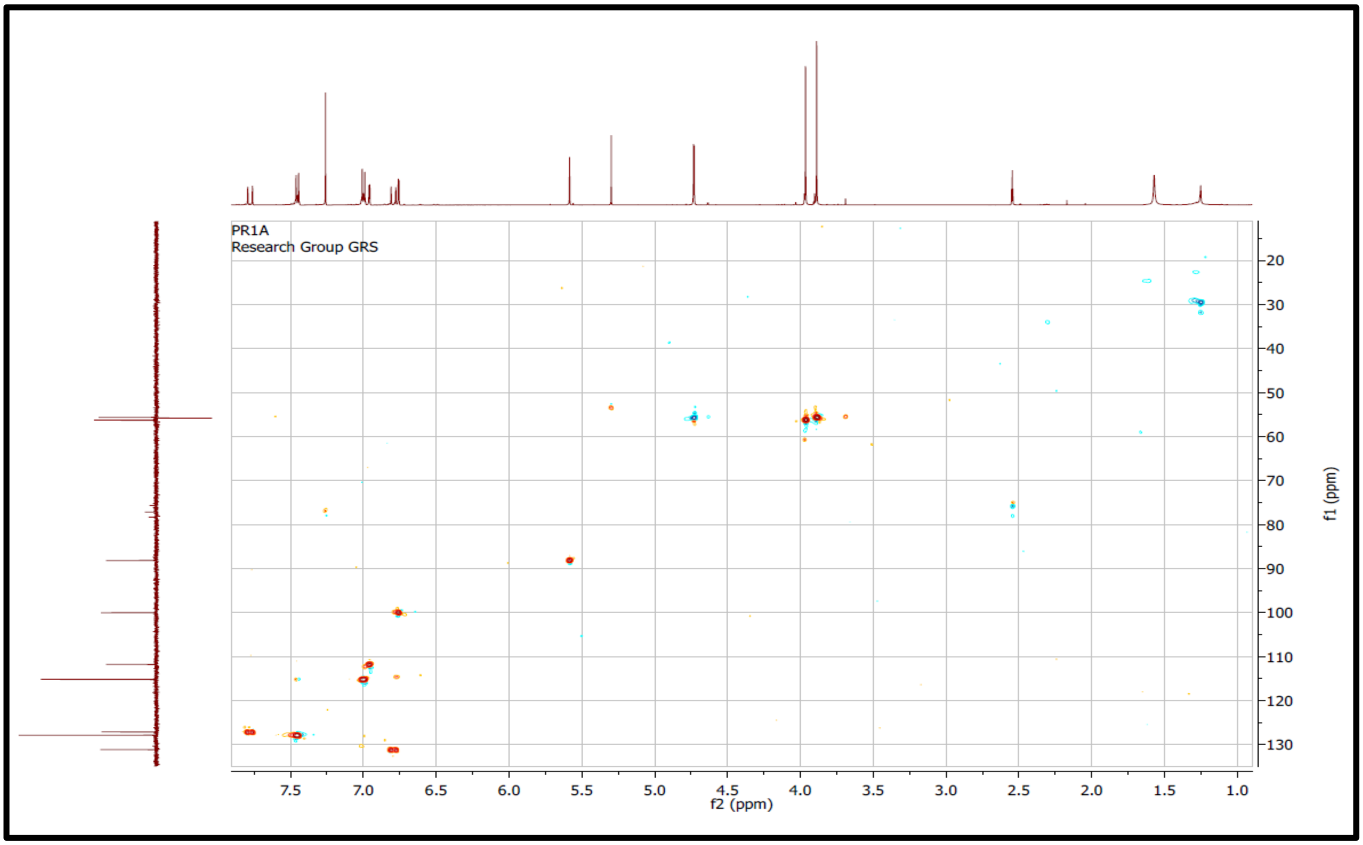

Figure S21: HSQC spectrum of compound **1b**

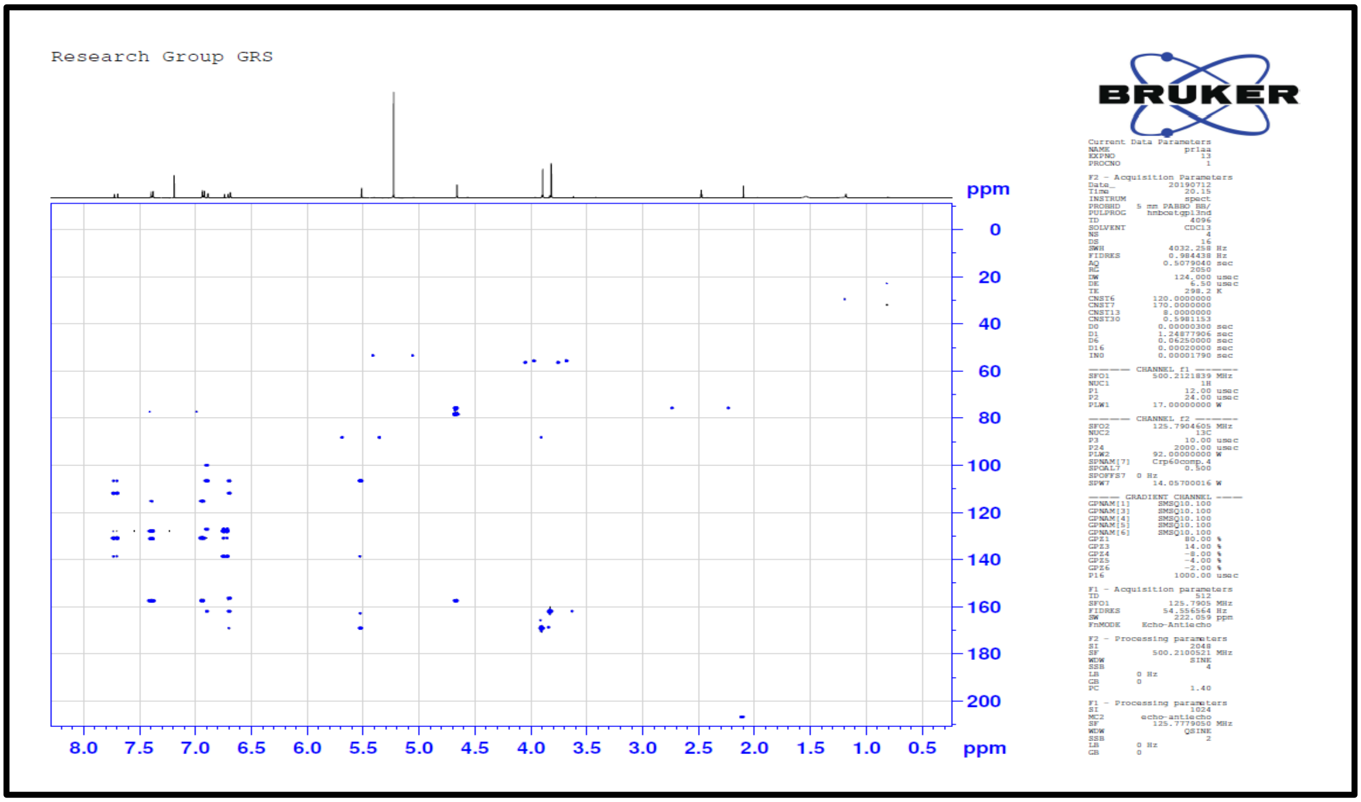

Figure S22: HMBC spectrum of compound **1b**

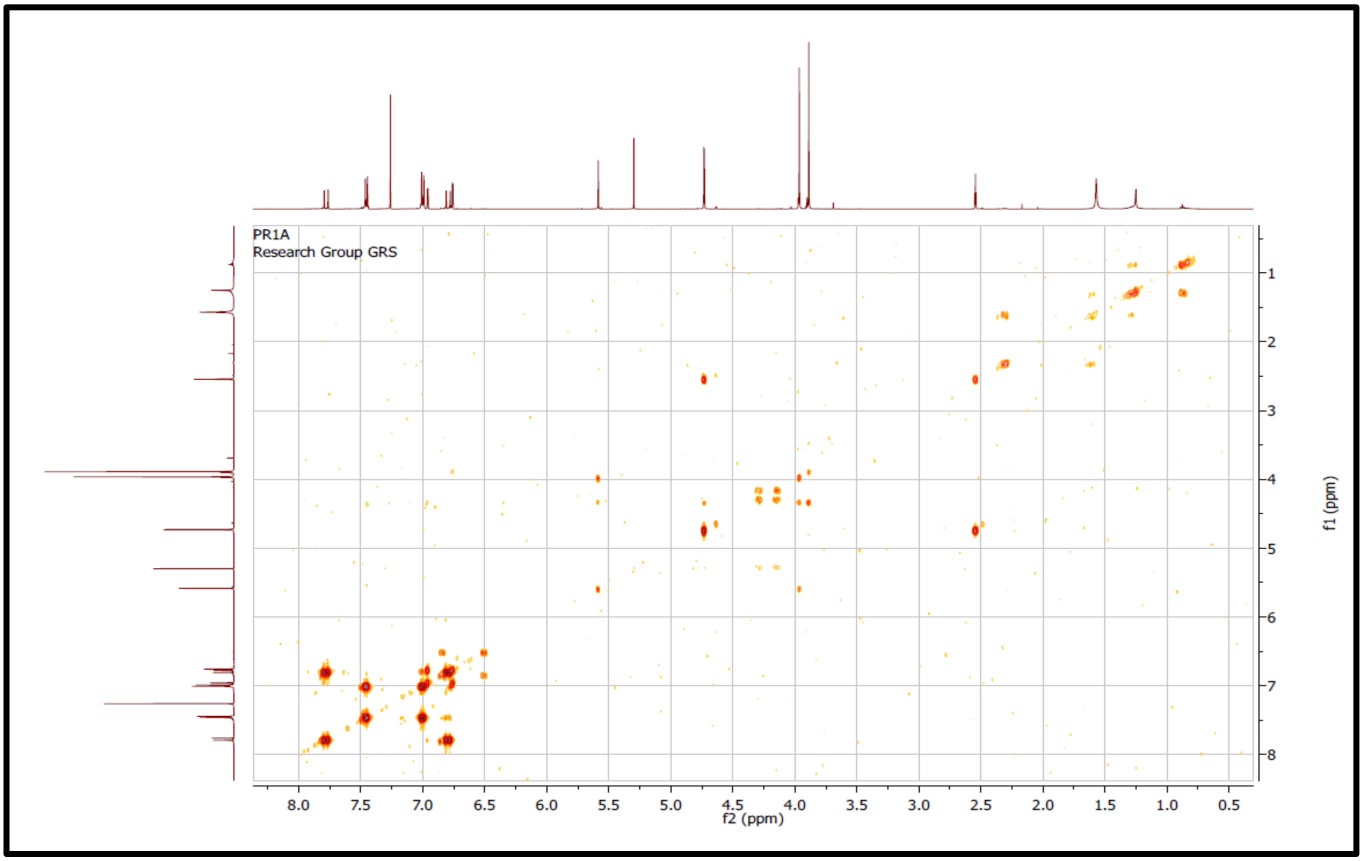

Figure S23: NOESY spectrum of compound **1b**

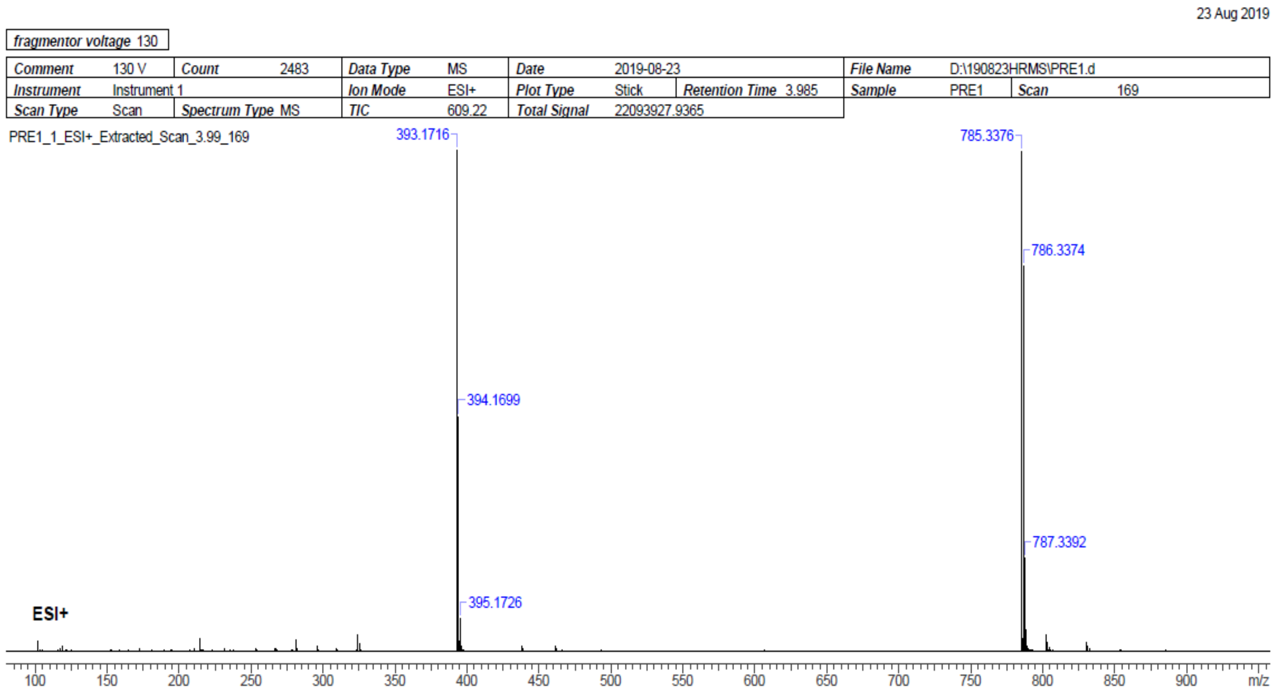

Expected Mass (*m/z*)	Observed Mass (*m/z*)	(Error (ppm)
393.1702	393.1716	3.560799

Figure S24: HRESIMS spectrum of compound **1c**

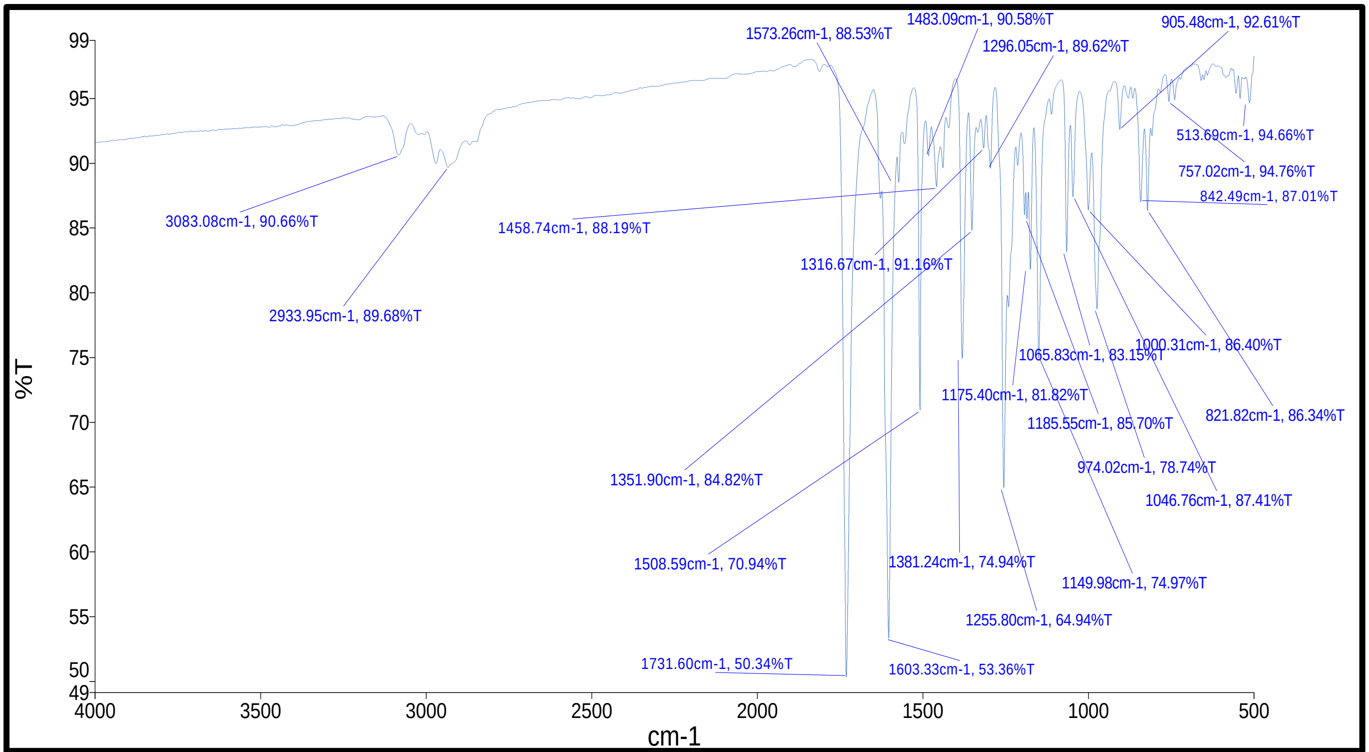

Figure S25: IR spectrum of compound **1c**

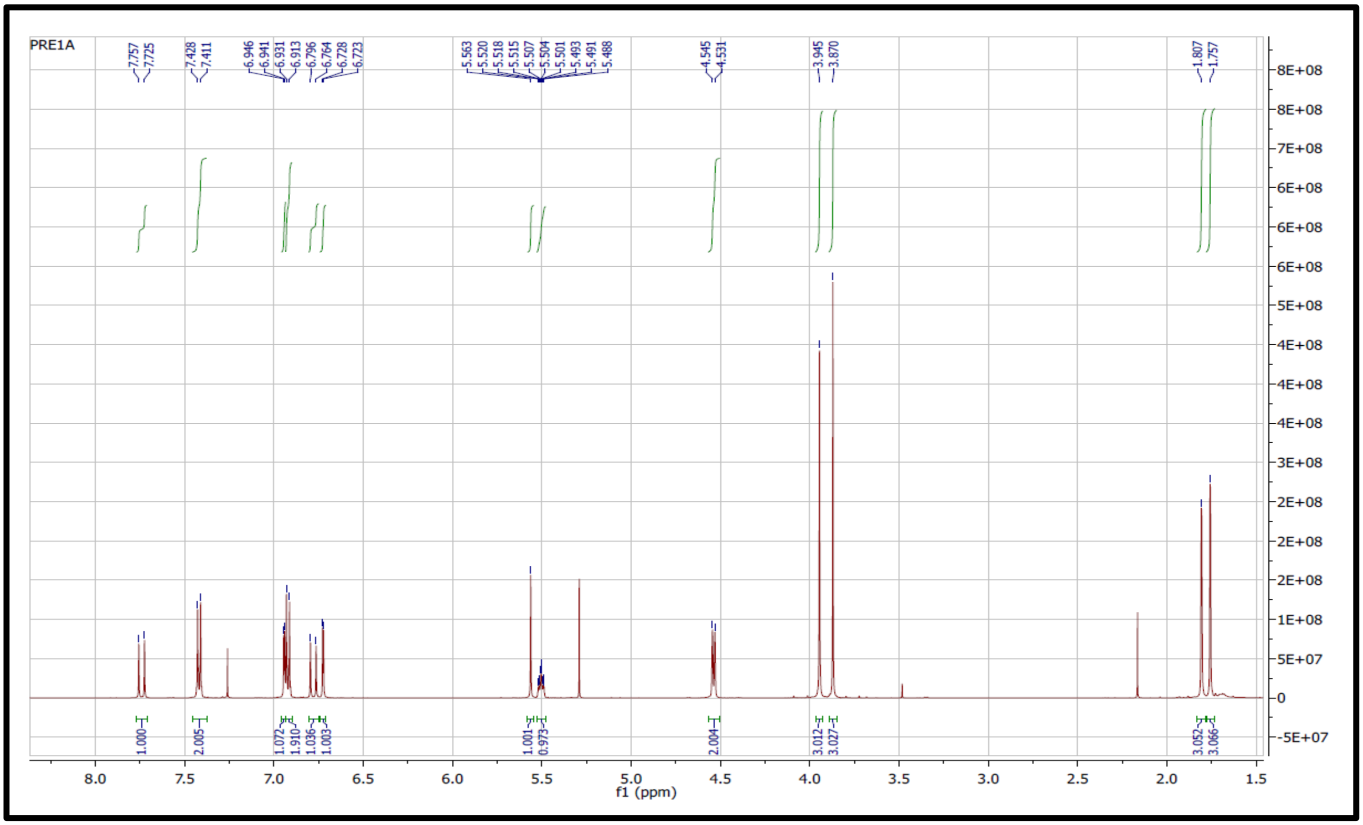

Figure S26: ^{1}H NMR spectrum ($CDCl_3$, 500 MHz) of compound **1c**

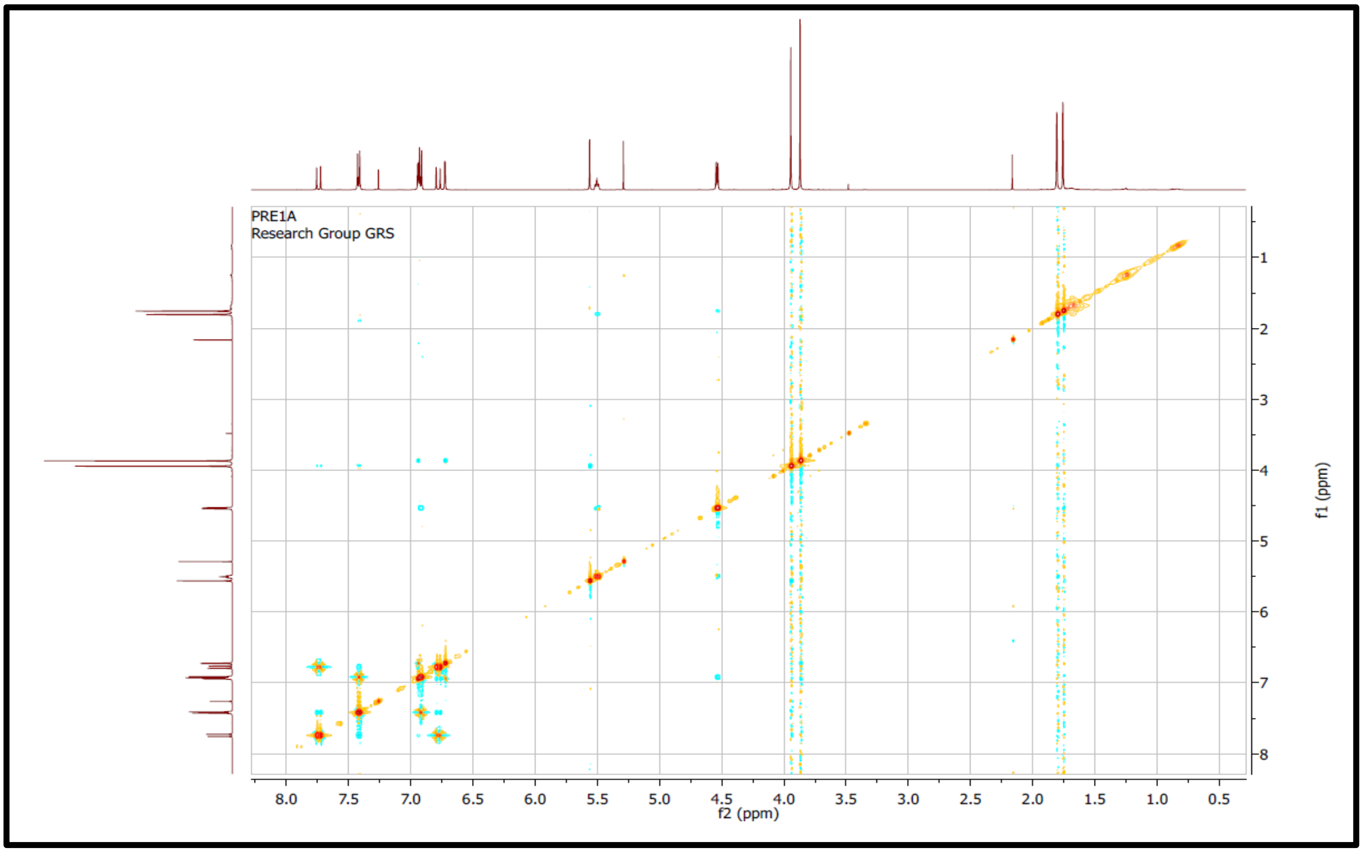

Figure S27: COSY spectrum of compound **1c**

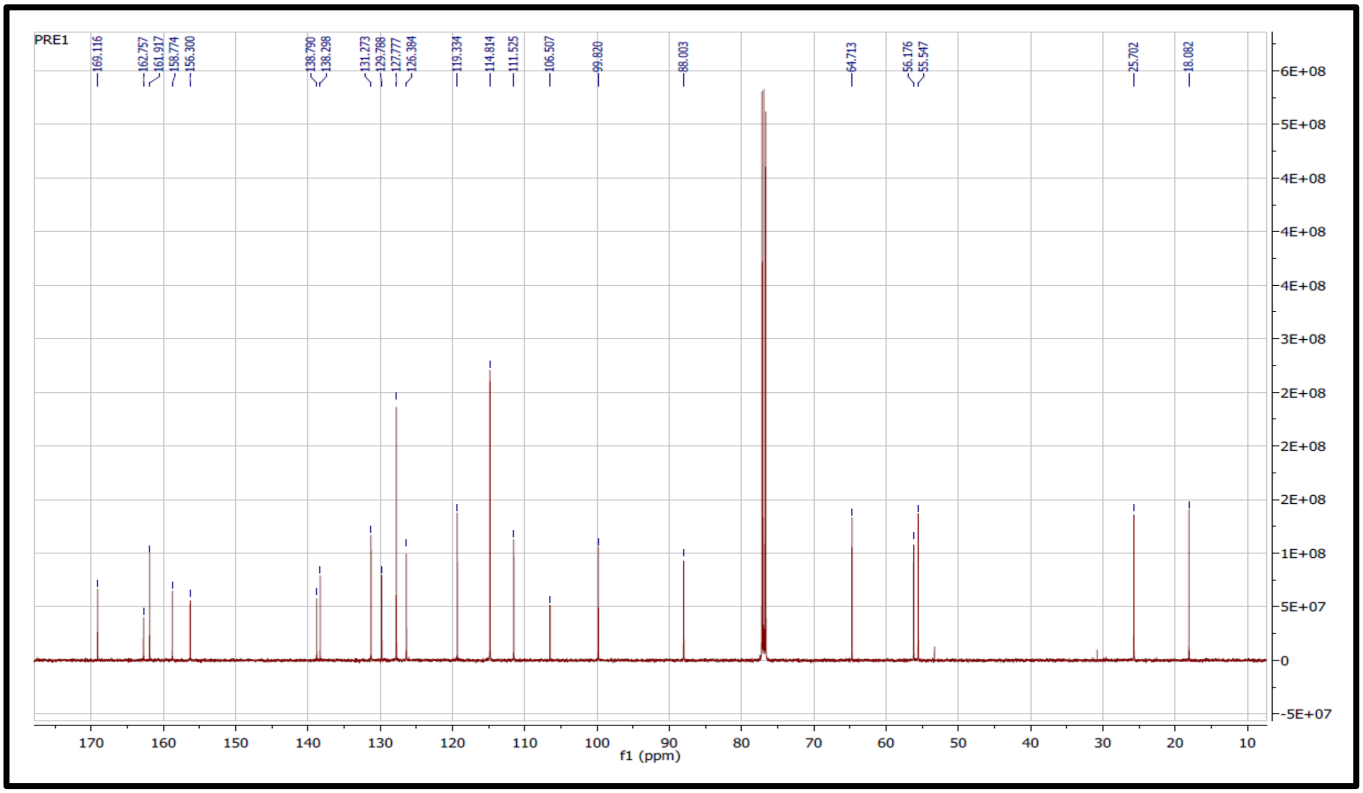

Figure S28: ^{13}C NMR spectrum ($CDCl_3$, 125 MHz) of compound **1c**

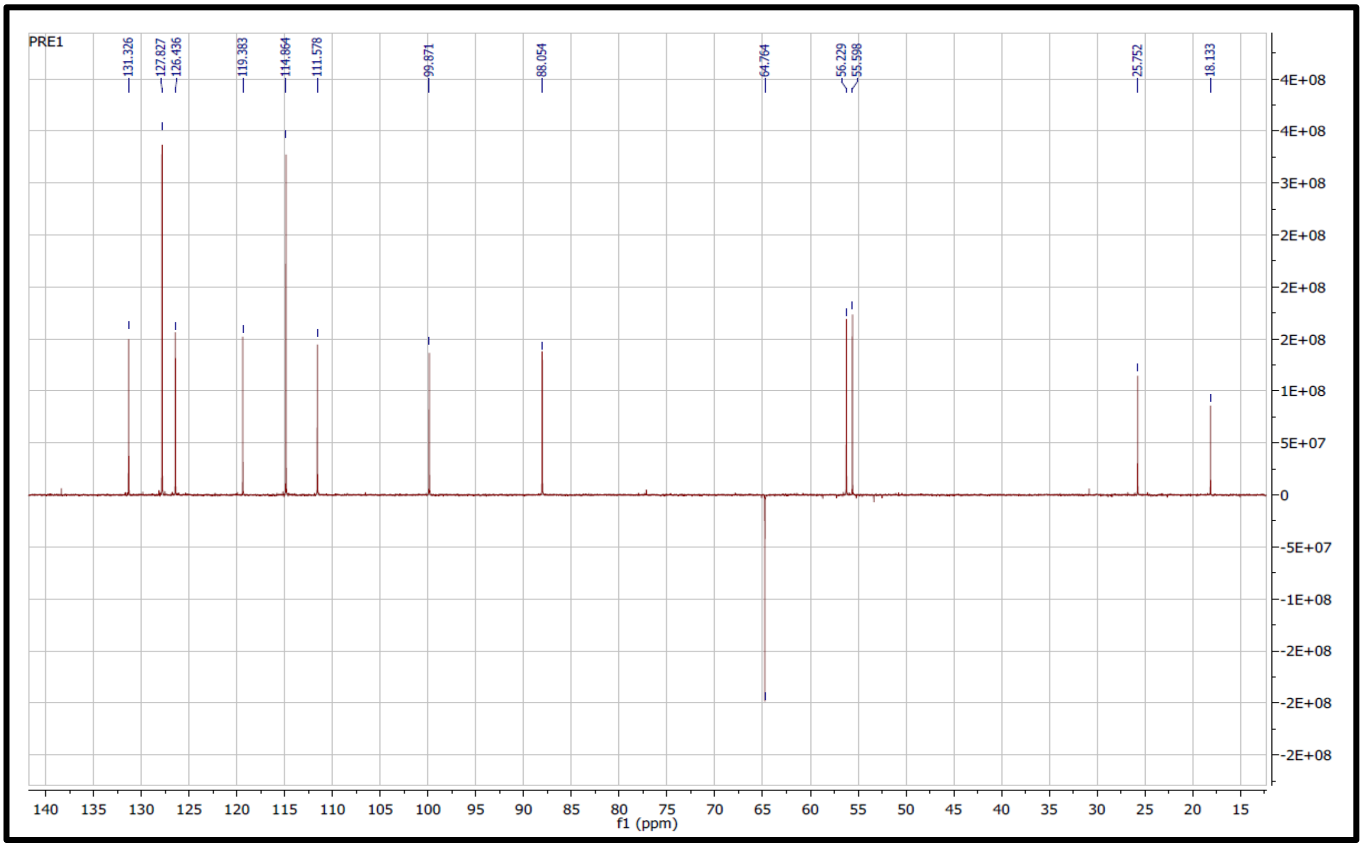

Figure S29: DEPT 135 spectrum of compound **1c**

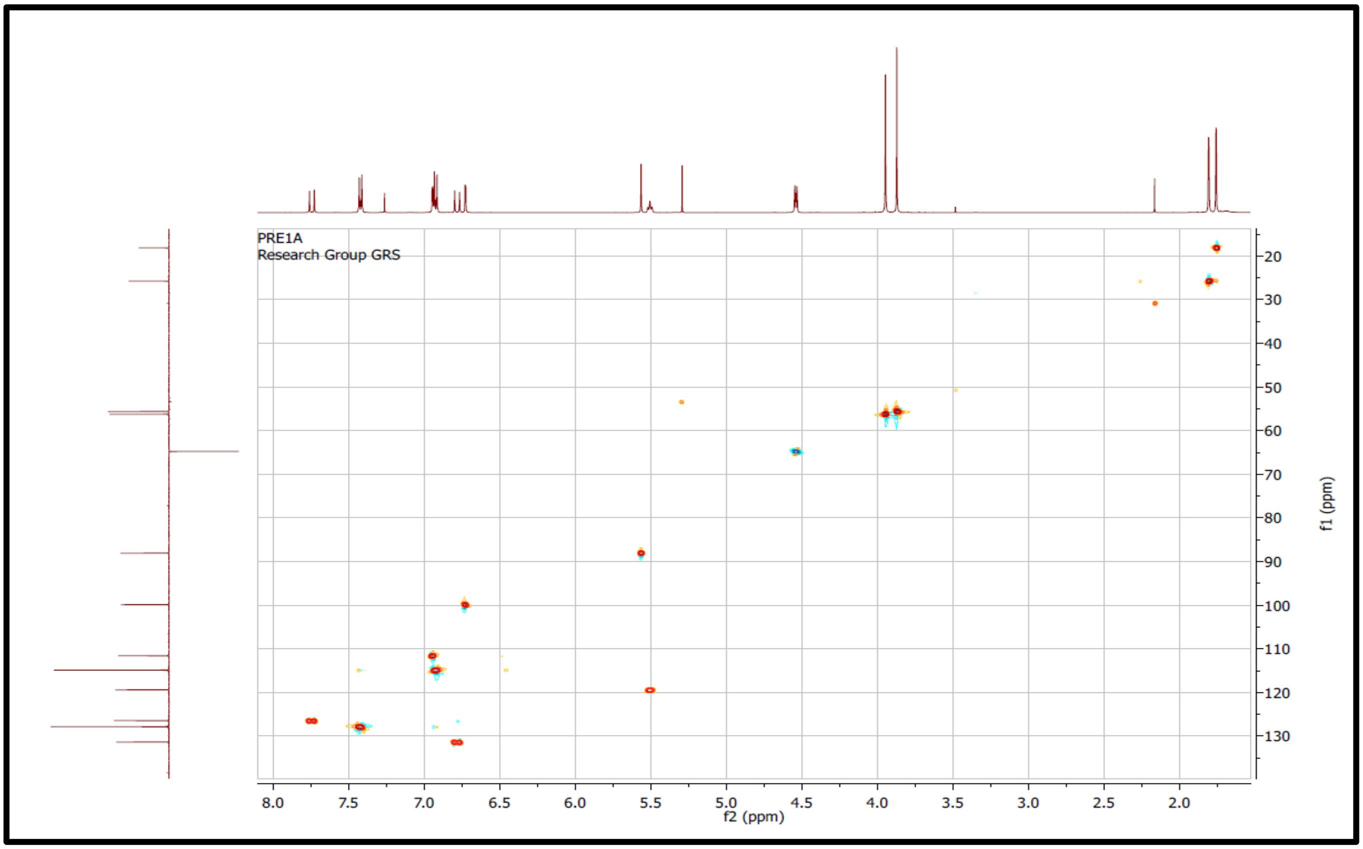

Figure S30: HSQC spectrum of compound **1c**

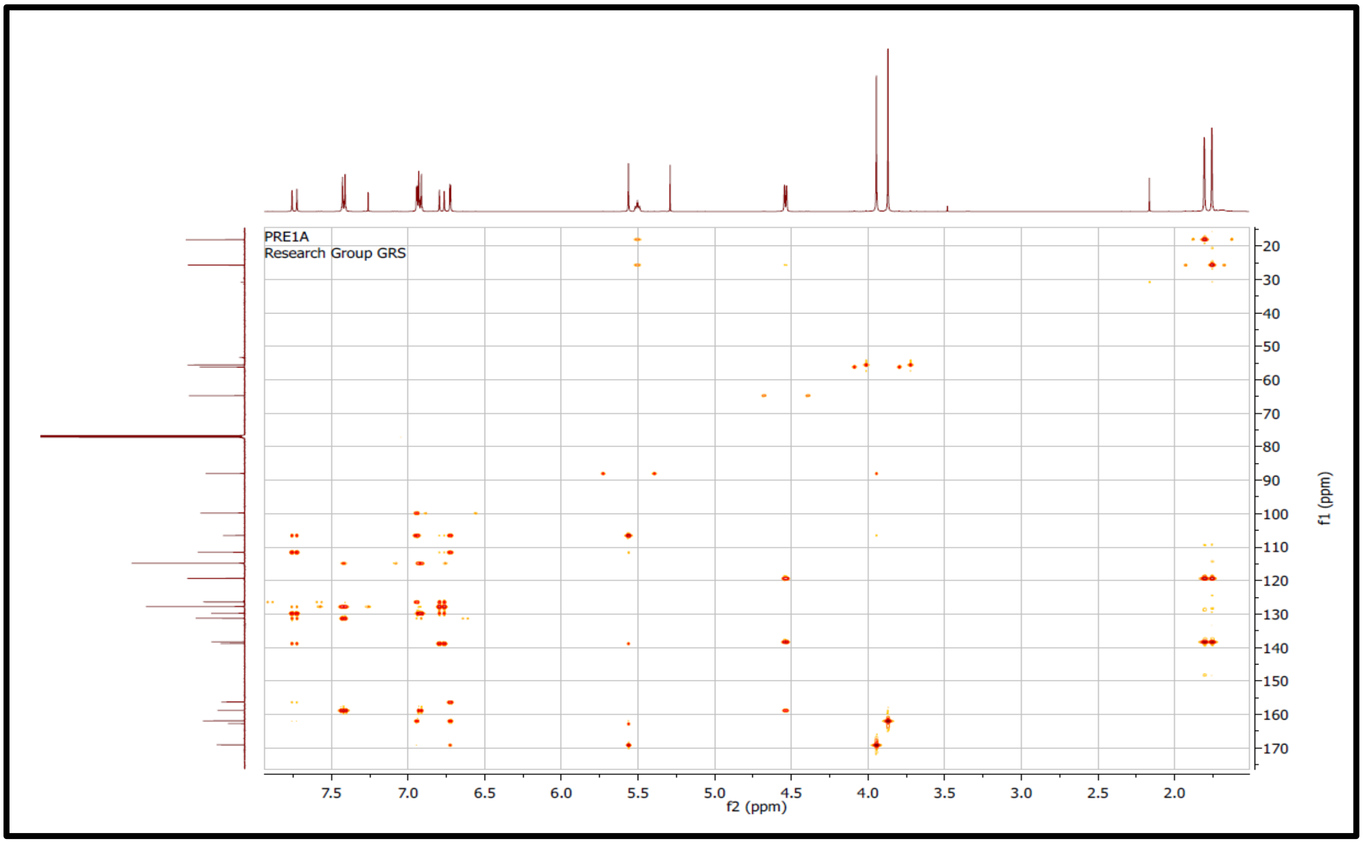

Figure S31: HMBC spectrum of compound **1c**

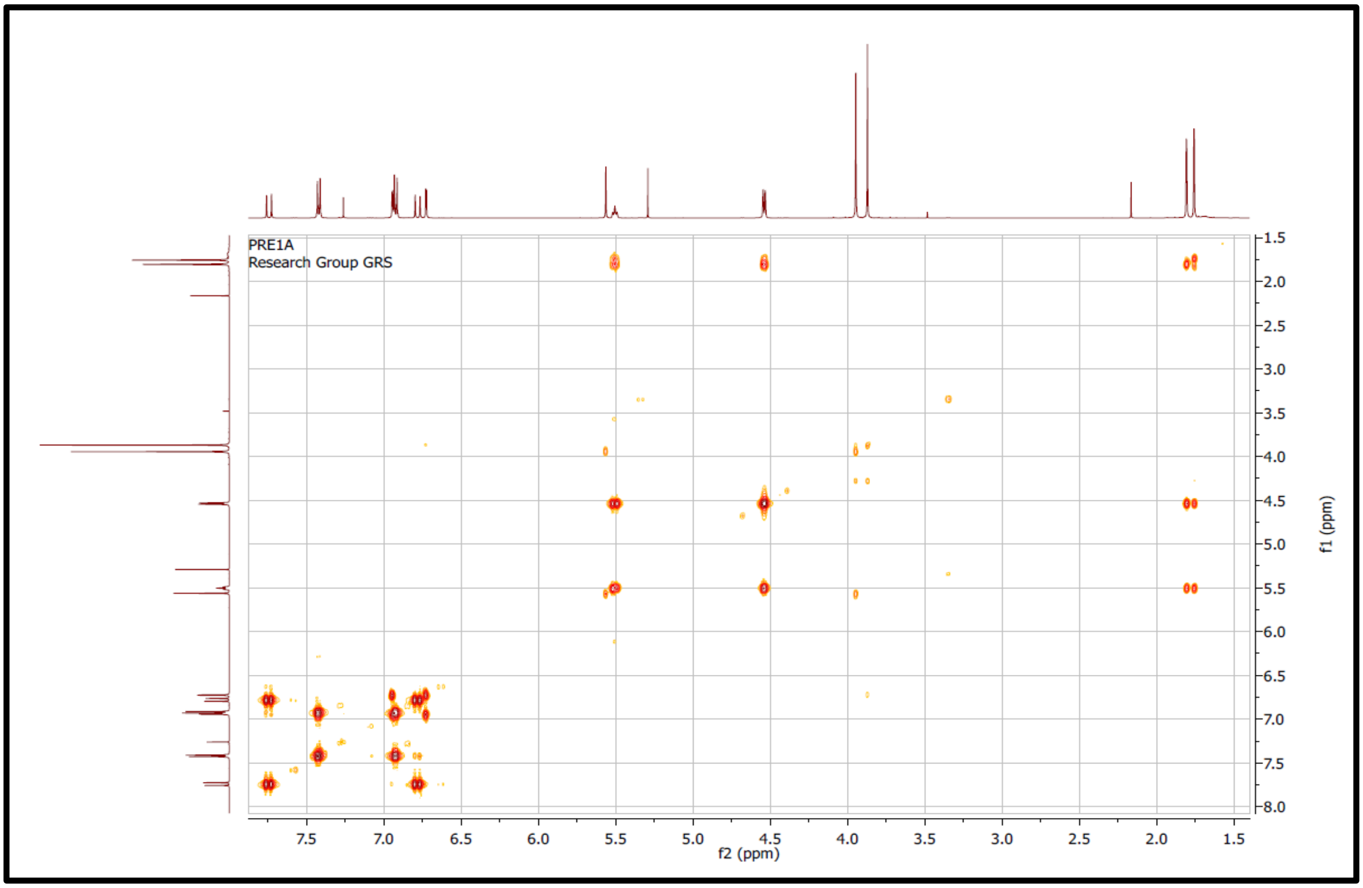

Figure S32: NOESY spectrum of compound **1c**

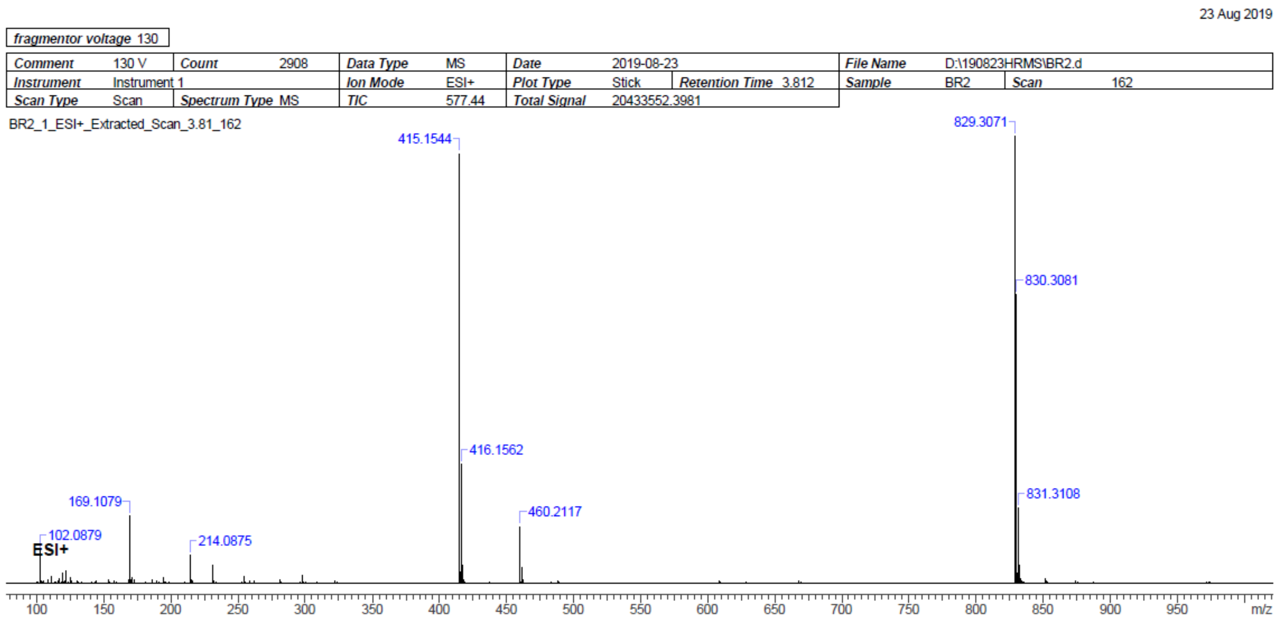

Expected Mass (*m/z*)	Observed Mass (*m/z*)	Error (ppm)
415.1545	415.1544	-0.240874

Figure S33: HRESIMS spectrum of compound **1d**

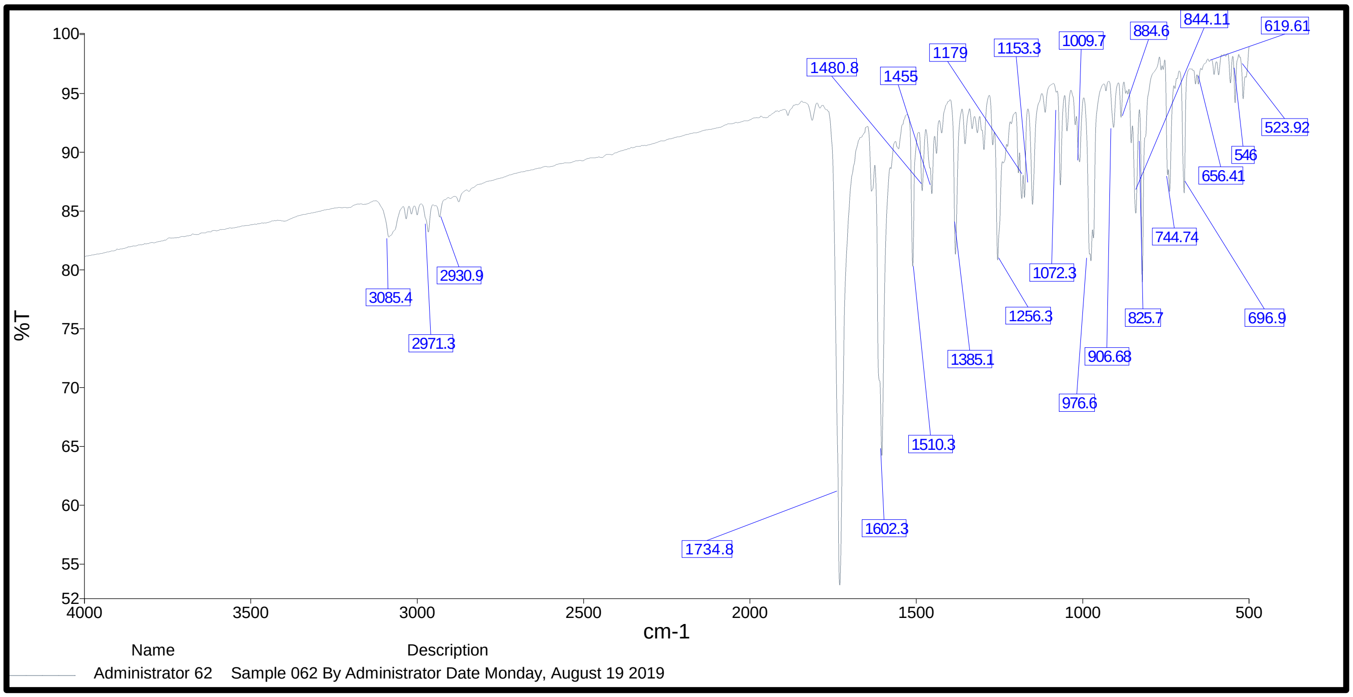

Figure S34: IR spectrum of compound **1d**

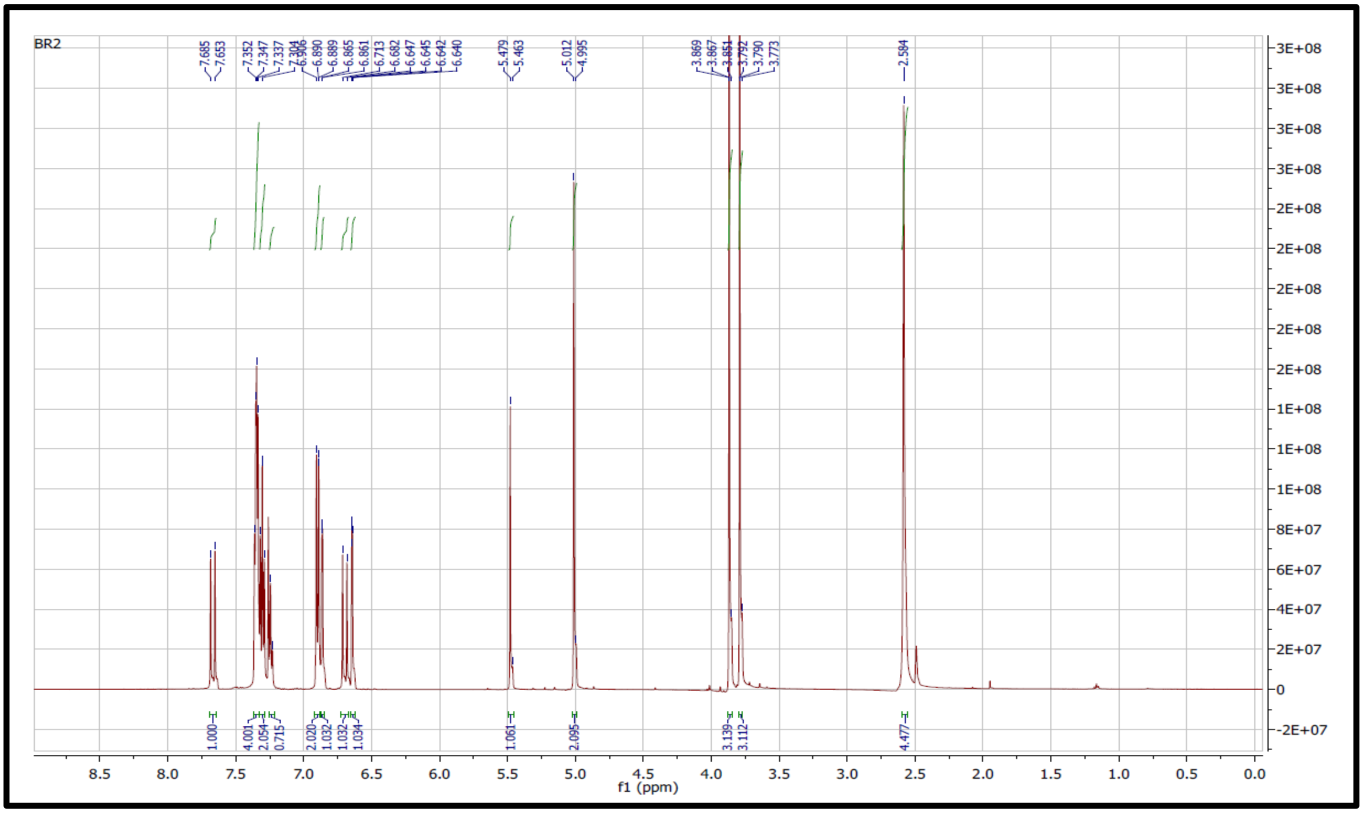

Figure S35: ^{1}H NMR spectrum ($CDCl_3$, 500 MHz) of compound **1d**

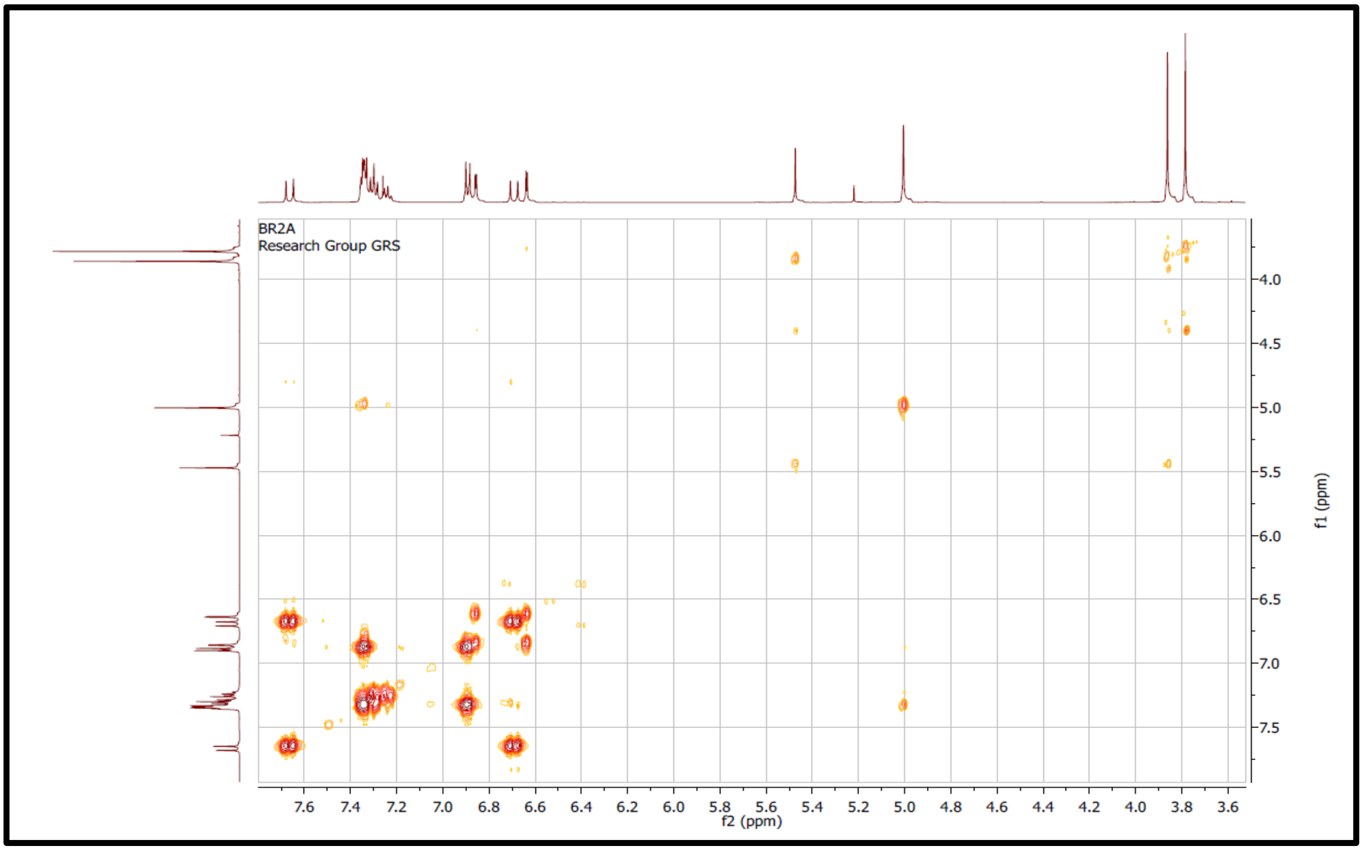

Figure S36: COSY spectrum of compound **1d**

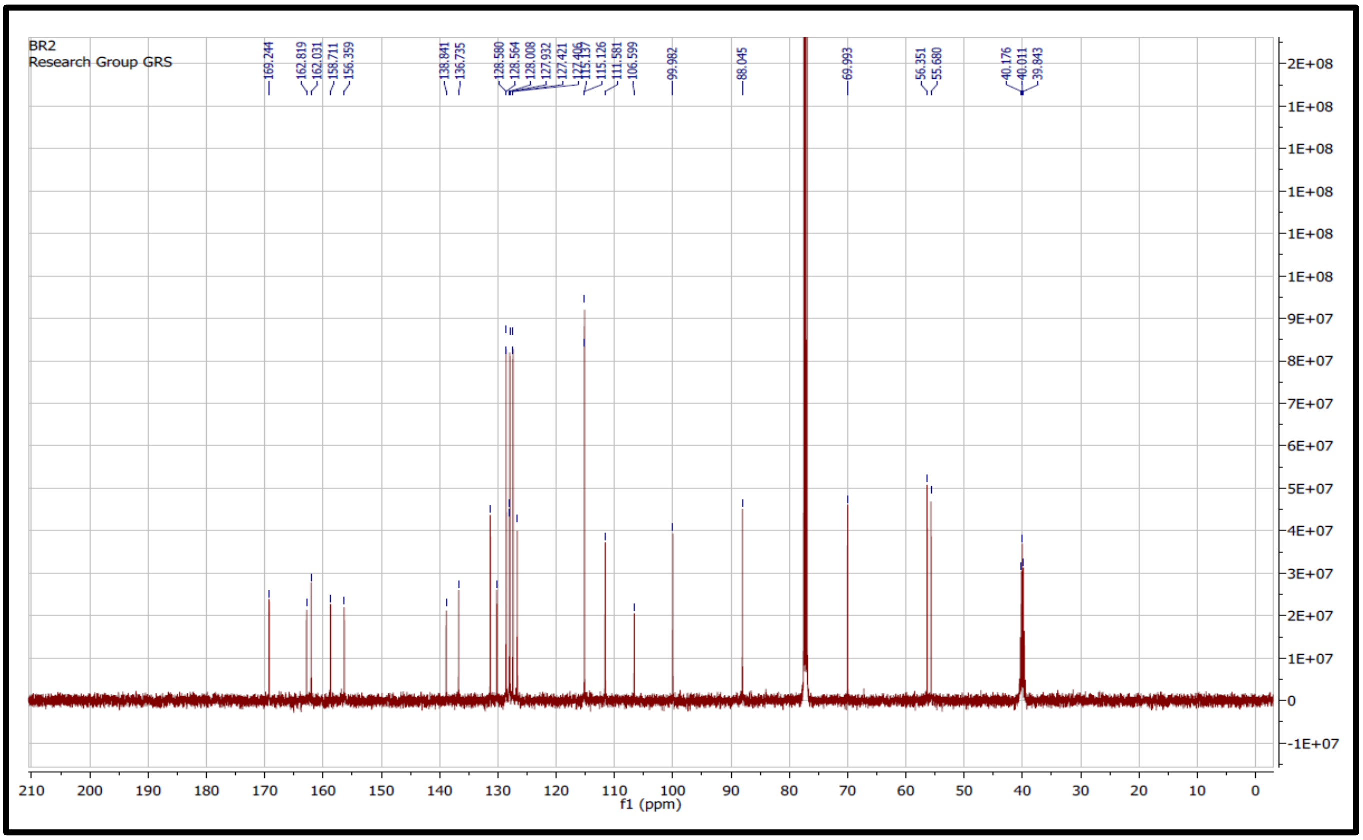

Figure S37: ^{13}C NMR spectrum ($CDCl_3$, 125 MHz) of compound **1d**

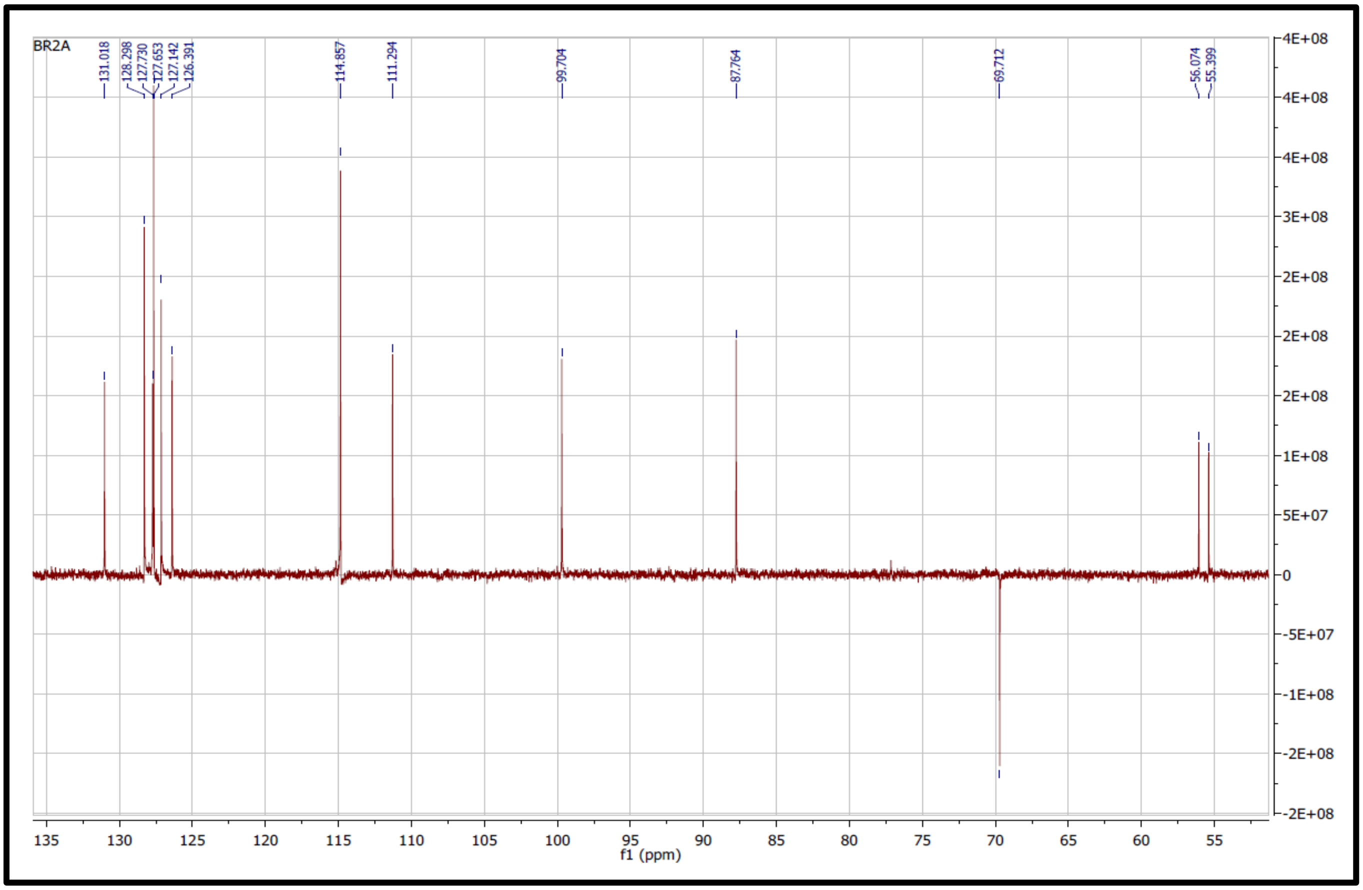

Figure S38: DEPT 135 spectrum of compound **1d**

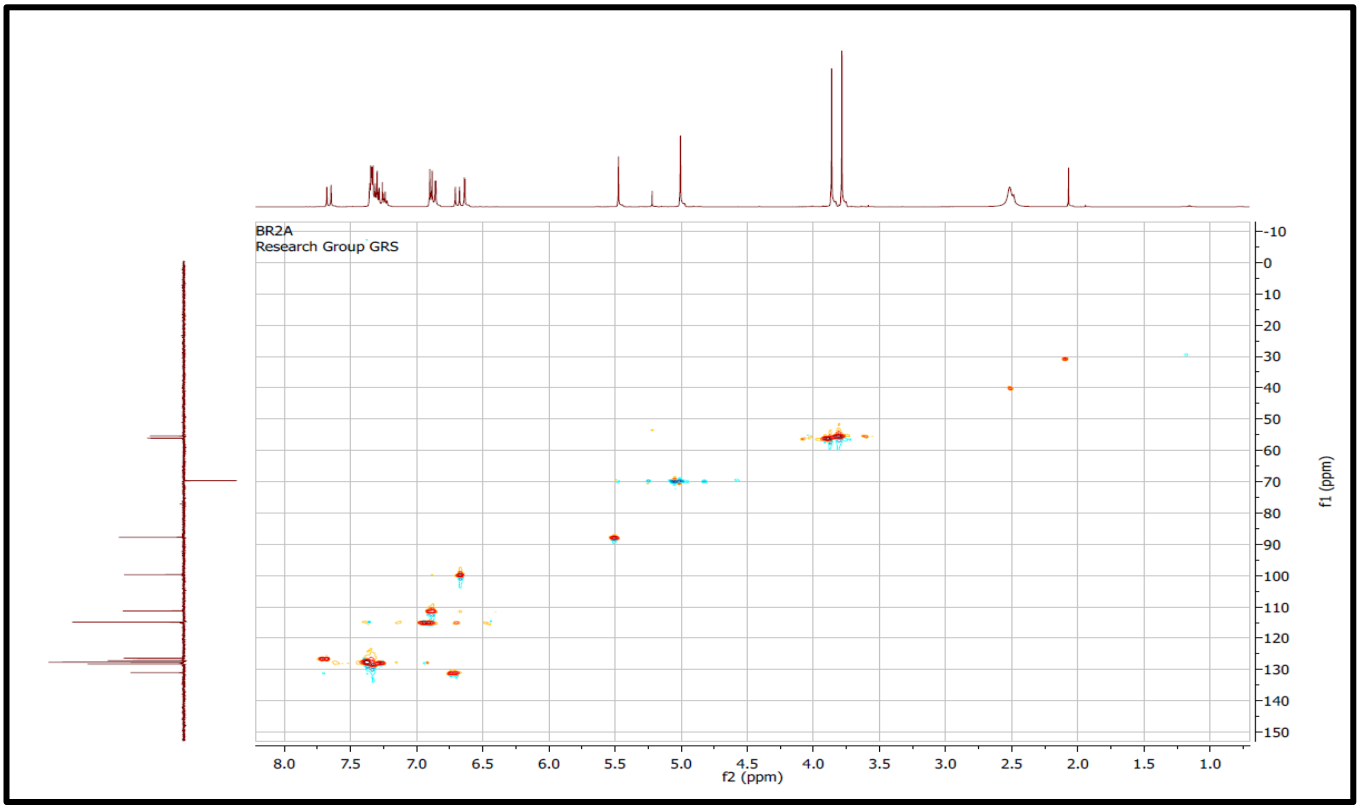

Figure S39: HSQC spectrum of compound **1d**

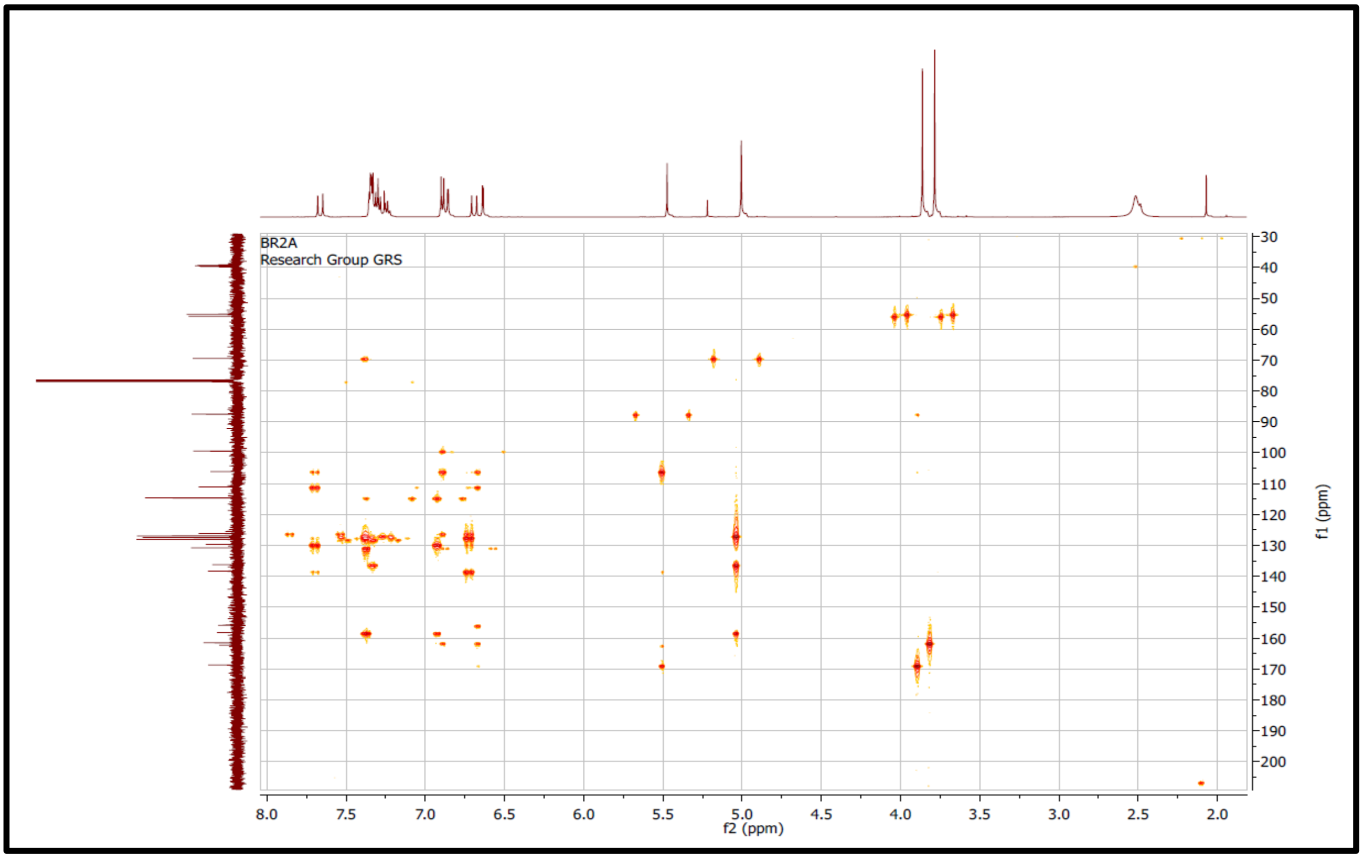

Figure S40: HMBC spectrum of compound **1d**

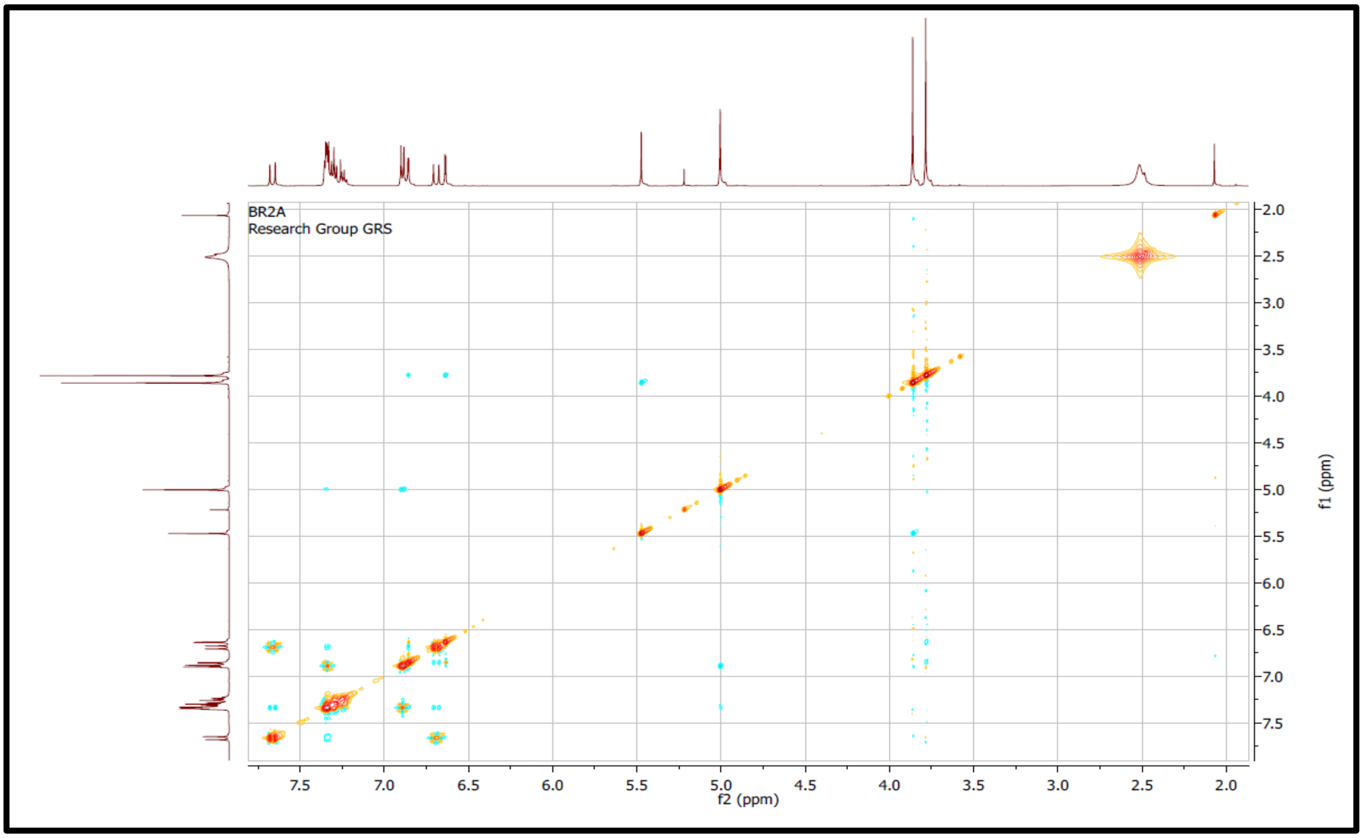

Figure S41: NOESY spectrum of compound **1d**

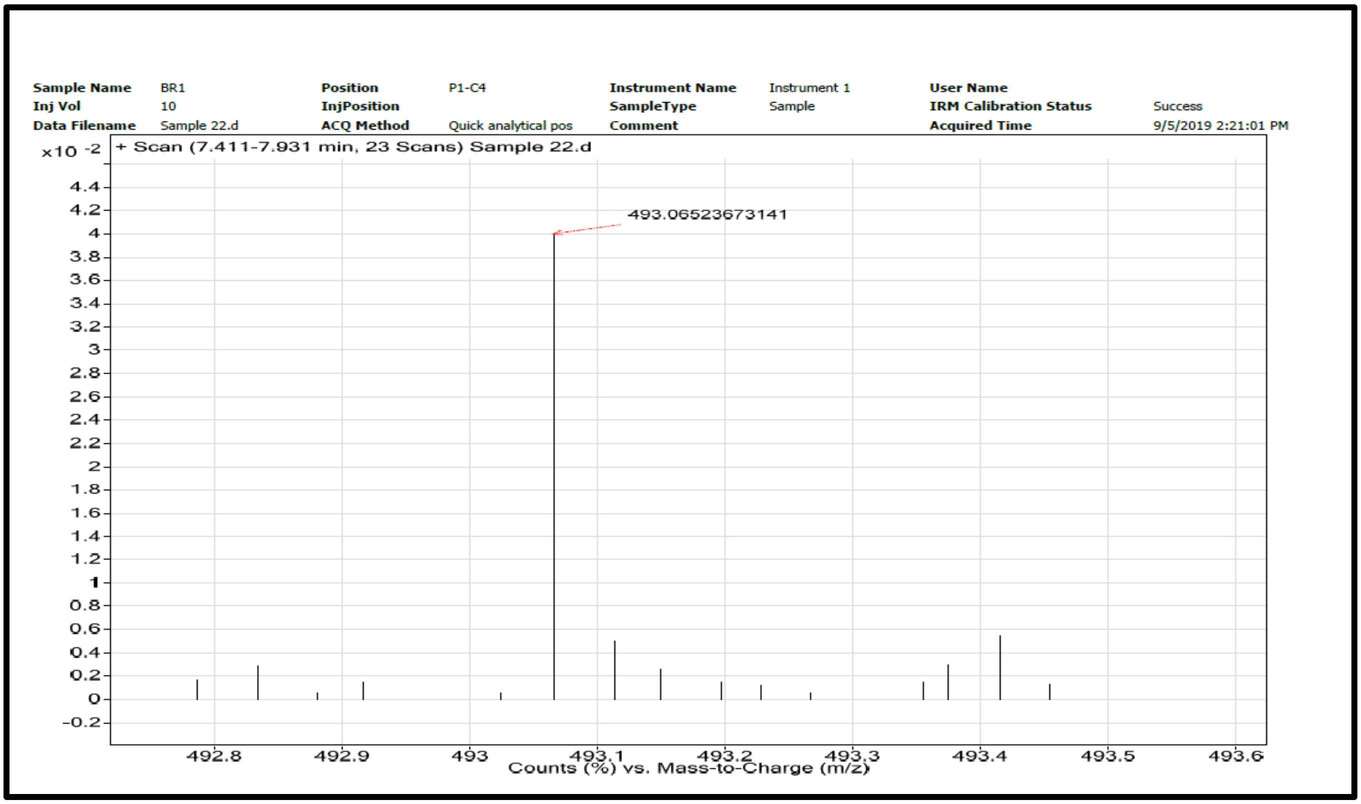

Figure S42: HRESIMS spectrum of compound **1e**

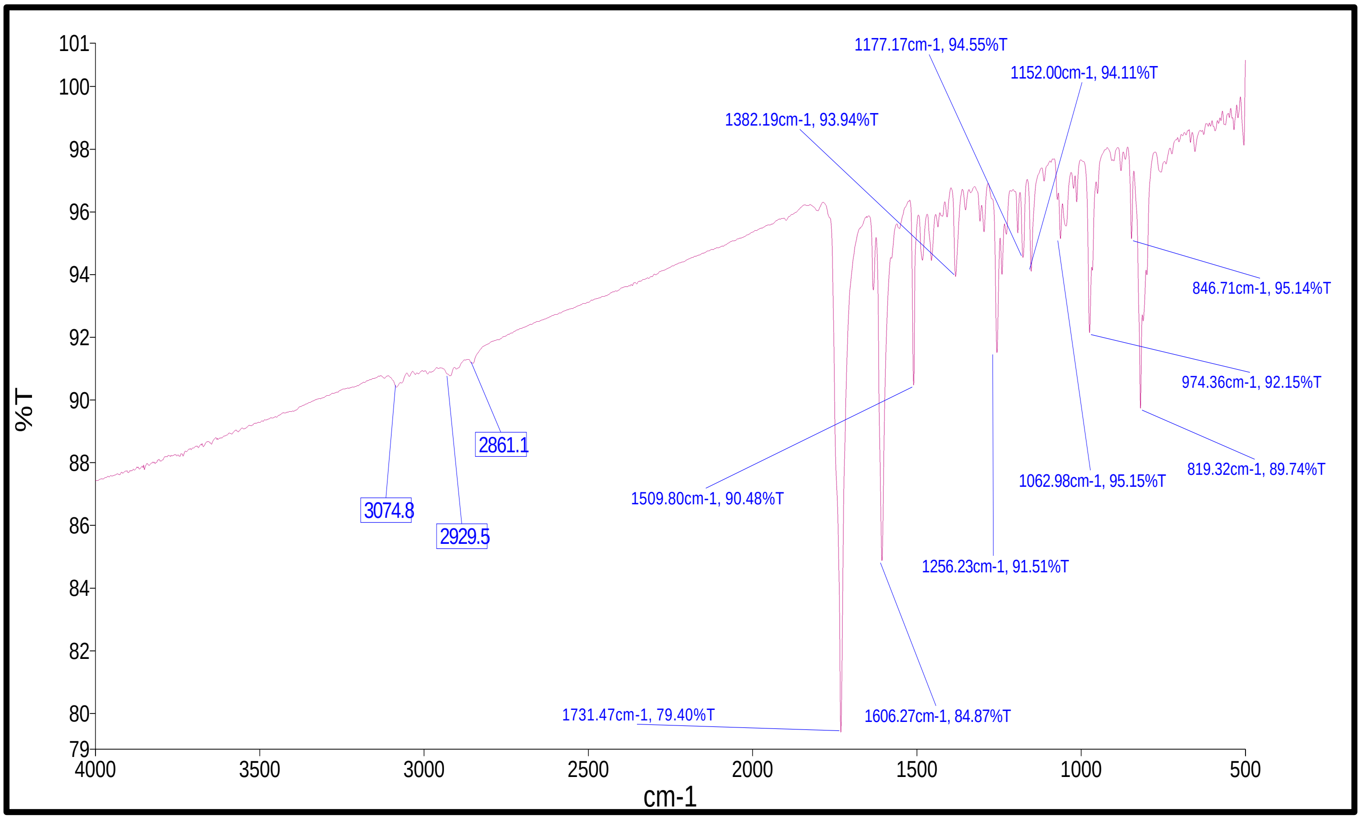

Figure S43: IR spectrum of compound **1e**

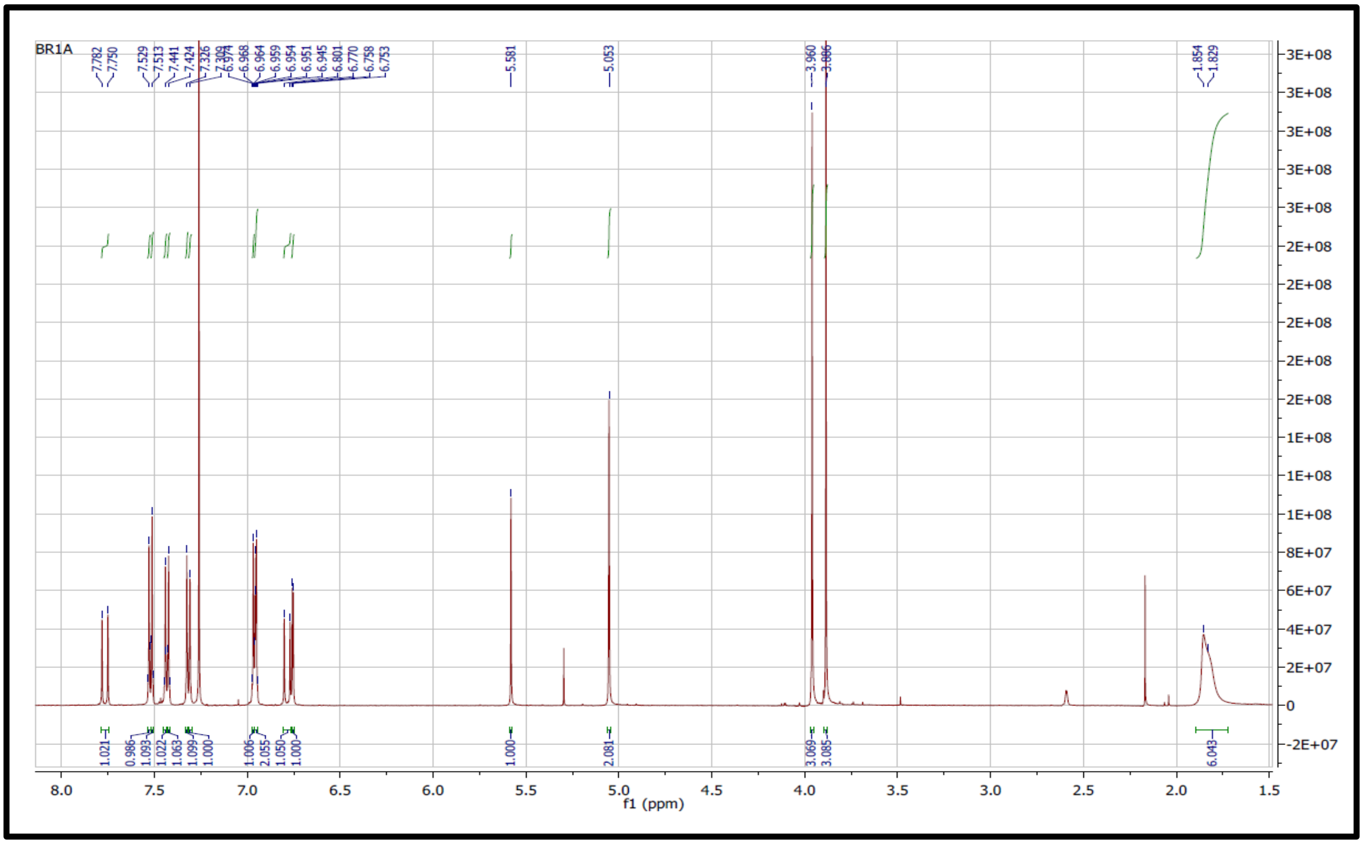

Figure S44: ^{1}H NMR spectrum ($CDCl_3$, 500 MHz) of compound **1e**

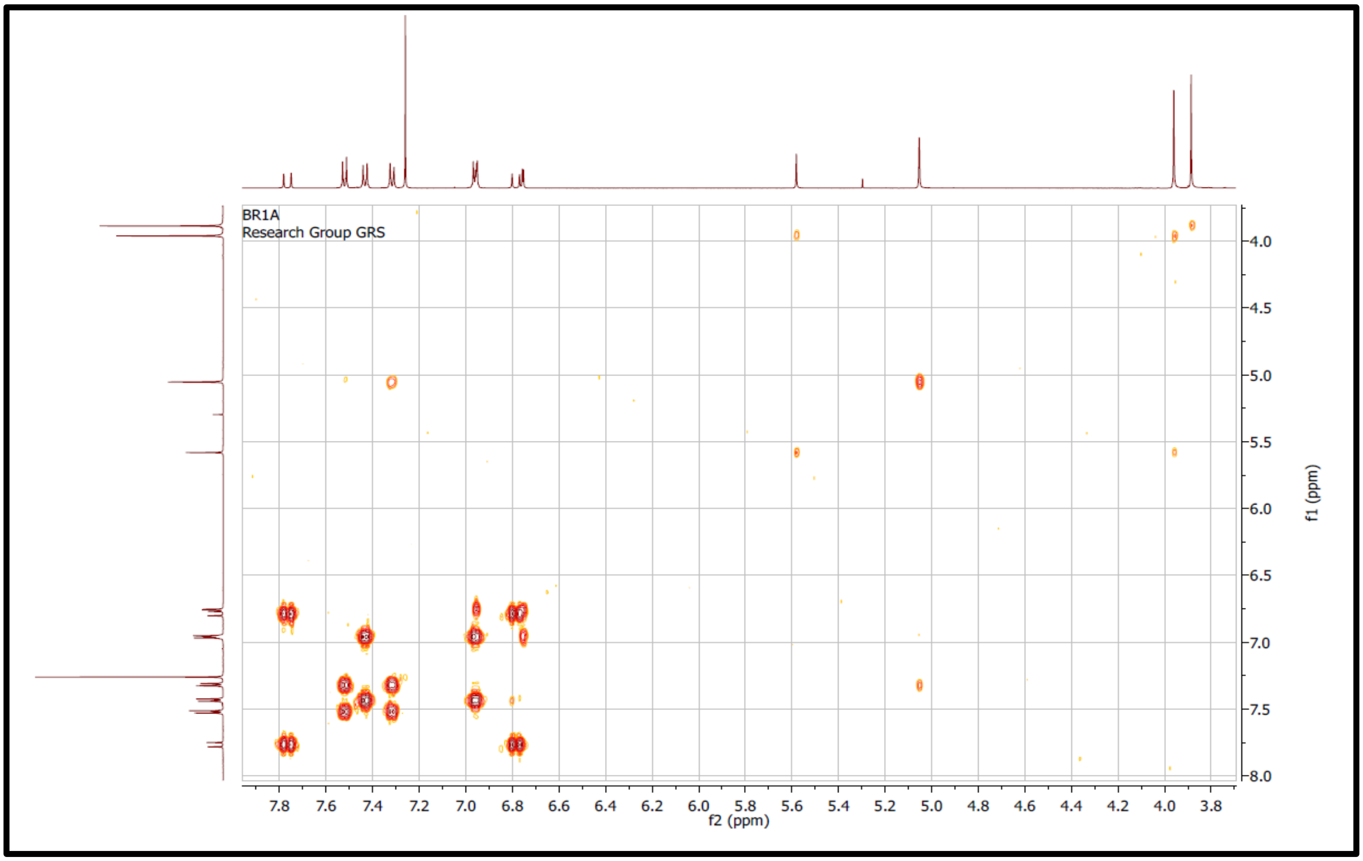

Figure S45: COSY spectrum of compound **1e**

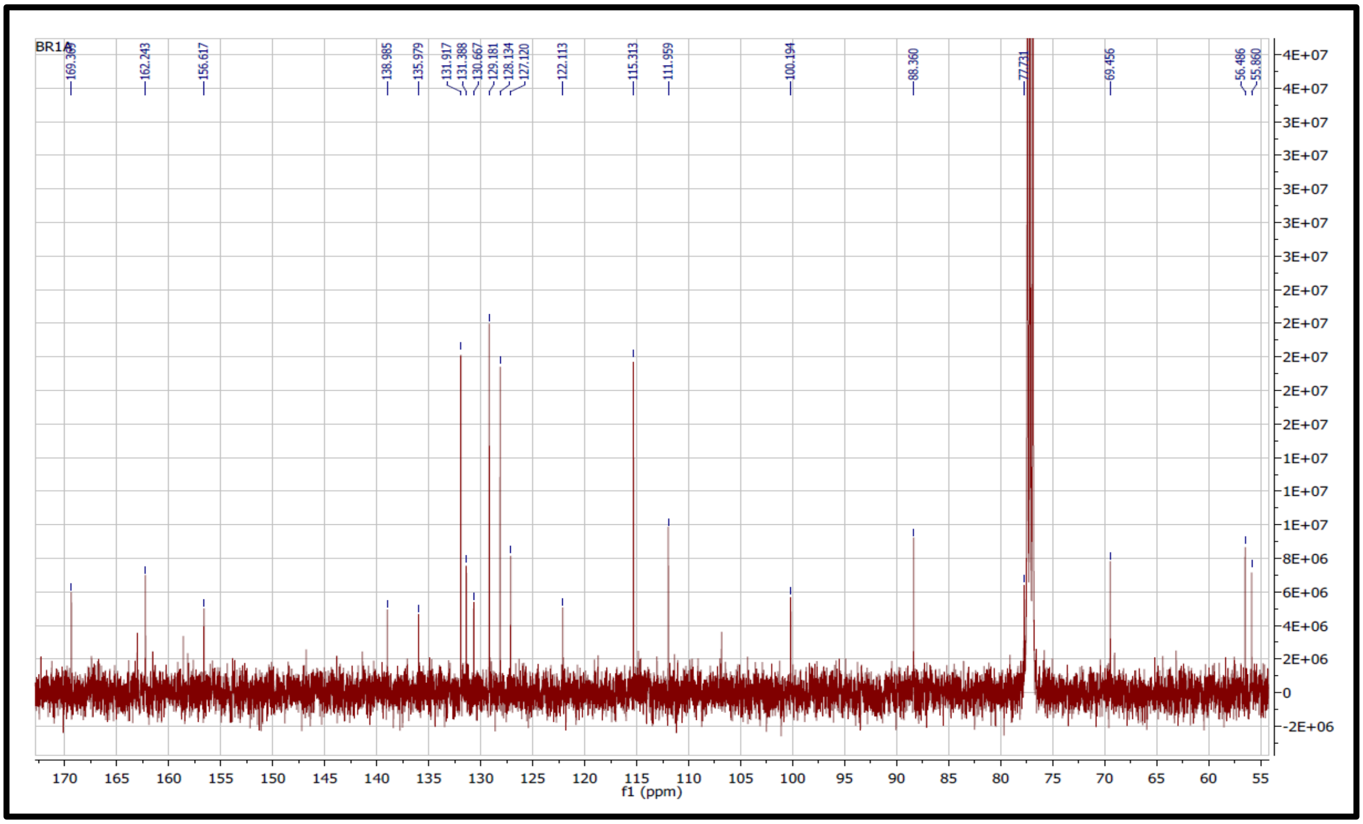

Figure S46: ^{13}C NMR spectrum ($CDCl_3$, 125 MHz) of compound **1e**

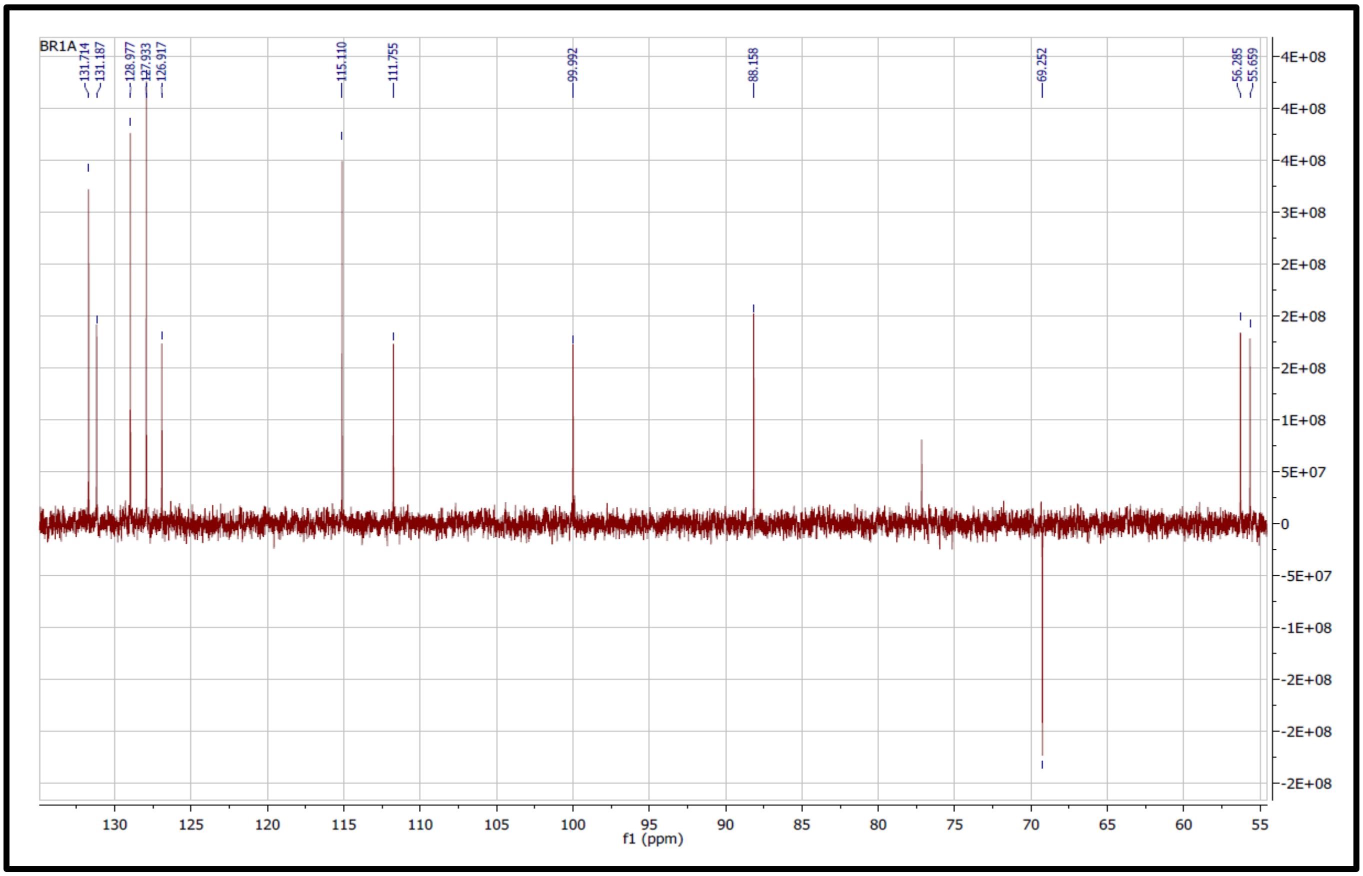

Figure S47: DEPT 135 spectrum of compound **1e**

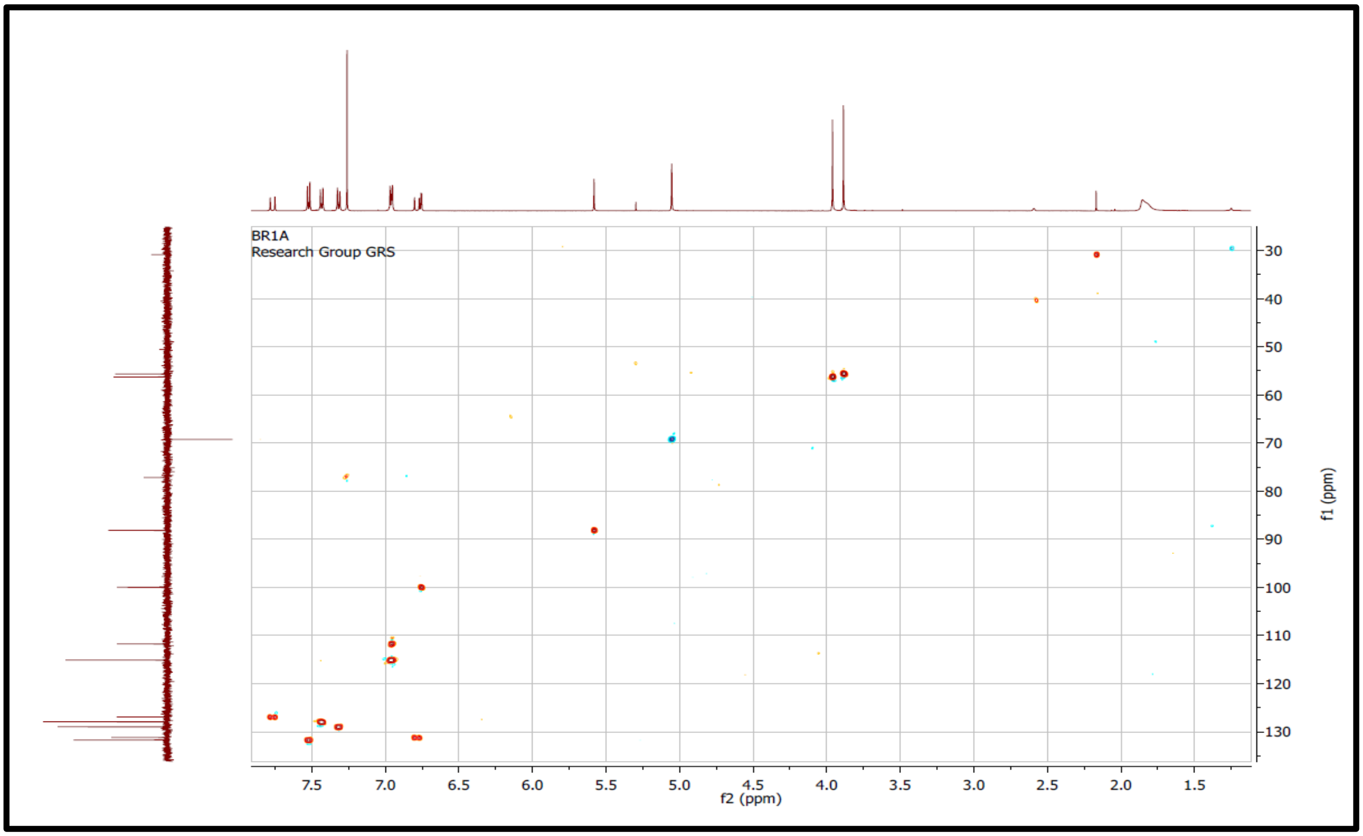

Figure S48: HSQC spectrum of compound **1e**

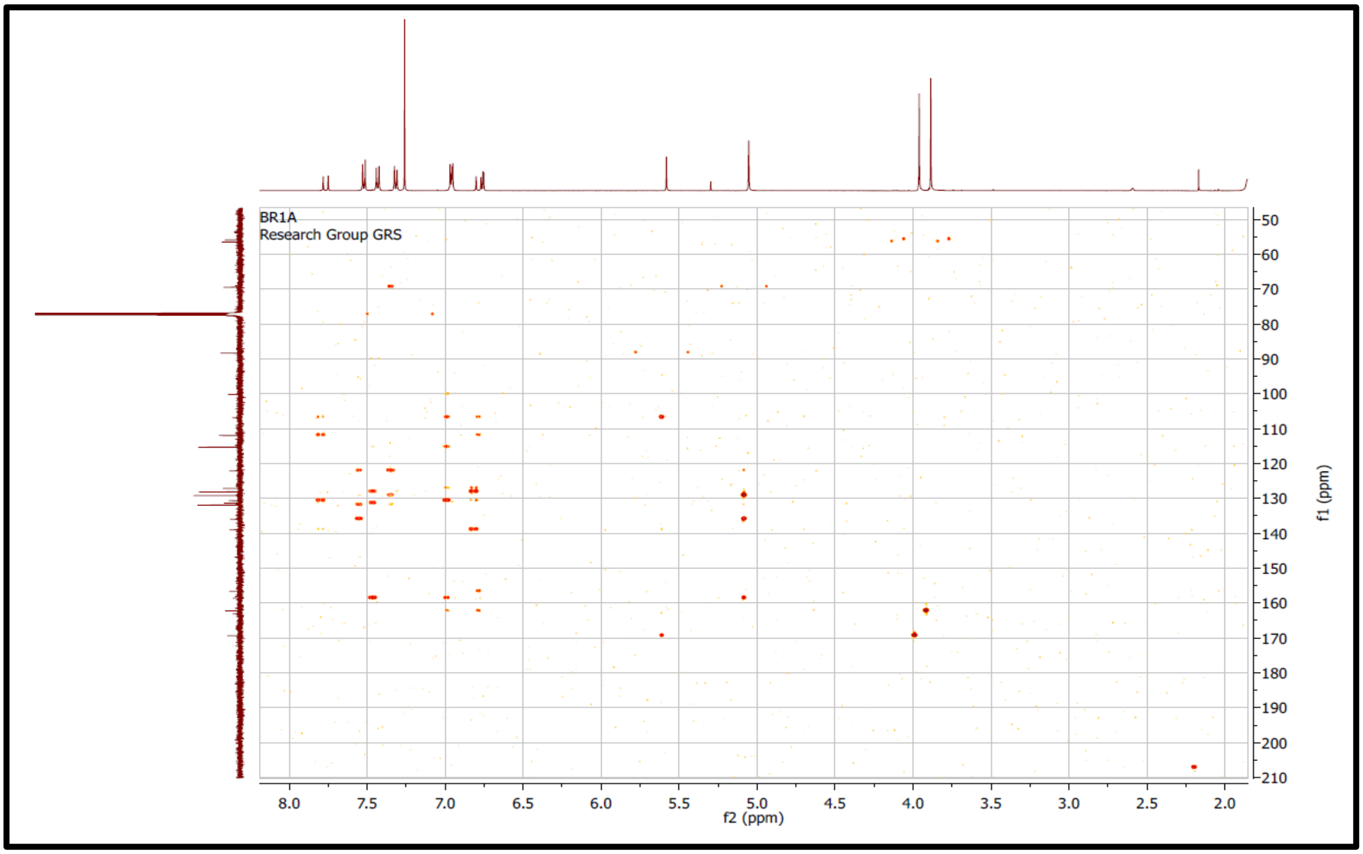

Figure S49: HMBC spectrum of compound **1e**
